# Thiazol-4-yl-Methylthio-Quinazolin-4(3H)-ones as Anticonvulsant Compounds: Chemical Design, Computational Studies, and Biological Evaluation

**DOI:** 10.3390/ijms27146107

**Published:** 2026-07-08

**Authors:** Daniel Ungureanu, Anamaria Apan, Cristina Mogoșan, Radu Tamaian, Brîndușa Tiperciuc, Gabriel Marc, Raluca Pele, Laurian Vlase, Adrian Pîrnău, Cristina Moldovan, Ioana Ionuț, Anca Stana, Ovidiu Oniga

**Affiliations:** 1 Department of Pharmaceutical Chemistry, Faculty of Pharmacy, “Iuliu Hațieganu” University of Medicine and Pharmacy, 41 Victor Babeș Street, 400012 Cluj-Napoca, Romania; daniel.ungureanu@elearn.umfcluj.ro (D.U.); btiperciuc@umfcluj.ro (B.T.); raluca.pele@umfcluj.ro (R.P.); cmoldovan@umfcluj.ro (C.M.); ionut.ioana@umfcluj.ro (I.I.); stana.anca@umfcluj.ro (A.S.); ooniga@umfcluj.ro (O.O.); 2 Department of Pharmacology, Physiology, Pathophysiology, Faculty of Pharmacy, “Iuliu Hațieganu” University of Medicine and Pharmacy, 6A Louis Pasteur Street, 400349 Cluj-Napoca, Romania; anamaria.apan@umfcluj.ro; 3 ICSI Business, National Institute for Research and Development for Cryogenic and Isotopic Technologies–ICSI Rm. Vâlcea, 4 Uzinei Street, 240050 Râmnicu Vâlcea, Romania; radu.tamaian@icsi.ro; 4 Department of Organic Chemistry, Faculty of Pharmacy, “Iuliu Hațieganu” University of Medicine and Pharmacy, 41 Victor Babeș Street, 400012 Cluj-Napoca, Romania; marc.gabriel@umfcluj.ro; 5 Department of Pharmaceutical Technology and Biopharmacy, Faculty of Pharmacy, “Iuliu Hațieganu” University of Medicine and Pharmacy, 41 Victor Babeș Street, 400012 Cluj-Napoca, Romania; laurian.vlase@umfcluj.ro; 6 National Institute for Research and Development of Isotopic and Molecular Technologies, 67–103 Donath Street, 400293 Cluj-Napoca, Romania; apirnau@itim-cj.ro

**Keywords:** thiazole, quinazolin-4(3*H*)-one, hybrid compounds, anticonvulsant

## Abstract

The purpose of this study was the chemical design, synthesis, and evaluation of the anticonvulsant potential of 15 novel thiazolyl-methylthio-quinazolin-4(3*H*)-one hybrid compounds (**4a-o**). The compounds were designed based on a scaffold that reunited thiazole and quinazolin-4(3*H*)-one heterocycles of two well-known anticonvulsants, clomethiazole and methaqualone, through a condensation reaction. The compounds were evaluated in vivo for anticonvulsant activity using the pentylenetetrazole-induced seizure animal model. A Rotarod test was employed to evaluate the neuromotor coordination after the administration of the tested compounds, and a flumazenil antagonism assay was subsequently performed to investigate if the observed anticonvulsant effects were mediated through the compounds’ interaction with the GABA_A_ receptor. The in silico assessment consisted of molecular docking, evaluation of the druggability, and ADMETox prediction. All compounds presented anticonvulsant activity to varying degrees. The most notable activity was observed in compounds **4k** (ED_50_ = 84.313 mg/kg) and **4c** (ED_50_ = 178.165 mg/kg). The in vivo results positively correlated with the observations drawn in the molecular docking study on the human α_1_β_2_γ_2_ GABA_A_ receptor and on the NR1 ligand-binding core of the NMDA receptor. The potential of anticonvulsant activity was also supported by the druggability and ADMETox predictions that highlighted an increased possibility of brain–blood barrier permeation, supported by the computed parameters TPSA, logD, and logBB. The results of the flumazenil antagonism assay additionally highlighted the possible mechanism of action of compounds **4c** and **4k** as positive allosteric modulators of the GABA_A_ receptor. Preliminary evaluation confirmed the anticonvulsant potential of the tested compounds, with further testing being necessary for a better understanding and confirmation of the activity.

## 1. Introduction

Epilepsy is a chronic neurological disease characterized by an increased predisposition to generate unprovoked seizures. According to the International League Against Epilepsy (ILAE) classification, seizures are divided into focal, generalized, unknown, and unclassified based on their onset [1,2,3,4].

In terms of prevalence, epilepsy is considered the most common neurological disease, affecting almost 60 million people from all age categories. The incidence is higher in the extremities of age; the youngest (86/100,000/year cases in the first year of age) and the oldest populations (180/100,000/year cases in the over 85-year-old age group). The most common type of seizures are the focal onset seizures, both in children and in adult populations [1,5,6].

The seizures can also occur as a consequence of disturbances of structural brain integrity and brain function. In this case, they are known as acute symptomatic seizures. Examples of causes include cortical hemorrhage, alcohol and benzodiazepine withdrawals, strokes, traumatic brain injuries, hyponatremia, central nervous system (CNS) infections, etc. [7].

Antiseizure medications (ASMs) or anticonvulsants are used to prevent seizures in epilepsy and secondary to the previously mentioned conditions. Their mechanisms of action include all currently known targets implicated in epileptogenesis: glutamate and γ-aminobutyric acid (GABA) neurotransmissions, sodium, potassium, and calcium channels, etc. Additionally, some ASMs (carbamazepine, valproic acid, lamotrigine, and topiramate, with gabapentin and pregabalin used as add-on therapies) have shown efficacy in both manic and depressive episodes of bipolar disorder, where they are used as mood stabilizers. Moreover, they can be used as off-label medications to treat impulsivity and aggressiveness in patients with schizophrenia and schizoaffective disorder, as well as to control impulsivity and craving in alcohol and substance use disorders. To further emphasize their polyvalent activities, they can also be used as add-on therapies in treatment-resistant panic disorder and neuropathic pain [8,9,10,11,12,13,14].

Despite the large number of available ASMs, approximately 30% of patients do not respond to monotherapy and may need combination therapy, ultimately leading to drug-resistant epilepsy (DRE). DRE is considered when two ASM schedules (monotherapy or combinations) fail to achieve seizure control [8,15,16,17,18,19].

Based on the mentioned limitations regarding ASMs, there is an ongoing interest in designing novel anticonvulsants with a broader activity range and a better safety profile [20,21,22,23,24].

Quinazoline-4(3*H*)-one and thiazole are two heterocycles found in compounds with documented anticonvulsant activity or potential. Quinazoline-4(3*H*)-one is the centerpiece of methaqualone (Quaalude^®^), a non-benzodiazepine γ-aminobutyric acid type A (GABA_A_) receptor positive allosteric modulator (PAM), which was retracted due to increasing recreational use (“disco biscuits”). Other quinazoline-4(3*H*)-one derivatives are generally known as “quaaludes” and include mebroqualone, mecloqualone, etaqualone, diproqualone, chloroqualone, nitrometaqualone, afloqualone, and methylmethaqualone [22,25,26,27,28,29,30,31,32,33,34,35]. Moreover, the quinazolin-4(3*H*)-one heterocycle has been found in compounds with antagonist activity on the *N-*methyl-*D*-aspartate (NMDA) receptors, thus reducing glutamate hyperactivity [36,37].

Thiazole is the main structural element of clomethiazole, another GABA_A_ receptor PAM, which was used for the treatment of alcohol withdrawal. Additionally, several other experimental compounds containing the mentioned heterocycles have been designed and reported in the literature with promising anticonvulsant activity and reduced risk of neurotoxicity [38,39,40].

**Figure 1:**
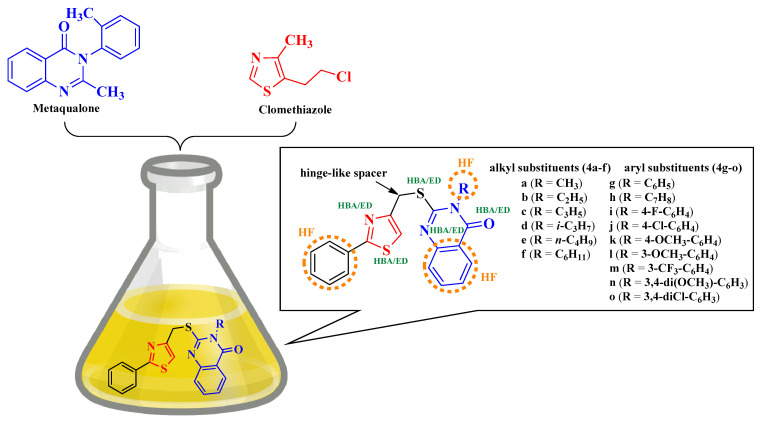
Chemical design and general presentation of compounds **4a-o**. The compounds were designed starting from methaqualone (containing the quinazoline-4(3*H*)-one heterocycle) and clomethiazole (containing the thiazole heterocycle). Legend: HF = hydrophobic, HBA/ED = hydrogen bond acceptor/electron donor, *n* = normal, and *i* = iso.

Encouraged by the available literature data regarding the quinazoline-4(3*H*)-one- and thiazole-containing anticonvulsant compounds, especially by the chemical structures of methaqualone and clomethiazole, we hypothesized that the molecular hybridization of these two heterocycles into a single scaffold could generate novel anticonvulsant candidates. The designed thiazolyl-methylthio-quinazolin-4(3*H*)-one hybrids were conceived to integrate three hydrophobic (HF) regions intended to support hydrophobic and π-π stacking interactions within receptor binding sites, several hydrogen bond acceptor/electron donor (HBA/ED) atoms capable of anchoring the ligands through polar interactions, and a methyl-thio linker as a hinge-like spacer between the two heterocycles (Figure 1).

The purpose of this study was the design and evaluation of novel thiazol-4-yl-methylthio-quinazolin-4(3*H*)-one hybrid compounds as potential PAMs of GABA_A_ receptor and NMDA receptor antagonists with anticonvulsant activity.

## 2. Results

### 2.1. Chemical Synthesis

**Figure 2:**
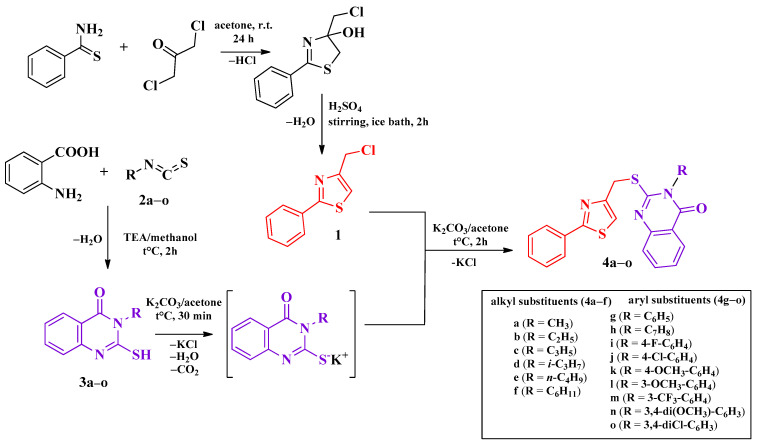
The general synthetic pathway for compounds **4a-o**.

Fifteen (2-phenylthiazol-4-yl)methyl)thio)quinazolin-4(3*H*)-one compounds (**4a-o**) were obtained through the condensation of the 2-phenyl-thiazole-4-yl-chloromethyl (**1**) intermediate with the corresponding 3-alkyl/aryl-2-mercaptoquinazolin-4(3*H*)-one intermediates (**3a-o**). The compounds were divided into two series based on the nature of the substituent (alkyl—**4a-f** or aryl—**4g-o**) from position 3 of the quinazoline-4(3*H*)-one ring (Figure 2).

The novel compounds were obtained in different yields ranging from 52.21% to 88.99%, while their structural identity was confirmed through IR, MS, ^1^HNMR, and ^13^CNMR spectral analysis. TLC and retardation factors (R_f_) were used to monitor the reaction progress.

The graphical presentations of the IR, MS, ^1^H-NMR, and ^13^C-NMR spectra for all intermediate and final compounds are available in the Supplementary Files (Figures S1–S90 from the Supplementary Materials).

### 2.2. Computational Studies

#### 2.2.1. Druggability and ADMETox Prediction Studies

The 3D-optimized structures of all compounds used in the ADMETox prediction study are available in the Supplementary Files (Table S1 from the Supplementary Materials).

The physicochemical descriptors taken into consideration for the druggability prediction of compounds **4a-o** were the molecular weight (MW), the number of rotatable bonds (RBs), the number of H-bond acceptors (HBAs), the number of H-bond donors (HBDs), the topological polar surface area (TPSA), the octanol–water partition coefficient implemented by Moriguchi (MLogP), logD at pH = 7.4, logBB, the estimated solubility (ESOL), and the number of violations of Lipinski’s rule of five [41,42,43,44,45,46].

The pharmacokinetic descriptors taken into consideration for this study were gastrointestinal (GI) absorption, P-glycoprotein (P-gp) inhibitor and substrate, blood–brain barrier (BBB) penetration, inhibition potential of several cytochrome P450 isoenzymes (CYP1A2, 2C19, 2C9, 2D6, and 3A4), and half-life (T_1/2_).

The toxicological descriptors taken into consideration for this study were carcinogenesis, eye irritation, hepatotoxicity, respiratory toxicity, skin sensitization, rat acute toxicity, and rat chronic oral toxicity.

Table 1, Table 2 and Table 3 summarize the predicted results for the druggability and ADMETox simulations performed with SwissADME and Deep-PK.

#### 2.2.2. Molecular Docking Studies

The molecular docking study was performed on the human α_1_β_2_γ_2_ GABA_A_ receptor (PDB entry code: 6X3Z) and on the NR1 ligand-binding core of the NMDA receptor (PDB entry code: 1PBQ). The selection of two targets from opposite neurotransmitters was motivated by the fact that many anticonvulsants have several pharmacological mechanisms to exert their activity (the so-called “magic shotgun” compounds with affinity for multiple targets). Examples of authorized anticonvulsants with both glutamatergic and GABAergic mechanisms include topiramate, valproic acid, and zonisamide [47,48,49,50]. Therefore, the selection of multiple targets for molecular docking increases the chances of proper in silico–in vivo correlations.

The results of the predicted binding affinity (BA) of compounds **4a-o** to the GABA_A_ and NMDA receptors are presented in Table 4.

**Figure 3:**
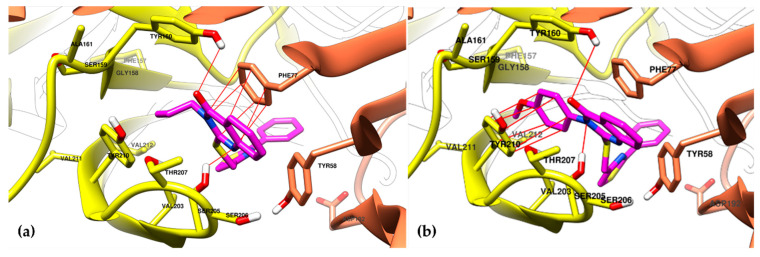
The predicted binding pose in the human α_1_β_2_γ_2_ GABA_A_ receptor of (**a**) compound **4c**; (**b**) compound **4k**. The following coloring scheme was used: purple for carbon atoms, red for oxygen atoms, blue for nitrogen atoms, white for hydrogen atoms, and yellow for sulfur atoms.

**Figure 4:**
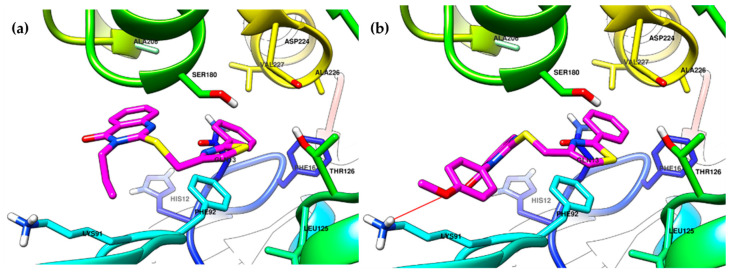
The predicted top binding conformation in the NR1 ligand-binding core of the NMDA receptor of (**a**) compound **4c**; (**b**) compound **4k**. The following coloring scheme was used: purple for carbon atoms, red for oxygen atoms, blue for nitrogen atoms, white for hydrogen atoms, and yellow for sulfur atoms.

The graphical depictions of the interaction between compounds **4c** and **4k**, which were the compounds that performed the best in the in vivo assay, with the human α_1_β_2_γ_2_ GABA_A_ receptor and the NR1 ligand-binding core of the NMDA receptor are presented in Figure 3 and Figure 4.

The graphical depictions of the interaction between compounds **4g**, **4h**, and **4i** with the human α_1_β_2_γ_2_ GABA_A_ receptor are presented in Figures S91–S93 from the Supplementary Materials.

### 2.3. Biological Evaluation

#### 2.3.1. Anticonvulsant Activity and Neuromotor Coordination Impairment

The percentages of mice protected against PTZ and the percentages of mice with preserved neuromotor coordination, following the i.p. administration of compounds **4a-o** in a single dose (D = 300 mg/kg), diazepam (D = 2 mg/kg), phenobarbital (D = 15 mg/kg), and for the negative control (NC) group are presented in Table 5.

**Figure 5:**
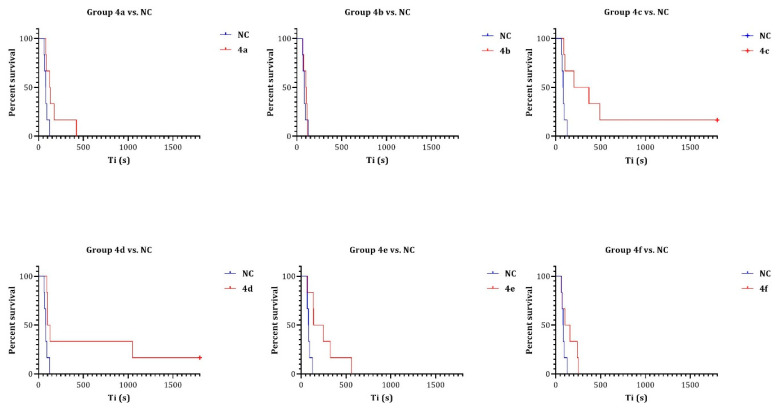
Kaplan–Meier time-to-seizure-onset survival analysis of compounds **4a-f** versus NC (negative control). Each panel represents the comparison of an individual compound versus NC (*n* = 6 mice). Blue lines represent the NC group, while red lines represent the tested compound. The “+” symbol marks censored observations (mice that did not present seizure by the 1800 s observation cutoff). Curve comparison was performed using the log-rank (Mantel–Cox) test, with Bonferroni correction for multiple comparisons.

**Figure 6:**
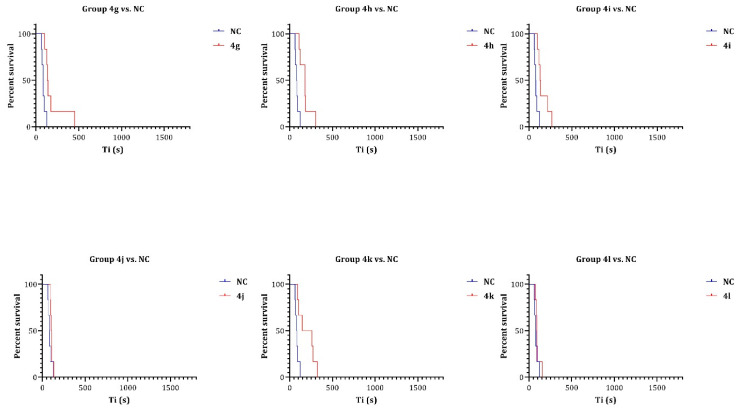
Kaplan–Meier time-to-seizure-onset survival analysis of compounds **4g-l** versus NC (negative control). Each panel represents the comparison of an individual compound versus NC (*n* = 6 mice). Blue lines represent the NC group, while red lines represent the tested compound. The “+” symbol marks censored observations (mice that did not present seizure by the 1800 s observation cutoff). Curve comparison was performed using the log-rank (Mantel–Cox) test, with Bonferroni correction for multiple comparisons.

**Figure 7:**
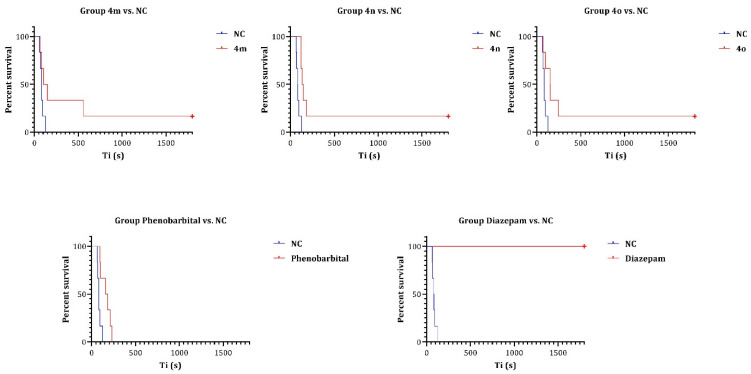
Kaplan–Meier time-to-seizure-onset survival analysis of compounds **4m-o** and reference drugs (phenobarbital and diazepam) versus NC (negative control). Each panel represents the comparison of an individual compound versus NC (*n* = 6 mice). Blue lines represent the NC group, while red lines represent the tested compound. The “+” symbol marks censored observations (mice that did not present seizure by the 1800 s observation cutoff). Curve comparison was performed using the log-rank (Mantel–Cox) test, with Bonferroni correction for multiple comparisons.

**Figure 8:**
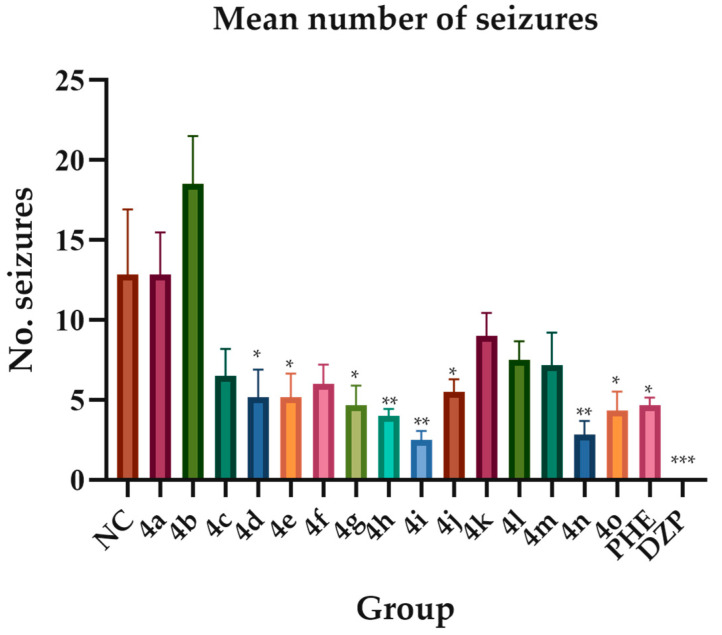
Mean number of seizures. For the statistically significant values compared to the negative group, the *p* value was expressed as *** *p* < 0.001; ** *p* < 0.01; * *p* < 0.05.

The comparisons between the groups for the latency until the first seizure (Ti) and the mean number of seizures are illustrated in Figure 5, Figure 6, Figure 7 and Figure 8. Data regarding each of these parameters are available in the Supplementary Files (Tables S2–S4 from the Supplementary Materials).

Based on the results from the initial screening, compounds **4c** and **4k** emerged as the most potent anticonvulsants and were further tested to determine their ED_50_. Table 6 summarizes the percentages of mice protected against PTZ-induced seizures and those with preserved neuromotor coordination, following the administration of compounds **4c** and **4k** at four different doses (D_1_ = 50 mg/kg, D_2_ = 150 mg/kg, D_3_ = 300 mg/kg, and D_4_ = 450 mg/kg), alongside the negative control (NC) group. Based on the percentages of mice protected against PTZ, the ED_50_ values were determined for both compounds.

#### 2.3.2. Flumazenil Antagonism Assay

**Figure 9:**
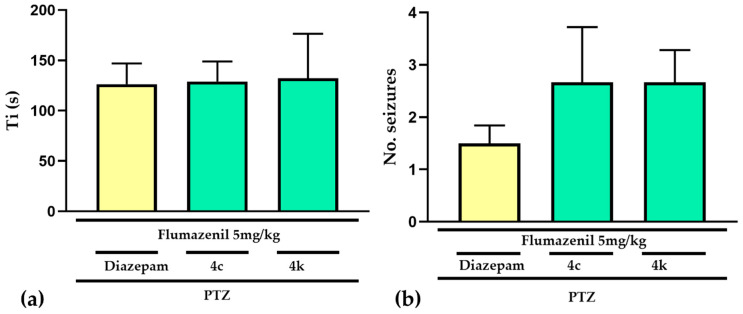
Results obtained in the flumazenil antagonism assay for compounds **4c** and **4k** in comparison to diazepam in terms of (**a**) latency until the first seizure (Ti); (**b**) number of seizures. For the statistically significant values compared to the diazepam group, the *p* value was expressed as *** *p* < 0.001; ** *p* < 0.01; * *p* < 0.05.

The comparisons between the groups for the latency until the first seizure (Ti) and the mean number of seizures are illustrated in Figure 9. Data regarding each of these parameters are available in the Supplementary Files (Table S5 from the Supplementary Materials).

## 3. Discussion

### 3.1. Chemical Synthesis

All intermediate compounds **1** and **3a-o** were previously reported in the literature [51,52,53,54,55,56,57,58,59,60,61,62]. The final products **4a-o** were not reported, according to the Reaxys database.

The lower yield obtained for compound **4o** compared with **4g** may be attributed primarily to the stronger electron-withdrawing nature of the 3,4-dichlorophenyl substituent, as aromatic meta and para substituents mainly exert electronic effects on the reactivity. In addition, the chlorinated aryl group may modify the physicochemical properties of the product, including solubility and crystallization behavior, which may lead to increased material losses during isolation and recrystallization [63,64].

The structures of final products **4a-o** were confirmed by spectral analysis. According to the IR spectra, all compounds showed νN-H wide stretching bands between 3413.39 cm^−1^ and 3450.51 cm^−1^, the first νC=O ketone band of the amide group between 1662.20 cm^−1^ and 1693.19 cm^−1^, the second νC=O ketone band between 1602.56 cm^−1^ and 1622.93 cm^−1^, νC=N between 1538.92 cm^−1^ and 1550.01 cm^−1^, and νC-N between 1468.53 cm^−1^ and 1508.06 cm^−1^. According to the MS, all corresponding molecular peaks were identified. Finally, according to the ^1^H- and ^13^C-NMR spectra, all the expected proton signals and signals for the carbon atoms were identified in the corresponding regions of the spectra with the expected multiplicity.

### 3.2. Druggability and ADMETox Prediction Studies

Compounds **4a-e**, **4k**, **4l**, and **4n** had no violations of Lipinski’s rule of five. They had a molecular weight lower than 500 g/mol, the number of HBAs was less than 10, while the number of HBDs was less than 5, and the value of MLogP was lower than 4.15. Compounds **4f-j**, **4m**, and **4o** had one violation of Lipinski’s rule of five, due to an exceeding value of MLogP [44,45]. It can be considered that compounds **4a-o** have good druggability properties, since the violation of one rule is accepted, as suggested by other publications [65,66].

By increasing the length of the alkyl substituent, the number of RBs increased as well, as observed in compounds **4a-c** and **4e** (Table 1). Grafting methoxy groups on the aromatic-substituted compounds **4g-o** increased the number of HBAs and TPSAs for compounds **4k**, **4l**, and **4n** (Table 1). Similarly, the number of HBAs also increased in fluorinated compounds **4c** and **4m**. MLogP values were higher in the aromatic series **4g-o** compared to the aliphatic ones **4a-f** (Table 1). Compounds **4a-e** were predicted to be moderately soluble (ESOL > −6.00), while the rest of the compounds were poorly soluble (ESOL > −7.00). All compounds had a TPSA value below 140 Å^2^, meaning that they have good penetrability through cell membranes (Table 1) [46,67,68].

All compounds were predicted to have GI absorption and BBB penetration, which supports the ability of compounds **4a-o** to reach CNS and exert anticonvulsant effects (Table 2). These observations were also seen for the selected reference drugs. The likelihood of BBB permeation was further supported by the predicted logD values at pH = 7.4 and logBB values (Table 1). Compounds **4a-o** showed logD values between 3.52 and 5.83, indicating high lipophilicity, and all displayed logBB values greater than −1, which was considered by the developers of LogBB_Pred as compatible with BBB penetration (Table 1) [69]. These values are provided for guidance only. The model used (LogBB_Pred) was trained on datasets with limited representation of sulfur-containing heterocycles. Predicted positive LogBB values suggest likely penetration of the blood–brain barrier (BBB). Combined with the predicted TPSA values, all three parameters highlight the potential of compounds **4a-o** to penetrate the BBB.

Class homogeneity was observed in terms of interactions with P-gp and CYP450 isoenzymes. On one hand, no compound was predicted as P-gp substrates or as CYP2D6 inhibitors. On the other hand, all compounds were predicted as P-gp, CYP1A2, 2C9, 2C19, and 3A4 inhibitors (Table 2). This represents an important liability from a translational perspective, because inhibition of major drug-metabolizing CYP isoenzymes may increase the systemic exposure of co-administered drugs and thereby raise the risk of clinically relevant drug–drug interactions, unexpected adverse effects, and possible dose-adjustment requirements. CYP-mediated interactions are a well-recognized source of altered drug exposure, potentially resulting either in toxicity due to increased plasma concentrations or in reduced therapeutic efficacy when pharmacokinetics is perturbed. This issue may be particularly relevant in patients receiving polypharmacy, including psychotropic or anticonvulsant medications [70,71]. All compounds were predicted to have a short half-life, less than 3 h (Table 2).

Another important limitation of compounds **4a-o** is their predicted hepatotoxic potential, which was identified for all compounds. Additional toxicological liabilities included predicted carcinogenic risk for compounds **4a-e**, **4g-j**, **4m**, and **4o**, respiratory toxicity for compounds **4a**, **4i**, **4k**, **4l**, **4n**, and **4o**, and skin sensitization potential for compounds **4a**, **4b**, **4d-h**, **4j**, **4m**, and **4o** (Table 3). No compound was projected to cause eye irritation or rat toxicity in the applied prediction model. The hepatotoxicity signal deserves particular attention, since liver safety is a major consideration in the development of CNS-active compounds and anticonvulsant agents. Therefore, despite the promising anticonvulsant and BBB permeation profiles, the current series should still be regarded as consisting of preliminary leads, whose developability is weakened by predicted metabolic interaction risks and possible hepatic adverse effects [72].

### 3.3. Molecular Docking Studies

All compounds showed binding affinities on the two selected targets between −11.4 kcal/mol and −8.0 kcal/mol (Table 4). The affinity for the human α_1_β_2_γ_2_ GABA_A_ receptor was generally better compared to the affinity for the NR1 ligand-binding core of the NMDA receptor. In both cases, the affinity of the aromatic series (**4g-o**) to the targets was better compared to the aliphatic series (**4a-f**), thus highlighting the necessity of two phenyl rings for proper binding to both receptors [73].

The highest affinities for the GABA_A_ receptor were registered by compounds **4f** (ΔG = −11.4 kcal/mol), **4g** (ΔG = −11.0 kcal/mol), and **4i** (ΔG = −9.7 kcal/mol), while the highest affinities for the NMDA receptor were registered by compounds **4m** (ΔG = −9.8 kcal/mol), **4i** (ΔG = −9.7 kcal/mol), and **4j** (ΔG = −9.7 kcal/mol) (Table 4). Regarding the results on the GABA_A_ receptor, the presence of a cyclohexyl substituent (**4f**) and the existence of a second phenyl ring, either unsubstituted (**4g**) or substituted with small substituents (4-fluoro, **4i**), proved to be beneficial. Regarding the results on the NMDA receptor, halogenation of the compounds (**4i**, **4j**, **4m**, and **4o**) improved the affinity towards the target.

On the other hand, the lowest affinities to the GABA_A_ receptor were registered by compounds **4n** (ΔG = −8.6 kcal/mol) and **4h** (ΔG = −8.5 kcal/mol), while the lowest affinities to the NMDA receptor were registered by compounds **4b** (ΔG = −8.2 kcal/mol) and **4e** (ΔG = −8.1 kcal/mol) (Table 4). Regarding the results, the increasing steric volume of the compounds, as seen in **4h** (benzyl) and **4n** (3,4-dimethoxyphenyl), was not favorable for the affinity towards GABA_A_, while the alkyl substituents like ethyl (**4b**) and butyl (**4e**) affected the affinity for the NMDA receptor.

According to the graphical depictions of compounds **4c** and **4k** in the human α_1_β_2_γ_2_ GABA_A_ receptor (Figure 3), the sidechains of Ser205 and Tyr160 are predicted to act as HBD to the nitrogen and oxygen atoms from positions 1 and 4 of the quinazolin-4(3*H*)-one heterocycle in compound **4c**. This interaction is slightly different in compound **4k**, where the sidechain of Tyr160 acts as an HBD to the nitrogen atom from position 3 of the same heterocycle. Some π-π stacking interactions were observed between the phenyl ring of Phe77 and the quinazolin-4(3*H*)-one heterocycle of compound **4c** and between the phenyl ring of Tyr210 and the 4-methoxyphenyl substituent of compound **4k**. These interactions suggest that both the quinazolin-4(3*H*)-one heterocycle and the aromatic substituent contribute to receptor-site stabilization [26].

According to the graphical depiction of compound **4k** in the NR1 ligand-binding core of the NMDA receptor, the sidechain of Lys91 is predicted to act as an HBD to the oxygen atom in the methoxy group (Figure 4b). This interaction may contribute to and explain the favorable in vivo activity observed for compound **4k**.

### 3.4. Anticonvulsant Evaluation and Neuromotor Coordination Impairment

All tested compounds presented anticonvulsant activity to varying degrees, but were inferior to the reference drugs. The most notable activity was evidenced in compound **4k**, which protected all mice in the initial assay at 300 mg/kg, followed by compounds **4c**, **4a**, **4h**, and **4i** (Table 5). The difference between compounds **4c**, **4a**, **4h**, and **4i** was established based on the latency until the first seizure. Compound **4c** managed to maintain a prolonged protective effect, with a latency of 508.3 ± 266.1 s, in comparison to the other three compounds. The activity correlates to some extent with the results obtained in the molecular docking studies on the GABA_A_ and NMDA receptors (Table 4), especially for compounds **4c** (ΔG = −9.6 kcal/mol for GABA_A_ and ΔG = −8.6 kcal/mol for NMDA), **4a** (ΔG = −9.8 kcal/mol for GABA_A_ and ΔG = −8.9 kcal/mol for NMDA), and **4i** (ΔG = −10.9 kcal/mol for GABA_A_ and ΔG = −9.7 kcal/mol for NMDA). However, the other possible mechanisms that were not covered in this study could also be responsible for the anticonvulsant activity.

Some of the compounds, including **4b**, **4g**, and **4l,** have shown weak anticonvulsant activity with low protection (less than 50%) against PTZ-induced tonic–clonic lethal convulsions (Table 5). The weak activity of compound **4b** was reflected by the poor affinity of the compound for the NMDA receptor (ΔG = −8.2 kcal/mol, Table 4).

The in vivo activity showed only a partial agreement with the molecular docking results on the GABA_A_ and NMDA receptors. Therefore, the predicted ΔG values should not be interpreted as direct determinants of anticonvulsant efficacy, but rather as supportive in silico indicators of possible target engagement. Although molecular docking provides information about possible binding modes, the relationship between ΔG values and in vivo activity is not linear due to pharmacokinetic, metabolic, and tissue distribution factors.

The compounds **4c** and **4k**, which were considered the most potent compounds from both series, were further tested at four different increasing doses in order to establish their ED_50_ values. The selection of these compounds for extended evaluation was based on a comparison between the in silico descriptors related to CNS exposure (MLogP, logD, and logBB) and the in vivo results obtained in the PTZ-induced seizure model. Across the tested compounds, MLogP tended to have an inverse association with the mean number of seizures, suggesting that increased lipophilicity within an acceptable drug-like range may contribute to a reduction in the number of seizures. Correlations between logD or logBB and latency until first seizure (Ti) were weak, indicating that descriptors alone do not fully explain anticonvulsant efficacy. Therefore, their selection was supported by an integrated profile combining BBB permeation predictions, compliance of Lipinski’s rule of five, preserved neuromotor coordination, and promising in vivo anticonvulsant activity in the initial assay. Compound **4c** showed one of the longest latencies until first seizure, whereas compound **4k** achieved complete protection against PTZ-induced tonic–clonic lethal convulsions.

The Kaplan–Meier survival analysis showed that there are significant differences among the tested groups regarding the onset time to seizures induced by PTZ in mice (log-rank (Mantel–Cox) test: χ^2^(17) = 66.684, *p* < 0.001). Paired comparisons between the experimental groups and the negative control (NC) group were corrected using Bonferroni correction.

Among the tested compounds **4a-o**, compound **4c** had the highest median latency of seizure onset (202 s). However, the wide 95% confidence interval reflected considerable interindividual variability, and the difference compared to the NC group did not reach statistical significance (*p* = 0.135). Compounds **4g**, **4h**, **4i**, **4n** also showed a prolonged median latency of seizure onset (Ti) and a narrow confidence interval, indicating reduced biological variability.

Compounds **4e**, **4k**, and **4o** had median latencies longer than those of the NC, but the confidence interval was wide, suggesting marked biological variability. Compounds **4a**, **4d**, and **4l** showed seizure latency onset (Ti) similar to those of the NC group, and compounds **4b**, **4f**, **4j**, and **4m** did not differ significantly compared to the NC group (*p* = 1.000), suggesting the absence of a relevant anticonvulsant effect in the PTZ-induced seizure test.

Diazepam, used as a reference drug, induced complete protection against seizures (no seizure during the 1800 s observation period) and the results were statistically significant compared to the NC group (*p* = 0.009). Phenobarbital prolonged the seizure onset (Ti) with a median latency of 158 s [95%CI:59.6–256.4] compared to the NC group, but the difference was not statistically significant (*p* = 0.078).

Based on the literature data, by comparison to methaqualone (ED_50_ = 200.00 mg/kg) and clomethiazole (ED_50_ = 130.80 mg/kg), the starting points of this series, compound **4k** (ED_50_ = 84.313 mg/kg) was more potent compared to them, while compound **4c** (ED_50_ = 178.165 mg/kg) was only more potent than methaqualone [25,74,75,76,77]. The in vivo activity could not have been evaluated using clomethiazole due to its unavailability, nor with methaqualone since it is restricted and considered a high-risk substance of abuse in Romania [78].

According to the structure–activity relationships for anticonvulsant activity, the most favorable substitutions for the activity were *p*-methoxyphenyl (**4k**), allyl (**4c**), methyl (**4a**), benzyl (**4h**), and *p*-fluorophenyl (**4i**). The most unfavorable substitutions included ethyl (**4h**), phenyl (**4g**), and *m*-methoxyphenyl (**4l**). When comparing compounds **4k** and **4l**, the position of methoxy substitution becomes crucial for the activity. As observed in the molecular docking study, 4-methoxy substitution (**4k**) provided supplementary interactions between the compound and the studied targets (Figure 3 and Figure 4). The 3,4-dimethoxy-substituted compound **4m** had moderate anticonvulsant activity, situated between compounds **4k** and **4l**. Methoxy substitutions have long been proven to boost anticonvulsant activity [79,80,81,82]. Similarly, the allyl substitution has been considered advantageous for anticonvulsant activity due to its reduced rigidity and potential for deep penetration of the hydrophobic pockets of the GABA_A_ receptor [22,83].

None of the compounds **4a-o** produced neuromotor coordination impairment at the tested doses since none of the mice fell from the rotating rod (6 rpm) during the 180 s cutoff time period. Thus, compounds **4c** and **4k** have a reduced risk of neuromotor coordination impairment at their ED_50_. The protocol used in the study served as a screening test for neuromotor disorders.

### 3.5. Flumazenil Antagonism Assay

The absence of statistically significant differences between compounds **4c** and **4k** and diazepam in the flumazenil antagonism assay suggests that flumazenil may exert a similar blocking effect on all three tested substances. Under flumazenil pretreatment, none of them were able to protect the mice against PTZ-induced seizures. Therefore, this result highlights the potential mechanism of action of compounds **4c** and **4k** as PAMs of the GABA_A_ receptor. Nevertheless, it is important to mention that this assay represents a pharmacological model to evidence the effect of a potential PAM, and further studies are necessary to confirm this mechanism.

## 4. Materials and Methods

### 4.1. Chemistry

All the necessary laboratory glassware, porcelainware, reagents, and solvents used for the chemical synthesis, purification, and spectral analysis were purchased from the local suppliers.

The progress of the reactions and the purities of all compounds were verified by thin-layer chromatography (TLC), using silica gel 60 F_254_ as the stationary phase and ethyl acetate:heptane 7:1 mixture as the mobile phase.

The melting points (mp) were determined via thermal optical analysis using the MPM-H1 (Schropp Gerätetechnik, Überlingen, Germany) melting point device and the glass capillary method.

All intermediate compounds were confirmed using infrared (IR) and mass spectral (MS) analysis, while the final compounds were additionally confirmed by proton nuclear magnetic resonance (^1^H NMR) and carbon magnetic resonance (^13^C NMR) spectral analysis.

The IR spectral analysis was performed on an FT/IR 6100 spectrometer (Jasco, Cremella, Italy), and the samples were prepared in potassium bromide (KBr) tablets under vacuum.

The mass spectra (MS) for the intermediate compounds **3a-o** were recorded using positive ionization mode on an Agilent 1100 series device, connected to an Agilent Ion Trap SL mass spectrometer (Agilent Technologies, Santa Clara, CA, USA), while the samples were dissolved in a mixture of acetonitrile and dimethylsulfoxide (DMSO).

For the final compounds **4a-o**, chromatographic separation was carried out on a Shimadzu Nexera X3 HPLC system (Shimadzu Corporation, Kyoto, Japan) equipped with two LC-40D X3 pumps with degasser, a SIL-40C X3 autosampler, and a CTO-40S column oven. The LC system was coupled to a Shimadzu QTOF 9030 mass spectrometer. Sample preparation involved the use of a Sigma 204 Centrifuge (Osterode am Harz, Germany), an Analytical Plus Balance (Mettler-Toledo, Switzerland), and an Elma Transsonic 700/H ultrasonic bath (Singen, Germany). Chromatographic separation was achieved on a Phenomenex Gemini column (50 mm x 2.1 mm i.d., 3 µm particle size, Phenomenex, SUA) maintained at 40 °C. The mobile phase consisted of 0.1% (*v*/*v*) acetic acid in water (Solvent A) and acetonitrile (Solvent B). A gradient elution was applied as follows: 0–6 min, linear gradient from 10% to 90% B, followed by a re-equilibration step with 10% B for 2 min. The flow rate was set at 0.6 mL/min, and the injection volume was 1 µL. The identified retention time (RT) was mentioned as well. Mass spectrometry detection was performed in positive ionization mode using both MS (high-resolution scan mode—for accurate mass measurement of parent ions) and MS/MS (data-dependent analysis, for accurate mass measurement of daughter ions). The ESI source parameters were set as follows: source voltage 3000 V, interface temperature 350 °C, nebulizing gas flow 3 L/min, heating gas flow 12 L/min (air), drying gas flow 12 L/min (nitrogen), desolvation line temperature 250 °C, and heat block temperature 400 °C. The time-of-flight (TOF) tube was calibrated daily using a standard solution of sodium iodide of 400 mg/L. Data acquisition and data processing were made using LabSolutions 5.135 software (Shimadzu Corporation, Kyoto, Japan).

The NMR spectra were recorded using an Avance NMR spectrometer (Bruker, Karlsruhe, Germany). The samples were dissolved in DMSO-*d_6_*; tetramethylsilane (TMS) was used for calibration and the peak was used as a reference for the chemical shifts (ppm) calculation, which were reported in δ units. The identified signal multiplicity is presented using abbreviations for the peak patterns: app–apparent, br–broad, s–singlet, d–doublet, dd–double doublet, t–triplet, q–quartet, quin–quintet, sex–sextet, and m–multiplet. To identify an atom in a specific region of the molecule, the following abbreviations were used: Ar–phenyl, Th–thiazole, and Q–quinazolin-4(3*H*)-one.

#### 4.1.1. Synthesis of Intermediate 4-(Chloromethyl)-2-phenylthiazole (**1**)

The synthesis of this intermediate was previously reported by another research group [51]. The compound was also previously resynthesized by our research group as well [84].

The 4-(chloromethyl)-2-phenylthiazole intermediate (**1**) was obtained from the dissolution of 6 g (43.73 mmol) of thiobenzamide in 30 mL of anhydrous acetone, followed by the dissolution of 5.56 g (43.79 mmol) of 1,3-dichloracetone. The mixture was stirred for 24 h at room temperature, followed by vacuum filtration and washing with ethylic ether. The obtained precipitate was slowly poured over 15 mL of concentrated sulfuric acid, with constant stirring and cooling in an ice bath. The mixture was left to sit for 2 h and then poured over ice with constant stirring. The obtained product was filtered and abundantly washed with water until it was free of acid [84].

#### 4.1.2. Synthesis of Intermediate 3-Alkyl/aryl-2-mercaptoquinazolin-4(3*H*)-ones (**3a-o**)

In a round-bottom flask, 20 mmol of anthranilic acid was dissolved in 25 mL of absolute ethanol (EtOH), followed by the addition of 21 mmol of corresponding isothiocyanate (**2a-o**) and 30 mmol of triethylamine (Et_3_N). The isothiocyanates **2a-o** were purchased from local suppliers.

The obtained mixture was refluxed at 80 °C, using a heating mantle, for approximately one hour and 30 min, until an abundant precipitate was observed. The hot mixture was vacuum filtered and the crystalline precipitate was washed with absolute ethanol until it was free of anthranilic acid. This method was adapted from a previously reported method [85]. All intermediates were previously reported in the literature [52,53,54,55,56,57,58,59,60,61,62]. The decision to synthesize and characterize these intermediates was motivated by their lack of commercial availability and by the need to compare the obtained structural data with the literature data available for these compounds.

*2-Mercapto-3-methylquinazolin-4(3*H*)-one* (**3a**): white crystals; mp = 258–260 °C (lit. mp = 260–261 °C [52]); yield = 89.81%; FTIR (KBr) ν_max_ (cm^−1^): 3420.62 (str N-H), 1647.39 (C=O amide I), 1620.88 (C=O amide II), 1533.61 (C=N), 1489.26 (C-N), 772.83 (C-S); ESI^+^-MS: *m*/*z* 192.9 ([M+H]^+^).

*3-Ethyl-2-mercaptoquinazolin-4(3*H*)-one* (**3b**): white crystals; mp = 270 °C (lit. mp = 244–247 °C [55]); yield = 76.52%; FTIR (KBr) ν_max_ (cm^−1^): 3415.80 (str N-H), 1684.52 (C=O amide I), 1622.80 (C=O amide II), 1543.74 (C=N), 1489.74 (C-N), 762.23 (C-S); ESI^+^-MS: *m*/*z* 206.9 ([M+H]^+^).

*3-Allyl-2-mercaptoquinazolin-4(3*H*)-one* (**3c**): white–yellow crystals; mp = 205–206 °C (lit. mp = 206–207 °C [56]); yield = 73.76%; FTIR (KBr) ν_max_ (cm^−1^): 3417.73 (str N-H), 1655.11 (C=O amide I), 1622.80 (C=O amide II), 1530.24 (C=N), 1489.74 (C-N), 765.12 (C-S); ESI^+^-MS: *m*/*z* 219.0 ([M+H]^+^).

*3-Isopropyl-2-mercaptoquinazolin-4(3*H*)-one* (**3d**): yellow solid; mp = 173 °C (lit. mp = 177.5–178.5 °C [57]); yield = 19.93%; FTIR (KBr) ν_max_ (cm^−1^): 3420.62 (str N-H), 1697.53 (C=O amide I), 1623.29 (C=O amide II), 1544.22 (C=N), 1489.26 (C-N), 761.74 (C-S); ESI^+^-MS: *m*/*z* 220.9 ([M+H]^+^).

*3-Butyl-2-mercaptoquinazolin-4(3*H*)-one* (**3e**): white crystals; mp = 175 °C (lit. mp = 171–172 °C [55]); yield = 78.53%; FTIR (KBr) ν_max_ (cm^−1^): 3414.35 (str N-H), 1649.80 (C=O amide I), 1623.29 (C=O amide II), 1538.92 (C=N), 1489.74 (C-N), 757.89 (C-S); ESI^+^-MS: *m*/*z* 234.9 ([M+H]^+^).

*3-Cyclohexyl-2-mercaptoquinazolin-4(3*H*)-one* (**3f**): yellow crystals; mp = 269–270 °C (lit. mp = 270–271 °C [55]); yield = 11.78%; FTIR (KBr) ν_max_ (cm^−1^): 3420.62 (str N-H), 1688.85 (C=O amide I), 1624.25 (C=O amide II), 1544.22 (C=N), 1488.78 (C-N), 758.85 (C-S); ESI^+^-MS: *m*/*z* 261.0 ([M+H]^+^).

*2-Mercapto-3-phenylquinazolin-4(3*H*)-one* (**3g**): white crystals; mp > 280 °C (lit. mp > 300 °C [56]); yield = 81.91%; FTIR (KBr) ν_max_ (cm^−1^): 3414.83 (str N-H), 1661.86 (C=O amide I), 1623.29 (C=O amide II), 1544.22 (C=N), 1489.26 (C-N), 761.74 (C-S); ESI^+^-MS: *m*/*z* 255.0 ([M+H]^+^).

*3-Benzyl-2-mercaptoquinazolin-4(3*H*)-one* (**3h**): white crystals; mp = 254–255 °C (lit. mp = 246–247 °C [58]); yield = 85.18%; FTIR (KBr) ν_max_ (cm^−1^): 3418.21 (str N-H), 1667.89 (C=O amide I), 1621.84 (C=O amide II), 1541.33 (C=N), 1487.81 (C-N), 759.33 (C-S); ESI^+^-MS: *m*/*z* 269.0 ([M+H]^+^).

*3-(4-Fluorophenyl)-2-mercaptoquinazolin-4(3*H*)-one* (**3i**): white crystals; mp > 280 °C (lit. mp > 300 °C [59]); yield = 74.11%; FTIR (KBr) ν_max_ (cm^−1^): 3272.61 (str N-H), 1663.30 (C=O amide I), 1620.88 (C=O amide II), 1533.61 (C=N), 1486.85 (C-N), 761.26 (C-S); ESI^+^-MS: *m*/*z* 273.0 ([M+H]^+^).

*3-(4-Chlorophenyl)-2-mercaptoquinazolin-4(3*H*)-one* (**3j**): white crystals; mp > 280 °C (lit. mp = 253 °C [60]); yield = 88.85%; FTIR (KBr) ν_max_ (cm^−1^): 3246.57 (str N-H), 1661.86 (C=O amide I), 1618.95 (C=O amide II), 1532.17 (C=N), 1489.26 (C-N), 758.85 (C-S), 690.87 (C-Cl); ESI^+^-MS: *m*/*z* 288.9 ([M+H]^+^).

*2-Mercapto-3-(4-methoxyphenyl)-quinazolin-4(3*H*)-one* (**3k**): white crystals; mp > 280 °C (lit. mp = 284 °C [60]); yield = 85.04%; FTIR (KBr) ν_max_ (cm^−1^): 3240.79 (str N-H), 1661.37 (C=O amide I), 1619.91 (C=O amide II), 1532.17 (C=N), 1508.06 (C-N), 1248.68 (C-O), 760.30 (C-S); ESI^+^-MS: *m*/*z* 285.1 ([M+H]^+^).

*2-Mercapto-3-(3-methoxyphenyl)-quinazolin-4(3*H*)-one* (**3l**): white crystals; mp > 280 °C (lit. mp = 255–256 °C [61]); yield = 82.41%; FTIR (KBr) ν_max_ (cm^−1^): 3244.65 (str N-H), 1663.30 (C=O amide I), 1621.84 (C=O amide II), 1532.17 (C=N), 1489.26 (C-N), 1264.59 (C-O), 760.30 (C-S); ESI^+^-MS: *m*/*z* 285.0 ([M+H]^+^).

*2-Mercapto-3-(3-(trifluoromethyl)phenyl)-quinazolin-4(3*H*)-one* (**3m**): white crystals; mp = 268–269 °C (lit. mp = 265 °C [62]); yield = 60.22%; FTIR (KBr) ν_max_ (cm^−1^): 3420.62 (str N-H), 1660.41 (C=O amide I), 1622.32 (C=O amide II), 1533.13 (C=N), 1489.26 (C-N), 758.85 (C-S); ESI^+^-MS: *m*/*z* 323.1 ([M+H]^+^).

*3-(3,4-Dimethoxyphenyl)-2-mercaptoquinazolin-4(3*H*)-one* (**3n**): white crystals; mp > 280 °C (lit. mp = 245–247 °C [53]); yield = 90.23%; FTIR (KBr) ν_max_ (cm^−1^): 3245.13 (str N-H), 1663.78 (C=O amide I), 1619.91 (C=O amide II), 1531.12 (C=N), 1485.40 (C-N), 1266.04 (C-O), 757.41 (C-S); ESI^+^-MS: *m*/*z* 315.1 ([M+H]^+^).

*3-(3,4-Dichlorophenyl)-2-mercaptoquinazolin-4(3*H*)-one* (**3o**): white crystals; mp > 280 °C (no reported mp value in [54]); yield = 76.97%; FTIR (KBr) ν_max_ (cm^−1^): 3449.55 (str N-H), 1663.30 (C=O amide I), 1619.43 (C=O amide II), 1532.17 (C=N), 1488.78 (C-N), 760.30 (C-S), 694.73 (C-Cl); ESI^+^-MS: *m*/*z* 322.9 ([M+H]^+^).

#### 4.1.3. Synthesis of (2-Phenylthiazol-4-yl)methyl)thio)quinazolin-4(3*H*)-ones (**4a-o**)

In a round-bottom flask, 1 mmol of 3-alkyl/aryl-2-mercaptoquinazolin-4-(3*H*)-one (**3a-o**) was dissolved in 5 mL of *N*,*N*-dimethylformamide (DMF) and 5 mL of anhydrous acetone. Over the obtained solution, 2 mmol of potassium carbonate (K_2_CO_3_) was added and the mixture was stirred for 30 min at room temperature. Then, 1 mmol of intermediate **1** was added and the mixture was refluxed for 2 h, at 60 °C, using a heating mantle. The obtained mixture was poured over ice-cold water, while vigorously stirring, and the pH was adjusted to 7–8, with a few drops of 10% hydrochloric acid solution. The obtained suspension was vacuum filtered and the precipitate was washed with distilled water until it was free of potassium carbonate and potassium chloride. The obtained compounds were recrystallized from ethanol.

*3-Methyl-2-(((2-phenylthiazol-4-yl)methyl)thio)quinazolin-4(3*H*)-one* (**4a**): white solid; mp = 107 °C; yield = 60.83%; FTIR (KBr) ν_max_ (cm^−1^): 3420.62 (str N-H), 1680.18 (C=O amide I), 1607.38 (C=O amide II), 1550.01 (C=N), 1472.38 (C-N), 771.87 (C-S); ESI^+^-MS: *m*/*z* 366.0720 ([M+H]^+^), RT (min) = 5.189; ^1^H-NMR (DMSO-*d_6_*, 500 MHz) δ (ppm): 8.089–8.073 (d, 1H, Ar, *J* = 8 Hz), 7.932–7.916 (m, 2H, Ar), 7.818–7.798 (m, 2H, Ar), 7.690–7.673 (d, 1H, Ar, *J* = 8 Hz), 7.500–7.441 (m, 4H, Ar), 4.714 (s, 2H, -CH_2_-), 3.506 (s, 3H, -CH_3_); ^13^C-NMR (DMSO-*d_6_*, 125 MHz) δ (ppm): 167.030 (Th), 156.405 (C=O), 151.750 (Q), 146.760 (Th), 135.309 (Ar), 134.546 (Ar), 132.796 (Ar), 130.319 (Ar), 129.241 (Ar), 127.232 (Ar), 126.385 (Ar), 126.056 (Ar), 118.623 (Ar), 118.378 (Th), 115.522 (Q), 31.426 (-CH_2_-), 30.019 (-CH_3_).

*3-Ethyl-2-(((2-phenylthiazol-4-yl)methyl)thio)quinazolin-4(3*H*)-one* (**4b**): white solid; mp = 117–118 °C; yield = 82.44%; FTIR (KBr) ν_max_ (cm^−1^): 3421.10 (str N-H), 1670.53 (C=O amide I), 1606.41 (C=O amide II), 1550.01 (C=N), 1507.58 (C-N), 772.35 (C-S); ESI^+^-MS: *m*/*z* 380.0879 ([M+H]^+^), RT (min) = 5.381; ^1^H-NMR (DMSO-*d_6_*, 500 MHz) δ (ppm): 8.090–8.075 (d, 1H, Ar, *J* = 7.5 Hz), 7.933–7.917 (m, 2H, Ar), 7.824–7.794 (m, 2H, Ar), 7.684–7.668 (d, 1H, Ar, *J* = 8 Hz), 7.503–7.447 (m, 4H, Ar), 4.722 (s, 2H, -CH_2_-), 4.118–4.975 (q, 2H, -CH_2_-, *J* = 7 Hz), 1.278–1.249 (t, 3H, -CH_3_, *J* = 7 Hz); ^13^C-NMR (DMSO-*d_6_*, 125 MHz) δ (ppm): 167.037 (Th), 160.304 (C=O), 155.516 (Q), 151.750 (Q), 146.760 (Th), 134.630 (Ar), 132.803 (Ar), 130.319 (Ar), 129.241 (Ar), 126.329 (Ar), 126.056 (Ar), 118.861 (Ar), 118.371 (Th), 115.200 (Q), 33.456 (-CH_2_-), 31.426 (-CH_2_-), 13.018 (-CH_3_).

*3-Allyl-2-(((2-phenylthiazol-4-yl)methyl)thio)quinazolin-4(3*H*)-one* (**4c**): white solid; mp = 109 °C; yield = 71.50%; FTIR (KBr) ν_max_ (cm^−1^): 3420.60 (str N-H), 1671.02 (C=O amide I), 1604.97 (C=O amide II), 1543.74 (C=N), 1507.58 (C-N), 774.28 (C-S); ESI^+^-MS: *m*/*z* 392.0871 ([M+H]^+^), RT (min) = 5.369; ^1^H-NMR (DMSO-*d_6_*, 500 MHz) δ (ppm): 8.095–8.080 (d, 1H, Ar, *J* = 7.5 Hz), 7.922–7.907 (m, 2H, Ar), 7.836–7.807 (m, 1H, Ar), 7.771 (s, 1H, Th), 7.699–7.682 (d, 1H, Ar, *J* = 8.5 Hz), 7.497–7.458 (m, 4H, Ar), 5.947–5.871 (m, 1H, -CH=), 5.193–5.092 (m, 2H, =CH_2_), 4.706 (br s, 4H, SCH_2_ + NCH_2_, overlapped); ^13^C-NMR (DMSO-*d_6_*, 125 MHz) δ (ppm): 166.575 (Th), 160.339 (C=O), 155.929 (Q), 151.729 (Q), 146.767 (Th), 134.777 (Ar), 132.796 (Ar), 131.424 (Ar), 130.319 (Ar), 129.234 (Ar), 127.260 (Ar), 126.455 (Ar), 126.049 (Ar), 124.481 (-CH=), 118.742 (Ar), 118.364 (Th), 117.370 (=CH_2_), 45.704 (-CH_2_-), 31.538 (-CH_2_-).

*3-Isopropyl-2-(((2-phenylthiazol-4-yl)methyl)thio)quinazolin-4(3*H*)-one* (**4d**): white solid; mp = 158 °C; yield = 76.27%; FTIR (KBr) ν_max_ (cm^−1^): 3420.62 (str N-H), 1676.32 (C=O amide I), 1606.90 (C=O amide II), 1548.56 (C=N), 1473.35 (C-N), 769.46 (C-S); ESI^+^-MS: *m*/*z* 394.1024 ([M+H]^+^), RT (min) = 5.748; ^1^H-NMR (DMSO-*d_6_*, 500 MHz) δ (ppm): 8.056–8.040 (d, 1H, Ar, *J* = 8 Hz), 7.936–7.921 (m, 2H, Ar), 7.799–7.774 (m, 2H, Ar), 7.659–7.643 (d, 1H, Ar, *J* = 8 Hz), 7.506–7.494 (m, 3H, Ar), 7.458–7.428 (t, 1H, Ar, *J* = 7.5 Hz), 4.690 (br s, 3H, SCH_2_ + NCH, overlapped), 1.570–1.557 (d, 6H, -CH_3_, *J* = 6.5 Hz); ^13^C-NMR (DMSO-*d_6_*, 125 MHz) δ (ppm): 167.030 (Th), 160.975 (C=O), 155.775 (Q), 151.673 (Q), 146.242 (Th), 134.518 (Ar), 132.803 (Ar), 130.326 (Ar), 129.248 (Ar), 126.063 (Ar), 125.895 (Ar), 125.727 (Ar), 119.953 (Ar), 118.427 (Th), 42.247 (CH), 32.175 (-CH_2_-), 19.149 (-CH_3_).

*3-Butyl-2-(((2-phenylthiazol-4-yl)methyl)thio)quinazolin-4(3*H*)-one* (**4e**): white solid; mp = 98–99 °C; yield = 88.91%; FTIR (KBr) ν_max_ (cm^−1^): 3413.39 (str N-H), 1662.20 (C=O amide I), 1617.98 (C=O amide II), 1544.22 (C=N), 1468.53 (C-N), 774.76 (C-S); ESI^+^-MS: *m*/*z* 408.1187 ([M+H]^+^), RT (min) = 5.932; ^1^H-NMR (DMSO-*d_6_*, 500 MHz) δ (ppm): 8.081–8.065 (d, 1H, Ar, *J* = 8 Hz), 7.924–7.908 (m, 2H, Ar), 7.817–7.777 (m, 2H, Ar), 7.678–7.662 (d, 1H, Ar, *J* = 8 Hz), 7.495–7.441 (m, 4H, Ar), 4.714 (s, 2H, -CH_2_-), 4.049–4.018 (t, 2H, -CH_2_-, *J* = 8 Hz), 1.678–1.617 (quin, 2H, -CH_2_-, *J* = 7.5 Hz), 1.381–1.307 (sex, 2H, -CH_2_-, *J* = 7.5 Hz), 0.910–0.881 (t, 3H, -CH_3_, *J* = 7.5 Hz); ^13^C-NMR (DMSO-*d_6_*, 125 MHz) δ (ppm): 167.016 (Th), 160.486 (C=O), 155.691 (Q), 151.778 (Q), 146.718 (Th), 134.637 (Ar), 132.803 (Ar), 130.319 (Ar), 129.234 (Ar), 126.371 (Ar), 126.042 (Ar), 126.000 (Ar), 118.798 (Ar), 118.385 (Th), 43.752 (-CH_2_-), 31.510 (-CH_2_-), 29.585 (-CH_2_-), 19.562 (-CH_2_-), 13.522 (-CH_3_).

*3-Cyclohexyl-2-(((2-phenylthiazol-4-yl)methyl)thio)quinazolin-4(3*H*)-one* (**4f**): white solid; mp = 116 °C; yield = 87.50%; FTIR (KBr) ν_max_ (cm^−1^): 3447.13 (str N-H), 1681.14 (C=O amide I), 1606.41 (C=O amide II), 1550.01 (C=N), 1472.38 (C-N), 773.32 (C-S); ESI^+^-MS: *m*/*z* 434.1347 ([M+H]^+^), RT (min) = 6.145; ^1^H-NMR (DMSO-*d_6_*, 500 MHz) δ (ppm): 8.047–8.031 (d, 1H, Ar, *J* = 8 Hz), 7.935–7.919 (m, 2H, Ar), 7.803–7.773 (m, 2H, Ar), 7.657–7.641 (d, 1H, Ar, *J* = 8 Hz), 7.505–7.492 (m, 3H, Ar), 7.457–7.426 (t, 1H, Ar, *J* = 8 Hz), 4.687 (s, 2H, -CH_2_-), 1.830–1.626 (m, 6H, -C_6_H_11_), 1.364–1.153 (m, 5H, -C_6_H_11_); ^13^C-NMR (DMSO-*d_6_*, 125 MHz) δ (ppm): 167.065 (Th), 161.045 (C=O), 157.154 (Q), 151.582 (Q), 146.158 (Th), 134.567 (Ar), 132.803 (Ar), 130.340 (Ar), 129.255 (Ar), 126.077 (Ar), 125.951 (Ar), 125.727 (Ar), 118.588 (Th), 50.380 (CH), 32.287 (-CH_2_-), 28.129 (-CH_2_-), 27.534 (-CH_2_-), 26.001 (-CH_2_-), 25.728 (-CH_2_-), 24.798 (-CH_2_-).

*3-Phenyl-2-(((2-phenylthiazol-4-yl)methyl)thio)quinazolin-4(3*H*)-one* (**4g**): white solid; mp = 196–197 °C; yield = 88.99%; FTIR (KBr) ν_max_ (cm^−1^): 3420.62 (str N-H), 1689.82 (C=O amide I), 1606.41 (C=O amide II), 1549.04 (C=N), 1473.35 (C-N), 773.32 (C-S); ESI^+^-MS: *m*/*z* 428.0876 ([M+H]^+^), RT (min) = 5.139; ^1^H-NMR (DMSO-*d_6_*, 500 MHz) δ (ppm): 8.101–8.085 (d, 1H, Ar, *J* = 8 Hz), 7.883–7.844 (m, 3H, Ar), 7.750–7.739 (m, 2H, Ar), 7.557–7.545 (m, 3H, Ar), 7.508–7.468 (m, 6H, Ar), 4.573 (s, 2H, -CH_2_-); ^13^C-NMR (DMSO-*d_6_*, 125 MHz) δ (ppm): 166.981 (Th), 160.702 (C=O), 156.594 (Q), 151.694 (Q), 147.194 (Th), 135.729 (Ar), 134.896 (Ar), 134.392 (Ar), 132.754 (Ar), 130.291 (Ar), 130.892 (Ar), 129.465 (Ar), 129.423 (Ar), 129.220 (Ar), 127.546 (Ar), 126.168 (Ar), 126.028 (Ar), 118.273 (Th), 31.951 (-CH_2_-).

*3-Benzyl-2-(((2-phenylthiazol-4-yl)methyl)thio)quinazolin-4(3*H*)-one* (**4h**): white solid; mp = 140 °C; yield = 85.74%; FTIR (KBr) ν_max_ (cm^−1^): 3446.65 (str N-H), 1680.18 (C=O amide I), 1622.93 (C=O amide II), 1538.92 (C=N), 1469.01 (C-N), 777.17 (C-S); ESI^+^-MS: *m*/*z* 442.1028 ([M+H]^+^), RT (min) = 5.469; ^1^H-NMR (DMSO-*d_6_*, 500 MHz) δ (ppm): 8.128–8.112 (d, 1H, Ar, *J* = 8 Hz), 7.894–7.829 (m, 3H, Ar), 7.730–7.709 (m, 2H, Ar), 7.510–7.479 (m, 4H, Ar), 7.307–7.230 (m, 5H, Ar), 5.327 (s, 2H, -CH_2_-), 4.687 (s, 2H, -CH_2_-); ^13^C-NMR (DMSO-*d_6_*, 125 MHz) δ (ppm): 167.009 (Th), 160.898 (C=O), 156.146 (Q), 151.673 (Q), 146.802 (Th), 135.617 (Ar), 134.938 (Ar), 132.775 (Ar), 130.319 (Ar), 129.227 (Ar), 128.541 (Ar), 128.058 (Ar), 127.358 (Ar), 127.358 (Ar), 127.106 (Ar), 126.679 (Ar), 126.595 (Ar), 126.049 (Ar), 118.364 (Th), 46.747 (-CH_2_-), 31.692 (-CH_2_-).

*3-(4-Fluorophenyl)-2-(((2-phenylthiazol-4-yl)methyl)thio)quinazolin-4(3*H*)-one* (**4i**): white solid; mp = 172–173 °C; yield = 75.85%; FTIR (KBr) ν_max_ (cm^−1^): 3447.54 (str N-H), 1672.46 (C=O amide I), 1607.86 (C=O amide II), 1544.70 (C=N), 1507.10 (C-N), 773.80 (C-S); ESI^+^-MS: *m*/*z* 446.0780 ([M+H]^+^), RT (min) = 5.209; ^1^H-NMR (DMSO-*d_6_*, 500 MHz) δ (ppm): 8.097–8.082 (d, 1H, Ar, *J* = 7.5 Hz), 7.890–7.846 (m, 3H, Ar), 7.750–7.733 (m, 2H, Ar), 7.579–7.552 (m, 2H, Ar), 7.510–7.475 (m, 4H, Ar), 7.409–7.375 (app t, 2H, Ar, *J* ≈ 8.5 Hz), 4.580 (s, 2H, -CH_2_-); ^13^C-NMR (DMSO-*d_6_*, 125 MHz) δ (ppm): 163.523 (Th), 161.556 (Q), 160.786 (C=O), 156.573 (Q), 151.659 (Q), 147.180 (Th), 134.917 (Ar), 132.754 (Ar), 131.977 (Ar), 131.886 (Ar), 131.816 (Ar), 130.298 (Ar), 129.220 (Ar), 126.546 (Ar), 126.168 (Ar), 126.035 (Ar), 119.603 (Ar), 118.308 (Th), 116.523 (Ar), 116.334 (Ar), 31.972 (-CH_2_-).

*3-(4-Chlorophenyl)-2-(((2-phenylthiazol-4-yl)methyl)thio)quinazolin-4(3*H*)-one* (**4j**): white solid; mp = 183 °C; yield = 83.04%; FTIR (KBr) ν_max_ (cm^−1^): 3447.13 (str N-H), 1687.41 (C=O amide I), 1606.41 (C=O amide II), 1550.01 (C=N), 1508.06 (C-N), 775.24 (C-S), 689.43 (C-Cl); ESI^+^-MS: *m*/*z* 462.0485 ([M+H]^+^), RT (min) = 5.444; ^1^H-NMR (DMSO-*d_6_*, 500 MHz) δ (ppm): 8.098–8.083 (d, 1H, Ar, *J* = 7.5 Hz), 7.892–7.850 (m, 3H, Ar), 7.754–7.736 (m, 2H, Ar), 7.638–7.622 (m, 2H, Ar), 7.556–7.539 (m, 2H, Ar), 7.514–7.477 (m, 4H, Ar), 4.587 (s, 2H, -CH_2_-); ^13^C-NMR (DMSO-*d_6_*, 125 MHz) δ (ppm): 166.400 (Th), 160.681 (C=O), 156.202 (Q), 151.617 (Q), 147.173 (Th), 135.624 (Ar), 134.959 (Ar), 134.644 (Ar), 134.035 (Ar), 132.761 (Ar), 131.466 (Ar), 131.026 (Ar), 130.305 (Ar), 129.584 (Ar), 129.227 (Ar), 128.961 (Ar), 126.553 (Ar), 126.189 (Ar), 126.042 (Ar), 124.369 (Ar), 119.589 (Ar), 118.343 (Th), 31.972 (-CH_2_-).

*3-(4-Methoxyphenyl)-2-(((2-phenylthiazol-4-yl)methyl)thio)quinazolin-4(3*H*)-one* (**4k**): white solid; mp = 188 °C; yield = 85.74%; FTIR (KBr) ν_max_ (cm^−1^): 3447.13 (str N-H), 1693.19 (C=O amide I), 1606.41 (C=O amide II), 1548.56 (C=N), 1471.42 (C-N), 1265.56 (C-O), 777.65 (C-S); ESI^+^-MS: *m*/*z* 458.0979 ([M+H]^+^), RT (min) = 5.131; ^1^H-NMR (DMSO-*d_6_*, 500 MHz) δ (ppm): 8.091–8.076 (d, 1H, Ar, *J* = 7.5 Hz), 7.887–7.829 (m, 3H, Ar), 7.733–7.716 (m, 2H, Ar), 7.494–7.451 (m, 4H, Ar), 7.377–7.359 (m, 2H, Ar), 7.077–7.060 (d, 2H, Ar, *J* = 8.5 Hz), 4.556 (s, 2H, -CH_2_-), 3.816 (s, 3H, -CH_3_); ^13^C-NMR (DMSO-*d_6_*, 125 MHz) δ (ppm): 162.956 (Th), 160.884 (Q), 160.038 (C=O), 157.301 (Q), 151.722 (Q), 147.215 (Th), 134.819 (Ar), 132.754 (Ar), 130.606 (Ar), 130.291 (Ar), 129.920 (Ar), 129.220 (Ar), 128.086 (Ar), 126.560 (Ar), 126.133 (Ar), 126.035 (Ar), 125.937 (Ar), 119.596 (Ar), 118.245 (Th), 114.598 (Ar), 55.405 (-CH_3_), 32.014 (-CH_2_-).

*3-(3-Methoxyphenyl)-2-(((2-phenylthiazol-4-yl)methyl)thio)quinazolin-4(3*H*)-one* (**4l**): white solid; mp = 150–151 °C; yield = 65.41%; FTIR (KBr) ν_max_ (cm^−1^): 3420.14 (str N-H), 1687.41 (C=O amide I), 1604.48 (C=O amide II), 1545.18 (C=N), 1489.26 (C-N), 1270.38 (C-O), 767.05 (C-S); ESI^+^-MS: *m*/*z* 458.0973 ([M+H]^+^), RT (min) = 5.154; ^1^H-NMR (DMSO-*d_6_*, 500 MHz) δ (ppm): 8.097–8.082 (d, 1H, Ar, *J* = 7.5 Hz), 7.882–7.838 (m, 3H, Ar), 7.743–7.723 (m, 2H, Ar), 7.503–7.441 (m, 5H, Ar), 7.117–7.103 (m, 2H, Ar), 7.034–7.019 (d, 1H, Ar, *J* = 7.5 Hz), 4.572 (s, 2H, -CH_2_-), 3.769 (s, 3H, -CH_3_); ^13^C-NMR (DMSO-*d_6_*, 125 MHz) δ (ppm): 166.988 (Th), 160.597 (Q), 159.926 (C=O), 156.573 (Q), 151.694 (Q), 147.166 (Th), 136.772 (Ar), 134.868 (Ar), 132.754 (Ar), 130.291 (Ar), 130.165 (Ar), 129.220 (Ar), 126.532 (Ar), 126.133 (Ar), 126.035 (Ar), 121.423 (Ar), 119.617 (Ar), 118.273 (Th), 115.424 (Ar), 115.186 (Ar), 55.440 (-CH_3_), 31.944 (-CH_2_-).

*2-(((2-Phenylthiazol-4-yl)methyl)thio)-3-(3-(trifluoromethyl)phenyl)quinazolin-4(3*H*)-one* (**4m**): white solid; mp = 172–173 °C; yield = 75.85%; FTIR (KBr) ν_max_ (cm^−1^): 3421.10 (str N-H), 1680.18 (C=O amide I), 1607.86 (C=O amide II), 1550.01 (C=N), 1472.38 (C-N), 774.76 (C-S); ESI^+^-MS: *m*/*z* 496.0752 ([M+H]^+^), RT (min) = 5.470; ^1^H-NMR (DMSO-*d_6_*, 500 MHz) δ (ppm): 8.102–8.087 (d, 1H, Ar, *J* = 7.5 Hz), 8.044 (s, 1H, Ar), 7.940–7.924 (d, 1H, Ar, *J* = 8 Hz), 7.884–7.854 (m, 4H, Ar), 7.820–7.788 (m, 1H, Ar), 7.763–7.748 (m, 2H, Ar), 7.520–7.465 (m, 4H, Ar), 4.647–4.558 (m, 2H, -CH_2_-); ^13^C-NMR (DMSO-*d_6_*, 125 MHz) δ (ppm): 164.160 (Th), 160.786 (C=O), 155.845 (Q), 151.575 (Q), 147.180 (Th), 136.632 (Ar), 135.001 (Ar), 133.993 (Ar), 132.754 (Ar), 130.760 (Ar), 130.305 (Ar), 129.227 (Ar), 126.784 (Ar), 126.539 (Ar), 126.217 (Ar), 126.112 (Ar), 126.028 (Ar), 119.659 (Ar), 118.413 (Th), 31.993 (-CH_2_-).

*3-(3,4-Dimethoxyphenyl)-2-(((2-phenylthiazol-4-yl)methyl)thio)quinazolin-4(3*H*)-one* (**4n**): white solid; mp = 184 °C; yield = 88.98%; FTIR (KBr) ν_max_ (cm^−1^): 3440.39 (str N-H), 1671.50 (C=O amide I), 1602.56 (C=O amide II), 1545.18 (C=N), 1472.38 (C-N), 1264.11 (C-O), 769.46 (C-S); ESI^+^-MS: *m*/*z* 488.1083 ([M+H]^+^), RT (min) = 4.891; ^1^H-NMR (DMSO-*d_6_*, 500 MHz) δ (ppm): 8.096–8.080 (d, 1H, Ar, *J* = 8 Hz), 7.883–7.829 (m, 3H, Ar), 7.740–7.715 (m, 2H, Ar), 7.496–7.468 (m, 4H, Ar), 7.105–7.064 (m, 2H, Ar), 7.000–6.981 (m, 1H, Ar), 4.600–4.513 (m, 2H, -CH_2_-), 3.819 (s, 3H, -CH_3_), 3.718 (s, 3H, -CH_3_); ^13^C-NMR (DMSO-*d_6_*, 125 MHz) δ (ppm): 166.267 (Th), 159.996 (C=O), 157.273 (Q), 151.757 (Q), 149.112 (Q), 147.208 (Th), 138.452 (Ar), 134.812 (Ar), 132.775 (Ar), 130.298 (Ar), 129.227 (Ar), 128.156 (Ar), 126.546 (Ar), 126.112 (Ar), 126.035 (Ar), 125.930 (Ar), 123.669 (Ar), 121.766 (Ar), 119.631 (Ar), 118.238 (Th), 112.841 (Ar), 111.540 (Ar), 55.790 (-CH_3_), 55.615 (-CH_3_), 32.035 (-CH_2_-).

*3-(3,4-Dichlorophenyl)-2-(((2-phenylthiazol-4-yl)methyl)thio)quinazolin-4(3*H*)-one* (**4o**): white solid; mp = 177–178 °C; yield = 52.21%; FTIR (KBr) ν_max_ (cm^−1^): 3450.51 (str N-H), 1663.55 (C=O amide I), 1607.38 (C=O amide II), 1544.22 (C=N), 1467.80 (C-N), 762.23 (C-S), 696.66 (C-Cl); ESI^+^-MS: *m*/*z* 496.0096 ([M+H]^+^), RT (min) = 5.734; ^1^H-NMR (DMSO-*d_6_*, 500 MHz) δ (ppm): 8.098–8.092 (d, 1H, Ar, *J* = 8 Hz), 7.986–7.982 (m, 1H, Ar), 7.896–7.842 (m, 4H, Ar), 7.769–7.740 (m, 2H, Ar), 7.594–7.572 (m, 1H, Ar), 7.517–7.475 (m, 4H, Ar), 4.636–4.573 (m, 2H, -CH_2_-); ^13^C-NMR (DMSO-*d_6_*, 125 MHz) δ (ppm): 167.037 (Th), 160.625 (C=O), 155.726 (Q), 151.519 (Q), 147.117 (Th), 135.687 (Ar), 135.043 (Ar), 132.950 (Ar), 132.747 (Ar), 131.767 (Ar), 131.704 (Ar), 131.397 (Ar), 130.305 (Ar), 130.179 (Ar), 129.220 (Ar), 126.546 (Ar), 126.210 (Ar), 126.147 (Ar), 126.042 (Ar), 119.568 (Ar), 118.441 (Th), 31.993 (-CH_2_-).

### 4.2. Computational Studies

#### 4.2.1. Druggability and ADMETox Prediction Studies

The SwissADME web tool was employed for the in silico prediction of the druggability of compounds **4a-o** [67]. For comparison reasons, phenobarbital (**PHE**), diazepam (**DZP**), clomethiazole (**CMT**), methaqualone (**MTQ**), and pentylenetetrazole (**PTZ**) were also included in this prediction.

The prediction of logBB was performed using the LogBB_Pred online application. This parameter represents a logarithmic ratio between the concentration of a drug in the brain and the concentration of the same drug in the blood [69].

The in silico ADMETox and logD at pH = 7.4 predictions were performed using the Deep-PK online platform [86]. LogD is a parameter that measures the distribution of a compound between a lipid phase and an aqueous phase [87]. The chemical structures of compounds **4a-o**, along with **PHE**, **DZP**, **MTQ**, **CMT**, and **PTZ,** were prepared as optimized 3D structures (.sdf file extension) and loaded into the platform.

#### 4.2.2. Molecular Docking Studies

The molecular docking of compounds **4a-o** to the human GABA_A_ receptor α_1_β_2_γ_2_ subtype and the NR1 ligand-binding core of the NMDA receptor was carried out using AutoDock Vina 1.1.2 [88]. The required input files were generated using AutoDock Tools 1.5.6 and OpenBabel 2.3.2, following previously reported methodologies, by applying the Gasteiger charges and the addition of the polar hydrogen atoms [89,90,91]. The target protein structures were taken from the RCSB Protein Data Bank (entry codes: 6X3Z and 1PBQ), and unnecessary co-crystallized molecule removal, polar hydrogen addition, charge calculation, and proper protonation were carried out [90,92,93,94]. The search space was configured as a cube, with side sizes set to x = y = z = 20 in both cases. The center coordinates of the cube search space were set to x = 81.211, y = 120.374, z = 119.414 for the human α_1_β_2_γ_2_ GABA_A_ receptor and x = 2.462, y = 39.384, z = -17.719 for the NR1 ligand-binding core of the NMDA receptor. The conformation with the best binding energy for each compound, expressed as a variation in Gibbs free energy (ΔG), was searched in these zones. The visualization was made using Chimera 1.10.2 [95].

### 4.3. Biological Evaluation

#### 4.3.1. Animals, Substances, and Ethics

This study has been authorized by notice no. AVZ267/19.10.2023 of the Ethics Commission of the “Iuliu Hațieganu” University of Medicine and Pharmacy Cluj-Napoca and by project authorization no. 386/14.11.2023 of the Cluj Sanitary Veterinary and Food Safety Directorate of the National Sanitary Veterinary and Food Safety Authority. The drugs used in this study were acquired from local suppliers and produced by Terapia S.A. (Cluj-Napoca, Romania), Hermes Arzneimittel (Pullach im Isartal, Germany), and Sigma-Aldrich (Burlington, MA, USA).

The animal species selected for this study was the laboratory mouse (*Mus musculus* CD1), and female specimens aged between 6 and 8 weeks and weighting between 20 and 30 g were used. All animals were requested from the Biobase—Center for Experimental Medicine and Practical Skills of the “Iuliu Hațieganu” University of Medicine and Pharmacy Cluj-Napoca.

Each group included in the study was placed separately in type-IV polycarbonate cages, provided with a grill in the superior part. Sufficient wood chips were placed in each cage, necessary for the animals to build shelters. Environmental enrichment was realized by placing paper pieces, plastic, and carton tubes to stimulate the natural behaviors of mice and increase their well-being [96].

The animals were kept in optimal conditions, with constant temperature (22 ± 2 °C), stable air humidity (45 ± 10%), a day–night cycle of 12/12 h, and ensuring that light intensity did not surpass 300 lux during daytime. The animals had ad libitum access to standard food and water [96].

The mice were weighed and divided into 18 groups (*n* = 6 mice for each group): 1 negative control group, 2 positive control groups, and 15 groups for testing compounds **4a-o**. Eight more groups were used for further testing of compounds **4c** and **4k**.

The substances were administered intraperitoneally (i.p.) as dispersions in a vehicle composed of saline solution, 0.1% Tween 80 *v*/*v*, and 2% propylene glycol *v*/*v*. Compounds **4a-o** were administered in a single dose (D = 300 mg/kg) for the initial screening. Compounds **4c** and **4k** were later administered in four different doses (D_1_ = 50 mg/kg, D_2_ = 150 mg/kg, D_3_ = 300 mg/kg, and D_4_ = 450 mg/kg) in order to determine ED_50_. The negative control group received only the vehicle, while the positive control groups were administered diazepam at 2 mg/kg i.p., and phenobarbital at 15 mg/kg i.p., respectively, dissolved in the same vehicle. The reference drugs were selected due to their activity as PAMs on the GABA_A_ receptor [22]. The doses were established according to previous reports in the literature [29,97]. The initial 300 mg/kg dose was selected as an exploratory screening dose based on the literature precedent for structurally related quinazolin-4(3*H*)-one anticonvulsants and related sulfur-containing heterocyclic anticonvulsant compounds, where screening doses up to 300 mg/kg have been reported [32,98,99].

During the restriction procedures, the animals were taken out of the cage using a carton tube and were placed on a solid support to ensure they had grip. Then, the restriction maneuver was performed by grabbing the animal from the lax skin of the cervical region and fixating the basal region of the tail to ensure the proper intraperitoneal administration of the compounds [96,100].

At the end of the study, the animals were euthanized by inducing a rapid and pain-free death. Animal euthanasia was ensured by overdosing a xylazine:ketamine 1:2 (2 mL:4 mL) anesthetic mixture to induce hypersedation and analgesia, followed by cervical dislocation. All ethical principles in scientific research were respected in order to avoid the unnecessary suffering of the animals used [96,100,101].

#### 4.3.2. Rotarod Test (RR)

Before the administration of any substance, the mice were trained on the Rotarod (Mouse RotaRod, Ugo Basile, Gemonio VA, Italy) for 180 s, at a speed of 6 rotations/minute (rpm) [102,103]. The training process was done three times for each mouse.

Following the initial Rotarod test, the substances were i.p. administered. After the administration, the mice were left for 30 min, then were subjected again to the Rotarod test for another 180 s at 6 rpm [102]. The mice were considered to have impaired neuromotor coordination if they fell off the rotating cylinder during this time. The results were quantified as a percentage (%) of preserved neuromotor coordination (B/T*100, where B is the number of mice that maintained their balance and T is the number of mice tested in a group). The Rotarod performance test was done three times for each mouse.

#### 4.3.3. Pentylenetetrazole (PTZ)-Induced Seizure Model

Following the Rotarod test, seizures were induced by i.p. administration of 75 mg/kg of PTZ and the behavior was monitored for 30 min by counting the number of tonic–clonic seizures. The results were quantified as the latency until first seizure (Ti)*,* number of seizures, and protection against tonic–clonic lethal seizures (PTZ P/T, where P is the number of mice protected and T is the number of mice tested). The last parameter was calculated as a ratio between the number of mice protected against tonic–clonic lethal seizures and the number of tested mice in a group (*n* = 6) and expressed as a percentage (%) [22,29,104]. An animal was considered protected if the tonic–clonic lethal seizures did not occur during the 30 min observation time [105].

Initially, all compounds were tested at a dose of 300 mg/kg. The most promising compounds, **4c** and **4k**, were further tested in four different doses, according to a previously reported study, in order to determine ED_50_ [35].

#### 4.3.4. Flumazenil Antagonism Assay

The flumazenil antagonism assay was employed in order to establish whether the anticonvulsant activity of the tested compounds is mediated via the diazepam binding site of the GABA_A_ receptor. The assay was performed for compounds **4c** and **4k**, which emerged as the best anticonvulsants from the tested compounds, based on an adaptation of the previously reported method by our research group [22].

The assay consisted of two parts. The first part was necessary to establish the optimal dose of flumazenil that inhibited 50% to 100% of diazepam activity and the second part was necessary to establish if the anticonvulsant activity of compounds **4c** and **4k** is due to their interaction with the diazepam binding site of the GABA_A_ receptor.

For the first part of the assay, 18 mice were weighed and divided into 3 groups (*n* = 6 mice for each group), and each group received three different increasing doses of flumazenil i.p. (5 mg/kg for group 1, 7 mg/kg for group 2, and 10 mg/kg for group 3), followed by 2 mg/kg of diazepam i.p. 5 min later. After an incubation period of 30 min, the mice received 75 mg/kg of PTZ i.p. The mice were monitored for 30 min, following the same protocol from the PTZ-induced seizure model. Based on the observations, the optimal dose of flumazenil was established at 5 mg/kg. This dose was then used to determine the possible interaction of compounds **4c** and **4k** with the benzodiazepine binding site of the GABA_A_ receptor.

For the second part of the assay, 12 mice were weighed and divided into 2 groups (*n* = 6 mice for each group). All mice received 5 mg/kg of flumazenil i.p., followed by compound **4c** for group 1 and compound **4k** for group 2, 5 min later. The administered i.p. dose was based on the ED_50_ for each of both compounds. Following the 30 min incubation period, the mice received 75 mg/kg of PTZ i.p. and were monitored identically as in the first part of the assay.

The results were quantified as the latency until first seizure (Ti), number of seizures, and protection against tonic–clonic lethal seizures (PTZ P/T). The last parameter was calculated as a ratio between the number of mice protected from seizures and the number of tested mice in a group (*n* = 6) and expressed as a percentage (%).

### 4.4. Statistical Analysis

The statistical analysis was performed using GraphPad Prism 8.0.2 software (Windows version 8.0.2., GraphPad Software Inc., San Diego, CA, USA) and IBM SPSS Statistics 27.0.1.0 software (IBM, Armonk, NY, USA). For quantifying the results of the initial PTZ-induced seizure model in a single dose, the treated mice, either with compounds **4a-o** or with reference drugs, were compared with the mice in the negative control group by applying the ordinary one-way analysis of variance (ANOVA) test, followed by post hoc Dunnett’s multiple comparison, when comparing the number of seizures.

The results were expressed as mean ± standard error of mean (SEM) for table and graphical representation. The comparisons between the treated groups and the negative control group were considered statistically significant if the *p* value was lower than 0.05 (*p* < 0.05). The evaluated parameters were the latency until first seizure (Ti), number of seizures, and protection against tonic–clonic lethal seizures (PTZ P/T). The same analysis was applied for quantifying the results of the PTZ-induced seizure model in four doses of compounds **4c** and **4k**, respectively, for the flumazenil antagonism assay [22].

Seizure latency (time to first seizure onset, Ti) was analyzed as a time-to-event variable using Kaplan–Meier survival analysis. Animals that remained seizure-free throughout the 30 min (Ti = 1800 s) observation period were recorded as censored observations. Overall differences among groups were assessed using the log-rank test (Mantel–Cox). Post hoc pairwise comparisons vs. negative control were performed using pairwise log-rank tests with Bonferroni correction (17 comparisons). Results are presented as median latency [95% CI] and Kaplan–Meier survival plots. All analyses were performed using IBM SPSS Statistics (version 27.0.1.0). Statistical significance was set at *p* < 0.05.

## 5. Conclusions

In this study, 15 novel compounds (**4a-o**) were designed, synthesized, and evaluated for their anticonvulsant potential using the PTZ-induced seizure model on mice. A Rotarod test was used to evaluate the neuromotor coordination following the administration of compounds **4a-o**. A flumazenil antagonism assay was performed to study the possible in vivo interaction of the compounds (**4c** and **4k**) with the GABA_A_ receptor. The anticonvulsant evaluation was completed with an in silico molecular docking study on the human α_1_β_2_γ_2_ GABA_A_ receptor and on the NR1 ligand-binding core of the NMDA receptor. Druggability and ADMETox prediction studies were employed to further explore the potential of these compounds as possible drugs and to study the possibility of these compounds to penetrate the BBB.

The chemical design of compounds **4a-o** was based on reuniting the thiazole and quinazolin-4(3*H*)-one heterocycles of clomethiazole and methaqualone, two well-documented anticonvulsants, into a single scaffold. The synthesis of these compounds was based on a condensation reaction between the heterocycle-containing intermediates. All compounds were confirmed through spectral analysis.

The compounds had overall good druggability properties and had good predictions regarding BBB permeation capacity, thus offering the ground for the possibility to exert anticonvulsant activity. A potential risk of drug–drug interactions and adverse effects was predicted from the ADMETox simulations.

Based on the molecular docking study, the compounds had an affinity for the selected targets. Overall, the aromatic series **4g-o** had better affinity for both targets compared to the aliphatic series **4a-f**. The most frequent interactions observed between the ligands and the proteins were hydrogen bonds and π-π stackings. While there was a degree of correlation between the in silico and in vivo results, it is also possible that other mechanisms that were not covered in the study could contribute to the anticonvulsant effect.

All tested compounds showed anticonvulsant activity with various potencies and none of them induced neuromotor coordination impairment. The most favorable substituents for the activity were *p*-methoxyphenyl (**4k**), allyl (**4c**), methyl (**4a**), benzyl (**4h**), and *p*-fluorophenyl (**4i**). Compounds **4c** and **4k** emerged as the most potent anticonvulsants and were further evaluated to determine their ED_50_ values. The flumazenil antagonism assay preliminarily suggested that compounds **4c** and **4k** may act as PAMs of the GABA_A_ receptor. Compound **4k** was more potent than compound **4c** and, compared to the literature data, it acted as a more potent PAM of the GABA_A_ receptor than methaqualone and clomethiazole.

The current study represents a preliminary evaluation of the anticonvulsant activity of the tested compounds. While an in vivo flumazenil antagonism assay was performed to evidence compounds’ potential as PAMs of GABA_A_, along with in silico molecular docking studies on GABA_A_ and NMDA receptors, in vitro affinity assays on the isolated targets are necessary to conclude the potential of these compounds as PAMs of GABA_A_ and NMDA antagonists. Further testing is necessary to define the complete pharmacological and toxicological profiles of these compounds.

## Figures and Tables

**Table 1:** The in silico physicochemical prediction performed using the SwissADME web tool. The prediction of logD at pH = 7.4 was performed using Deep-PK and for LogBB by LogBB_Pred online application.

Compound	MW (g/mol)	No. of RBs	No. of HBAs	No. of HBDs	TPSA (Å^2^)	MLogP	LogD at pH = 7.4	LogBB	ESOL	No. of Lipinski Violations
**4a**	365.47	4	3	0	101.32	2.94	4.01	−0.28247	−5.01	0
**4b**	379.50	5	3	0	101.32	3.17	4.52	−0.32344	−5.24	0
**4c**	391.51	6	3	0	101.32	3.32	4.55	−0.40209	−5.40	0
**4d**	393.53	5	3	0	101.32	3.39	4.50	−0.33999	−5.57	0
**4e**	407.55	7	3	0	101.32	3.61	4.89	−0.21749	−5.79	0
**4f**	433.59	5	3	0	101.32	4.44	5.83	−0.04630	−6.40	1
**4g**	427.54	5	3	0	101.32	4.50	4.47	−0.33006	−6.35	1
**4h**	441.57	6	3	0	101.32	4.44	4.84	−0.24951	−6.32	1
**4i**	445.53	5	4	0	101.32	4.88	4.27	−0.25908	−6.51	1
**4j**	461.99	5	3	0	101.32	4.98	4.35	−0.42819	−6.94	1
**4k**	457.57	6	4	0	110.55	3.48	4.10	−0.43007	−6.41	0
**4l**	457.57	6	4	0	110.55	3.48	4.18	−0.41638	−6.41	0
**4m**	495.54	6	6	0	101.32	5.02	4.76	−0.05344	−7.19	1
**4n**	487.59	7	5	0	119.78	3.14	3.52	−0.33006	−6.48	0
**4o**	496.43	5	3	0	101.32	5.45	4.74	−0.62800	−7.53	1
**DZP**	284.74	1	2	0	32.67	2.67	2.80	0.57460	−3.87	0
**PHE**	230.22	2	5	0	51.21	1.77	2.65	−0.10003	−2.32	0
**CMT**	161.65	2	1	0	41.13	1.19	1.36	−0.00979	−2.46	0
**MTQ**	250.30	1	2	0	34.89	2.99	2.46	−0.02719	−3.52	0
**PTZ**	138.17	0	3	0	43.60	1.02	−0.09	−0.01235	−3.52	0

Legend: MW—molecular weight; No. of RBs—number of rotatable bonds; No. of HBAs—number of hydrogen bond acceptors; No. of HBDs—number of hydrogen bond donors; TPSA—topological polar surface area; MLogP—the octanol–water partition coefficient implemented by Moriguchi; logD—pH-dependent LogP; LogBB—logarithmic ratio between the concentration of a drug in the brain and the concentration of the same drug in the blood; ESOL—estimated solubility; DZP—diazepam; PHE—phenobarbital; CMT—clomethiazole; MTQ—methaqualone; PTZ—pentylenetetrazole.

**Table 2:** The in silico pharmacokinetic descriptors that were computed for compounds **4a-o**, references, and PTZ. The prediction was performed using the Deep-PK online platform. Confidence level legend: ***—high confidence; **—medium confidence; *—low confidence.

Compound	GI Absorption	P-gp Substrate	P-gp Inhibitor	BBB Penetration	CYP1A2 Inhibitor	CYP2C19 Inhibitor	CYP2C9 Inhibitor	CYP2D6 Inhibitor	CYP3A4 Inhibitor	Half-Life
**4a**	Yes ***	No ***	Yes ***	Yes ***	Yes ***	Yes **	Yes ***	No ***	Yes *	<3h ***
**4b**	Yes ***	No ***	Yes ***	Yes ***	Yes ***	Yes ***	Yes ***	No ***	Yes **	<3h ***
**4c**	Yes ***	No ***	Yes ***	Yes ***	Yes ***	Yes ***	Yes ***	No ***	Yes ***	<3h ***
**4d**	Yes ***	No ***	Yes ***	Yes ***	Yes ***	Yes **	Yes ***	No ***	Yes ***	<3h ***
**4e**	Yes ***	No **	Yes ***	Yes ***	Yes ***	Yes ***	Yes ***	No ***	Yes ***	<3h ***
**4f**	Yes ***	No **	Yes ***	Yes ***	Yes ***	Yes **	Yes ***	No ***	Yes ***	<3h ***
**4g**	Yes ***	No ***	Yes ***	Yes ***	Yes ***	Yes ***	Yes ***	No ***	Yes **	<3h ***
**4h**	Yes ***	No ***	Yes ***	Yes ***	Yes ***	Yes ***	Yes ***	No ***	Yes ***	<3h ***
**4i**	Yes ***	No ***	Yes ***	Yes ***	Yes ***	Yes **	Yes ***	No ***	Yes **	<3h ***
**4j**	Yes ***	No ***	Yes ***	Yes ***	Yes ***	Yes ***	Yes ***	No ***	Yes **	<3h ***
**4k**	Yes ***	No ***	Yes ***	Yes ***	Yes ***	Yes *	Yes ***	No ***	Yes ***	<3h ***
**4l**	Yes ***	No ***	Yes ***	Yes ***	Yes ***	Yes ***	Yes ***	No ***	Yes ***	<3h ***
**4m**	Yes ***	No ***	Yes ***	Yes ***	Yes ***	Yes ***	Yes ***	No ***	Yes **	<3h ***
**4n**	Yes ***	No ***	Yes ***	Yes ***	Yes **	Yes ***	Yes ***	No ***	Yes ***	<3h ***
**4o**	Yes ***	No ***	Yes ***	Yes ***	Yes ***	Yes **	Yes ***	No ***	Yes *	<3h ***
**DZP**	Yes ***	No ***	No ***	Yes ***	Yes ***	Yes ***	No ***	No ***	No ***	<3h ***
**PHE**	Yes ***	No ***	No ***	Yes ***	No ***	No ***	No ***	No ***	No ***	<3h ***
**CMT**	Yes ***	No ***	No ***	Yes ***	Yes ***	Yes ***	No ***	Yes ***	No ***	<3h **
**MTQ**	Yes ***	No **	No **	Yes ***	No *	No *	No ***	No ***	No ***	<3h **
**PTZ**	Yes ***	No **	Yes ***	Yes ***	Yes **	No ***	No ***	No ***	No ***	<3h ***

**Table 3:** The in silico toxicological descriptors that were computed for compounds **4a-o**, references, and PTZ. The prediction was performed using the Deep-PK online platform. Confidence level legend: ***—high confidence; **—medium confidence; *—low confidence.

Compound	Carcinogenesis	Eye Irritation	Hepatotoxicity	Respiratory Toxicity	Skin Sensitization	Rat Acute Toxicity	Rat Chronic Oral Toxicity
**4a**	Toxic *	Safe ***	Toxic ***	Toxic **	Toxic *	None	None
**4b**	Toxic *	Safe ***	Toxic ***	Safe **	Toxic *	None	None
**4c**	Toxic *	Safe ***	Toxic ***	Safe *	Safe *	None	None
**4d**	Toxic *	Safe ***	Toxic ***	Safe **	Toxic *	None	None
**4e**	Toxic *	Safe ***	Toxic ***	Safe ***	Toxic *	None	None
**4f**	Safe *	Safe ***	Toxic ***	Safe ***	Toxic *	None	None
**4g**	Toxic *	Safe ***	Toxic ***	Safe *	Toxic *	None	None
**4h**	Toxic *	Safe ***	Toxic ***	Safe **	Toxic *	None	None
**4i**	Toxic *	Safe ***	Toxic ***	Toxic *	Safe *	None	None
**4j**	Toxic *	Safe ***	Toxic ***	Safe *	Toxic *	None	None
**4k**	Safe *	Safe ***	Toxic ***	Toxic *	Safe *	None	None
**4l**	Safe *	Safe ***	Toxic ***	Toxic *	Safe *	None	None
**4m**	Toxic *	Safe ***	Toxic ***	Safe **	Toxic *	None	None
**4n**	Safe *	Safe ***	Toxic ***	Toxic *	Safe *	None	None
**4o**	Toxic *	Safe ***	Toxic ***	Toxic *	Toxic *	None	None
**DZP**	Safe **	Safe ***	Toxic **	Toxic **	Toxic *	None	None
**PHE**	Toxic *	Safe ***	Safe *	Toxic *	Toxic **	None	None
**CMT**	Safe *	Toxic *	Toxic **	Toxic ***	Toxic *	None	None
**MTQ**	Toxic *	Safe *	Toxic **	Toxic *	Toxic *	None	None
**PTZ**	Safe ***	Toxic **	Toxic *	Toxic ***	Toxic **	None	None

**Table 4:** The results of the molecular docking of compounds **4a-o** to the GABA_A_ and NMDA receptors expressed as the variation in Gibbs free energy (ΔG, kcal/mol).

Compound	ΔG (kcal/mol)	
	GABA_A_	NMDA
**4a**	−9.8	−8.9
**4b**	−9.4	−8.2
**4c**	−9.6	−8.6
**4d**	−9.2	−8.6
**4e**	−9.4	−8.1
**4f**	−11.4	−9.0
**4g**	−11.0	−9.4
**4h**	−8.5	−9.0
**4i**	−10.9	−9.7
**4j**	−9.2	−9.7
**4k**	−9.0	−9.3
**4l**	−9.4	−9.2
**4m**	−9.5	−9.8
**4n**	−8.6	−9.2
**4o**	−9.2	−9.4

**Table 5:** Anticonvulsant activity and impaired neuromotor coordination potential of the compounds **4a-o**.

Group	PTZ ^a^		RR ^b^	
	P/T	%Protection	B/T	%Preserved
**NC**	0/6	0	ND	ND
**4a**	5/6	83.33	6/6	100
**4b**	2/6	33.33	6/6	100
**4c**	5/6	83.33	6/6	100
**4d**	4/6	66.66	6/6	100
**4e**	4/6	66.66	6/6	100
**4f**	4/6	66.66	6/6	100
**4g**	2/6	33.33	6/6	100
**4h**	5/6	83.33	6/6	100
**4i**	5/6	83.33	6/6	100
**4j**	4/6	66.66	6/6	100
**4k**	6/6	100	6/6	100
**4l**	2/6	33.33	6/6	100
**4m**	4/6	66.66	6/6	100
**4n**	3/6	50	6/6	100
**4o**	4/6	66.66	6/6	100
**DZP** (2 mg/kg)	6/6	100	6/6	100
**PHE** (15 mg/kg)	6/6	100	6/6	100

Legend: NC—negative control; ^a^ PTZ—pentylenetetrazole (75 mg/kg, i.p.)-induced lethal seizure; (P)—number of protected mice/(T)—number of mice tested; RR B/T—preserved neuromotor coordination; ^b^ neuromotor coordination evaluated by Rotarod test (6 rpm, 180 s cutoff time); (B)—number of mice that maintained their balance; ND—not determined.

**Table 6:** Anticonvulsant activity and impaired neuromotor coordination potential for compounds **4c** and **4k** in four different doses.

Group	PTZ ^a^		RR ^b^	
	P/T	%Protection	B/T	%Preserved
**NC**	0/6	0	ND	ND
**Compound 4c (ED_50_ = 178.165 mg/kg)**				
**D_1_ = 50 mg/kg**	3/6	50	6/6	100
**D_2_ = 150 mg/kg**	3/6	50	6/6	100
**D_3_ = 300 mg/kg**	4/6	66.66	6/6	100
**D_4_ = 450 mg/kg**	2/6	33.33	6/6	100
**Compound 4k (ED_50_ = 84.313 mg/kg)**				
**D_1_ = 50 mg/kg**	3/6	50	6/6	100
**D_2_ = 150 mg/kg**	3/6	50	6/6	100
**D_3_ = 300 mg/kg**	3/6	50	6/6	100
**D_4_ = 450 mg/kg**	1/6	16.66	6/6	100

Legend: NC—negative control; ^a^ PTZ—pentylenetetrazole (75 mg/kg, i.p.)-induced lethal seizure; (P)—number of protected mice/(T)—number of mice tested; RR B/T—preserved neuromotor coordination; ^b^ neuromotor coordination evaluated by Rotarod test (6 rpm, 180 s cutoff time); (B)—number of mice that maintained their balance; ND—not determined.

## Data Availability

The data presented in this study are available in this article.

## Supplementary Materials

The following supporting information can be downloaded at: https://www.mdpi.com/article/10.3390/ijms27146107/s1, Figures S1–S30: The IR spectra for compounds 3a-o and 4a-o; Figures S31–S60: The MS for compounds 3a-o and 4a-o; Figures S61–S75: The 1H-NMR spectra for compounds 3a-o and 4a-o; Figures S76–S90: The 13C-NMR spectra for compounds 3a-o and 4a-o; Figures S91–S93: The predicted binding poses of compound 4f, 4g, and 4i in the human α1β2γ2 GABAA receptor; Table S1: The 3D-optimized structures of compounds 4a-o, diazepam, phenobarbital, clomethiazole, methaqualone, and pentylenetetrazole; Table S2: Seizure latency in PTZ-treated mice and statistical comparisons vs. negative control; Table S3: Mean number of seizures expressed as mean ± standard error of mean (SEM); Table S4: Latency until the first seizure (Ti) and mean number of seizures expressed as mean ± standard error of mean (SEM) for compounds 4c and 4k in four different doses; Table S5: Latency until the first seizure (Ti) and mean number of seizures expressed as mean ± standard error of mean (SEM) for compounds 4c and 4k and diazepam in the flumazenil antagonism assay.
