# Supplementary material for: Thiazol-4-yl-Methylthio-Quinazolin-4(3H)-ones as Anticonvulsant Compounds: Chemical Design, Computational Studies, and Biological Evaluation
Source: Int J Mol Sci. 2026 Jul 8;27(14):6107. doi: 10.3390/ijms27146107 (PMC13411530; doi:10.3390/ijms27146107)
Supplement: Supplementary file 1 [file ijms-27-06107-s001.zip › ijms-4405356-supplementary.pdf]

Article

Supplementary Material: Thiazol-4-yl-Methylthio-Quinazolin-4(3H)-ones as Anticonvulsant Compounds: Chemical Design, Computational Studies, and Biological Evaluation

Daniel Ungureanu [1], Anamaria Apan [2], Cristina Mogoșan [2,*], Radu Tamaian [3], Brîndușa Tiperciuc [1], Gabriel Marc [4], Raluca Pele [1], Laurian Vlase [5], Adrian Pîrnău [6], Cristina Moldovan [1], Ioana Ionuț [1], Anca Stana [1], Ovidiu Oniga [1]

1. Figures

1.1. IR Spectra

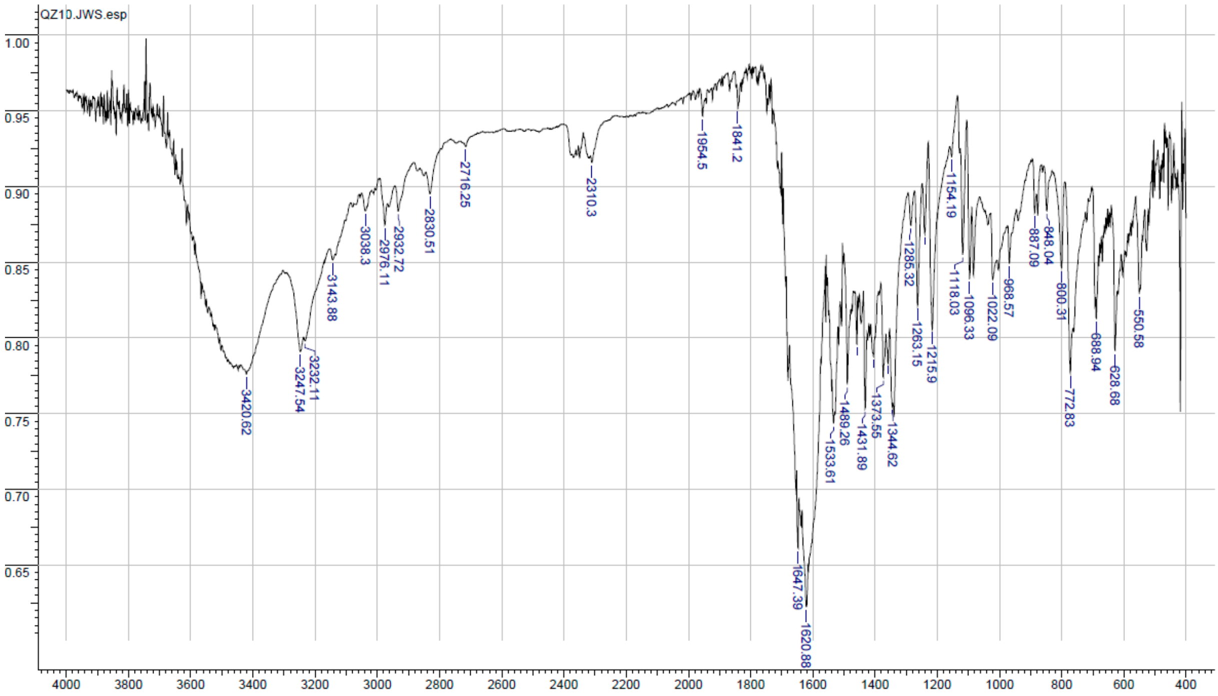

Figure S1. The IR spectrum for the compound **3a**.

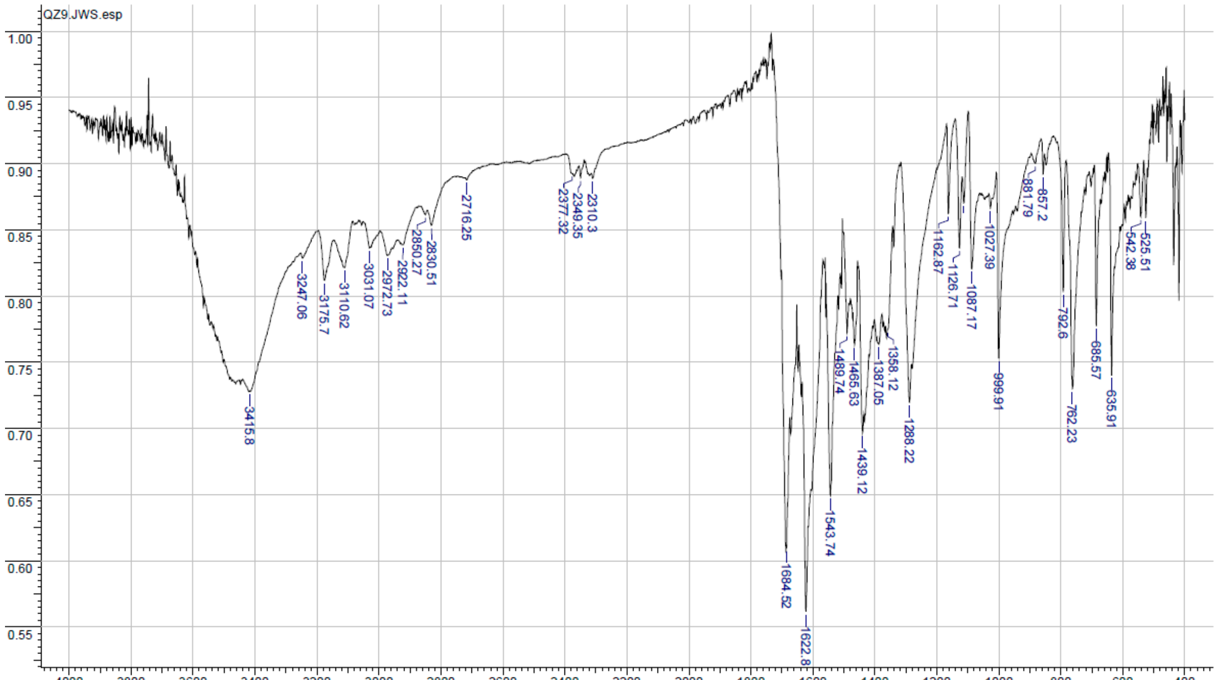

Figure S2. The IR spectrum for the compound **3b**.

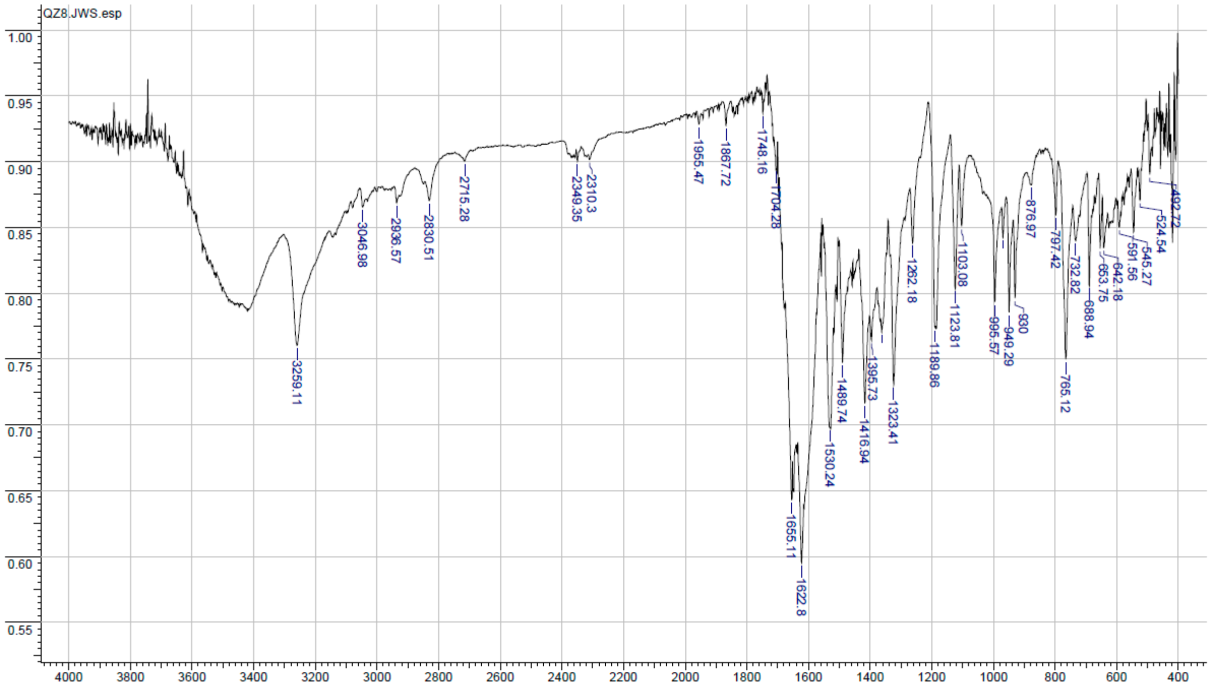

Figure S3. The IR spectrum for the compound **3c**.

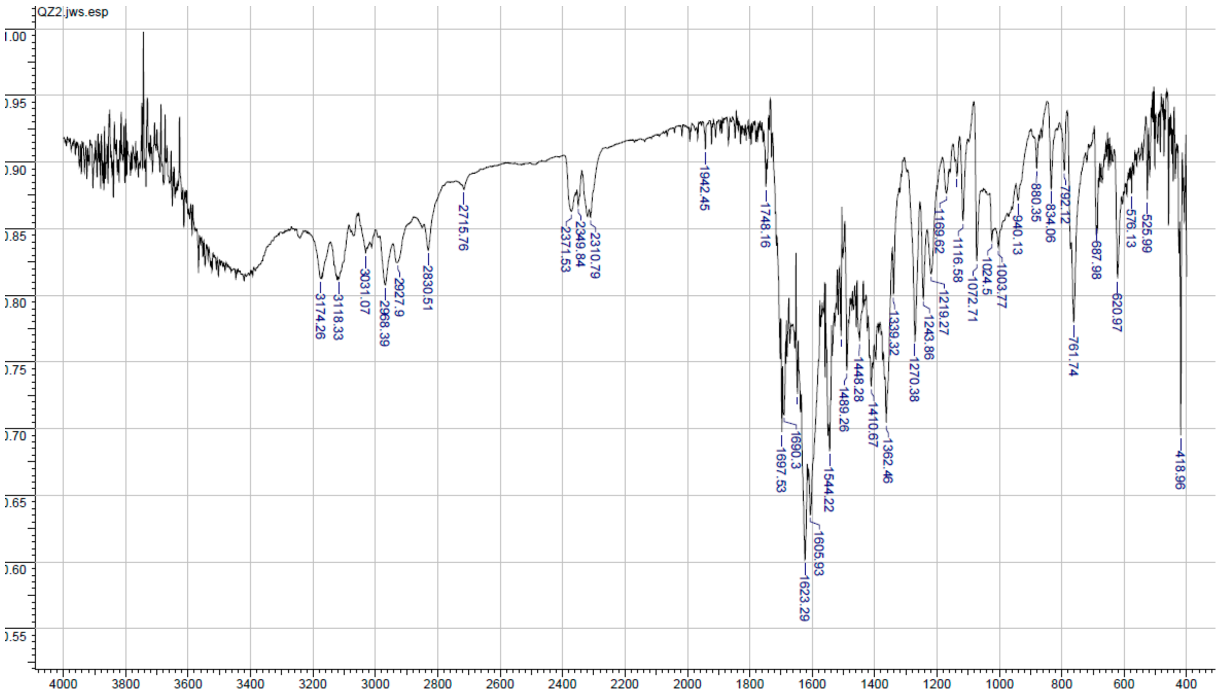

Figure S4. The IR spectrum for the compound **3d**.

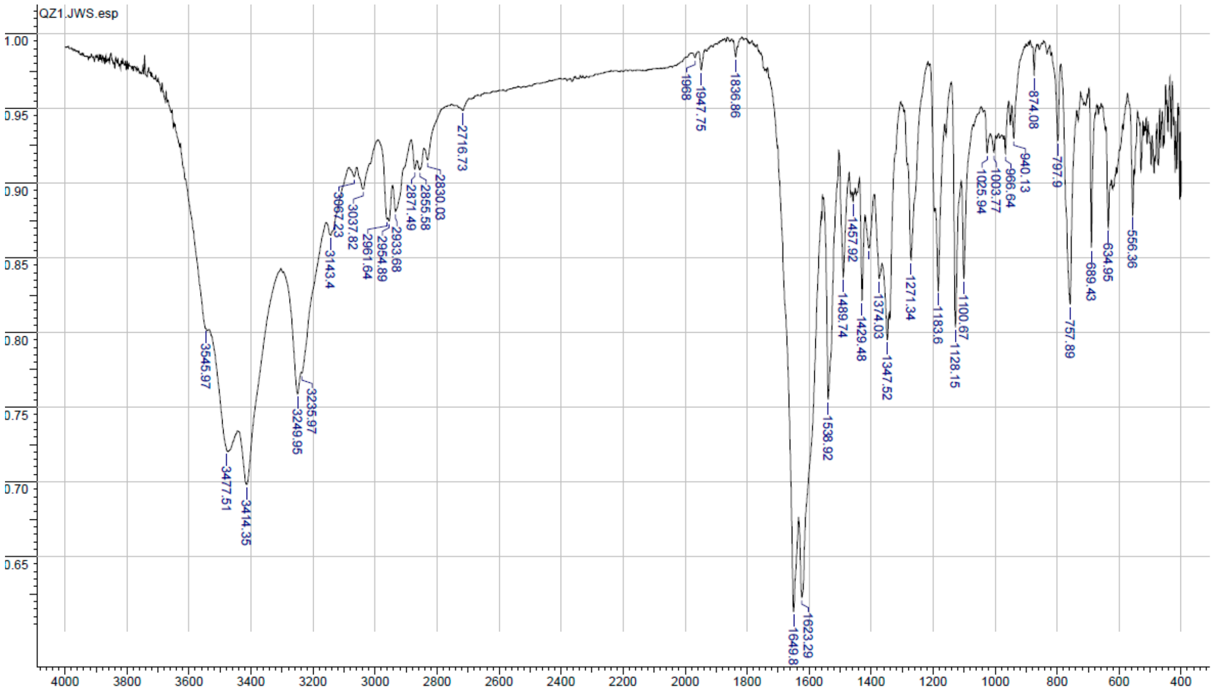

Figure S5. The IR spectrum for the compound **3e**.

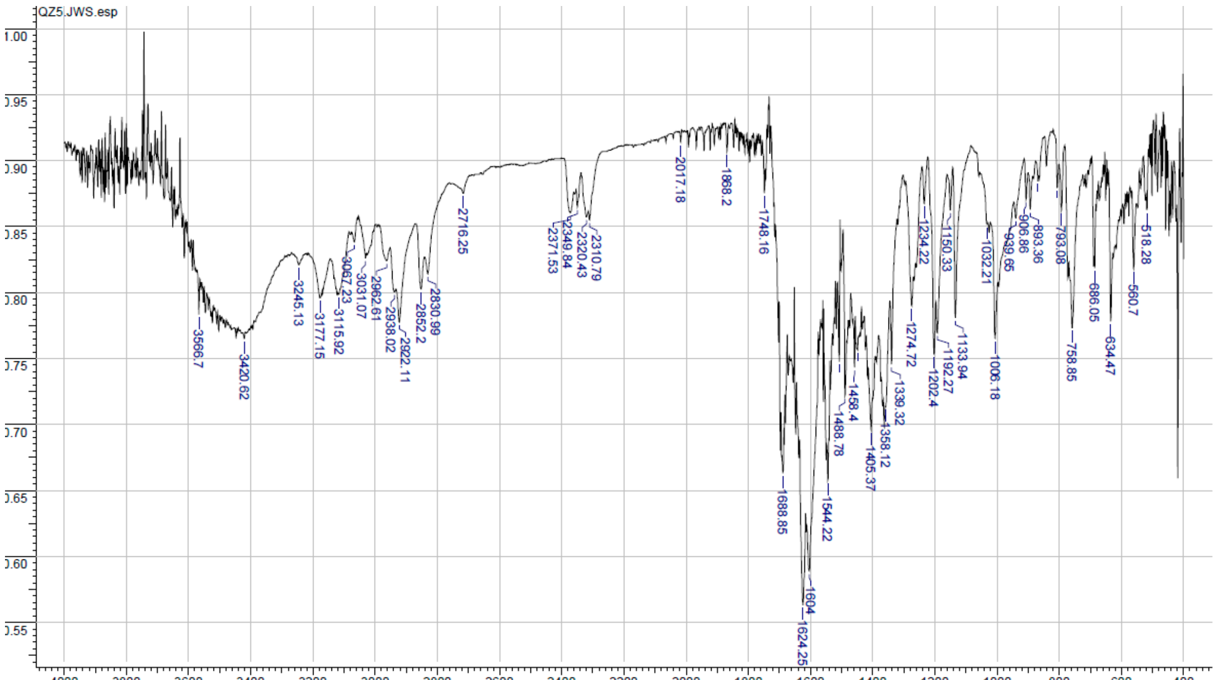

Figure S6. The IR spectrum for the compound **3f**.

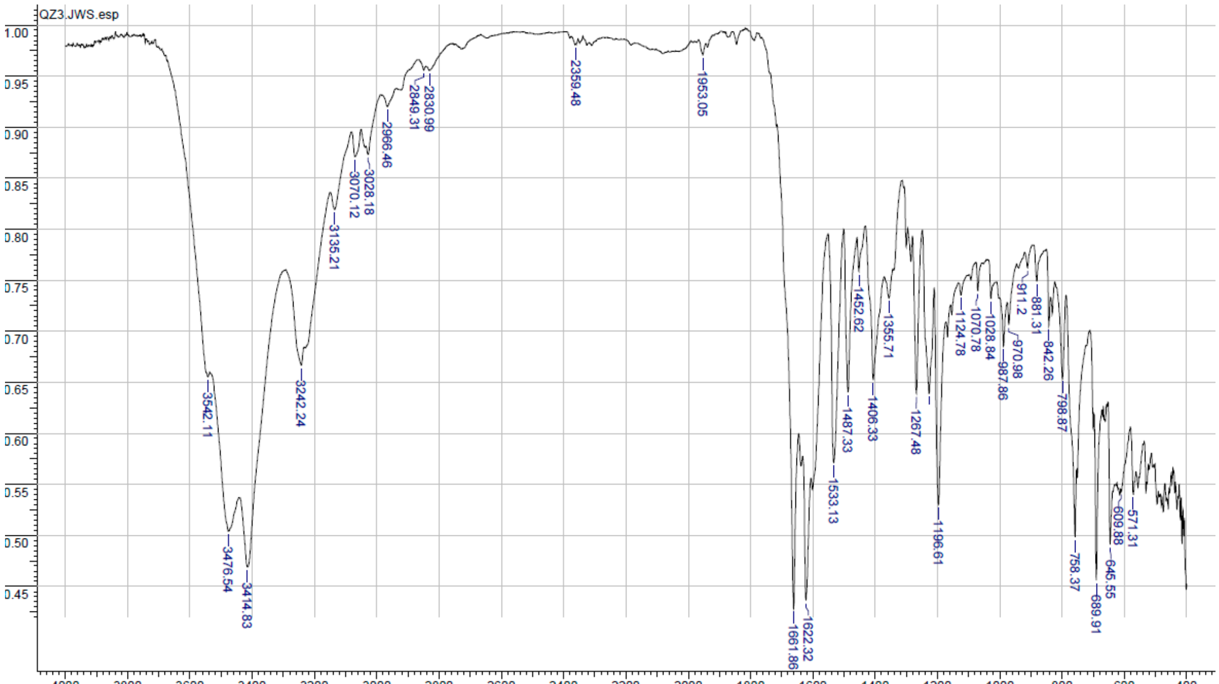

Figure S7. The IR spectrum for the compound **3g**.

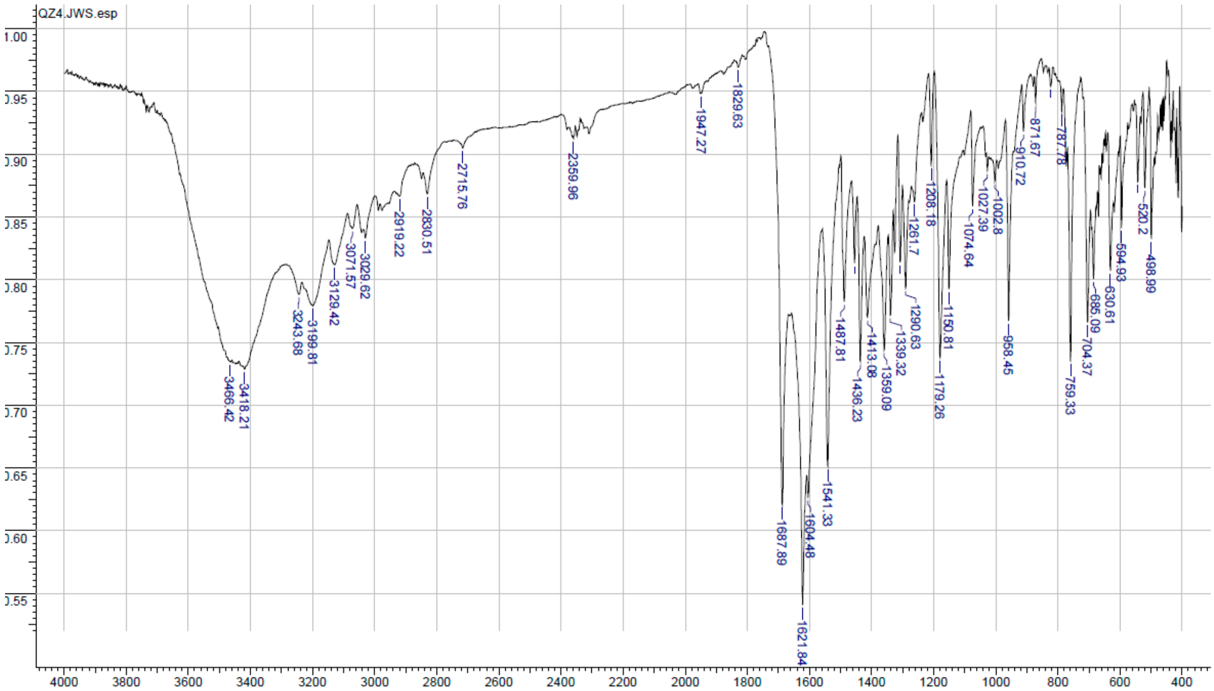

Figure S8. The IR spectrum for the compound **3h**.

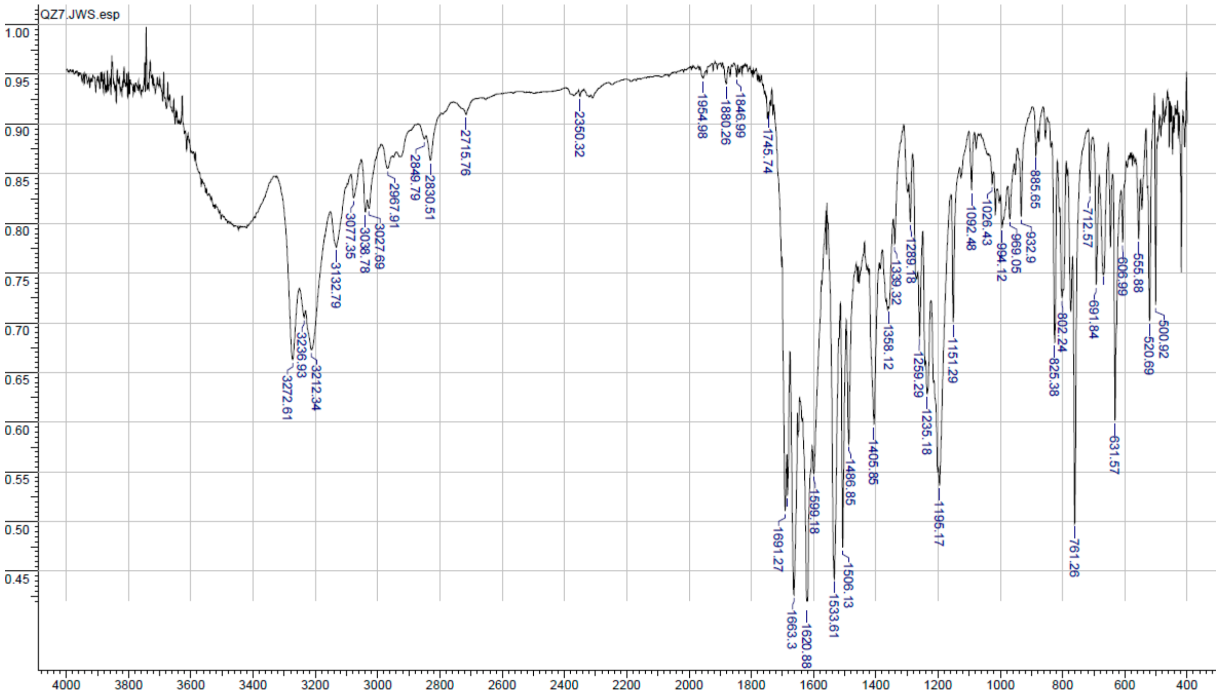

Figure S9. The IR spectrum for the compound **3i**.

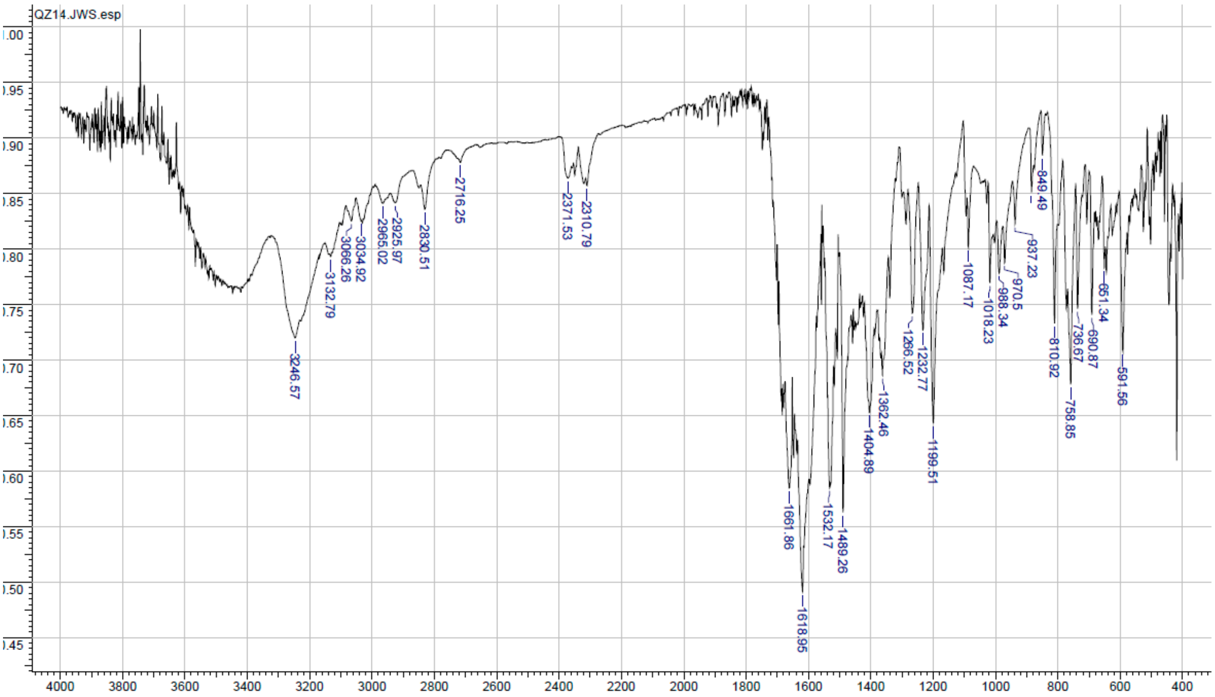

27

Figure S10. The IR spectrum for the compound **3j**. 28

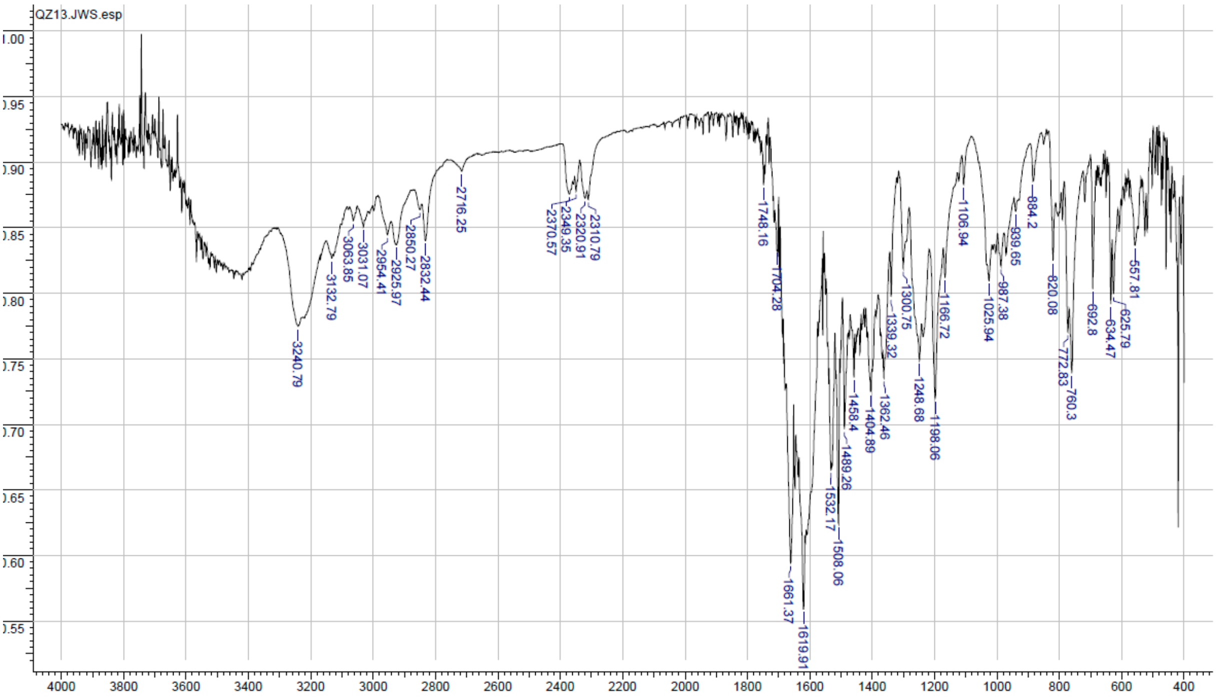

29

Figure S11. The IR spectrum for the compound **3k**. 30

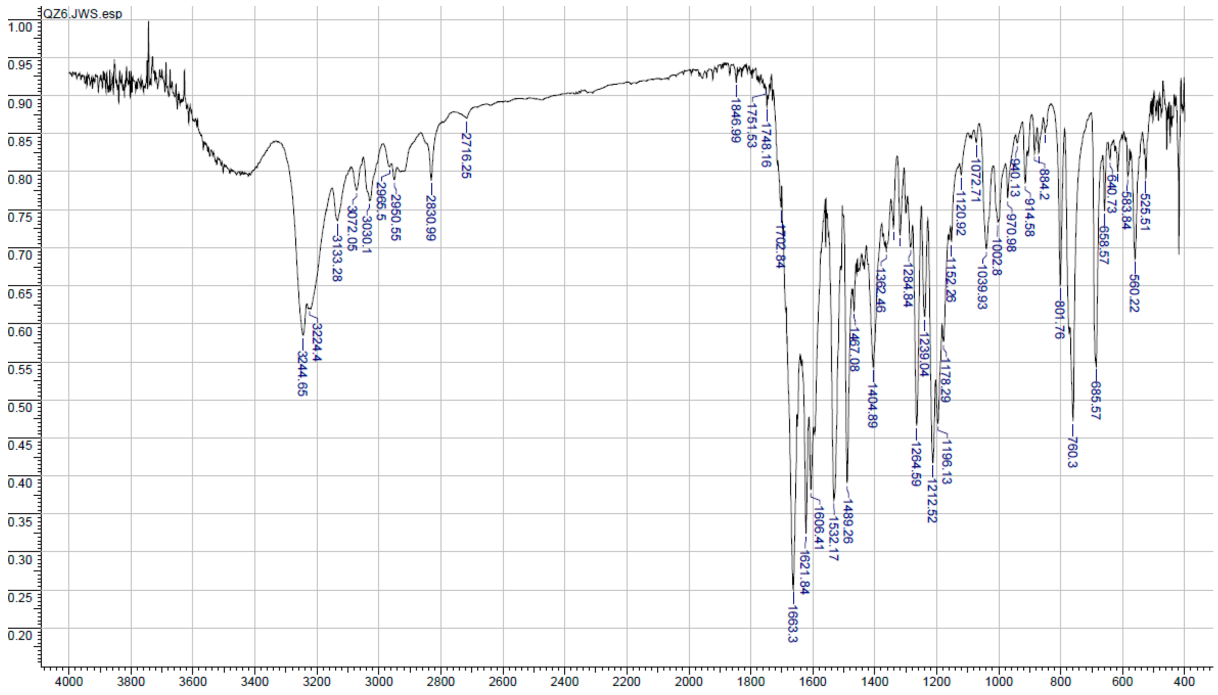

Figure S12. The IR spectrum for the compound **3l**.

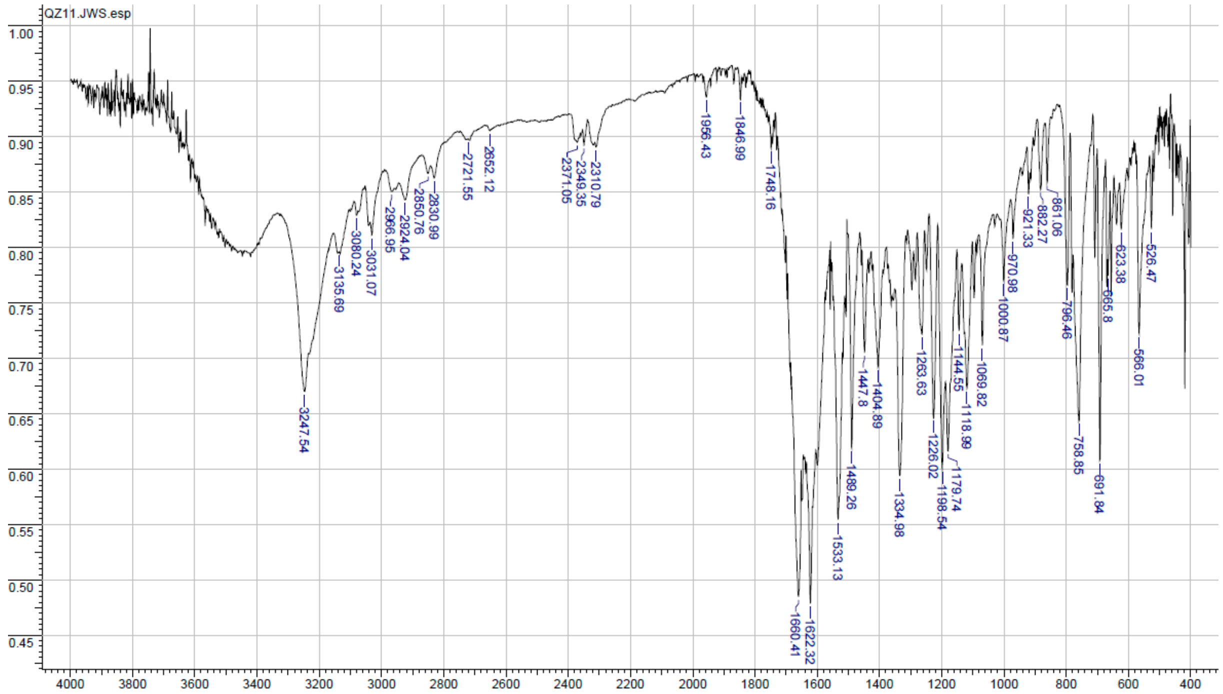

Figure S13. The IR spectrum for the compound **3m**.

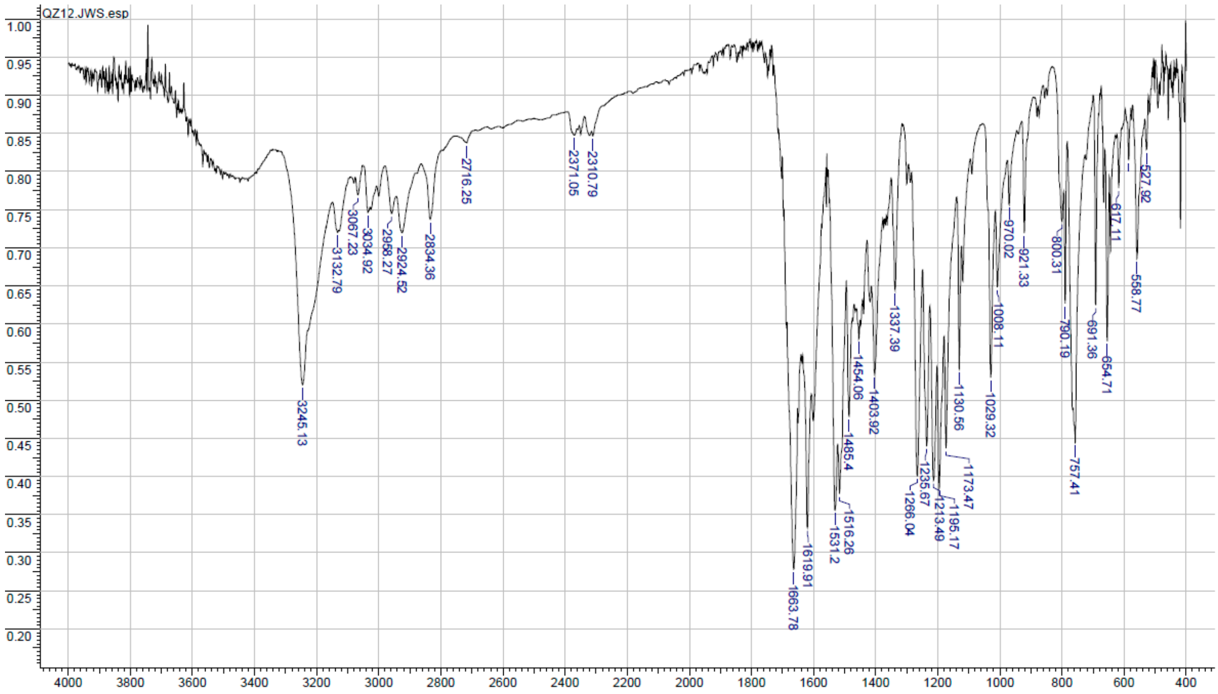

Figure S14. The IR spectrum for the compound **3n**.

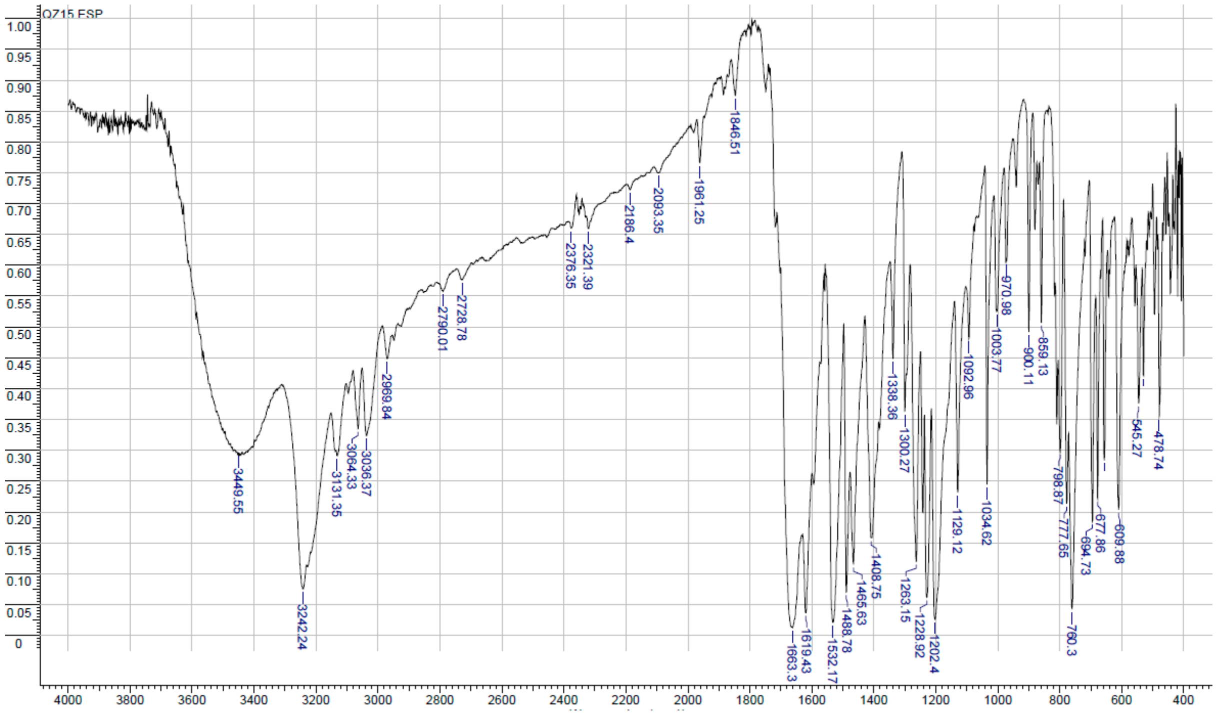

Figure S15. The IR spectrum for the compound **3o**.

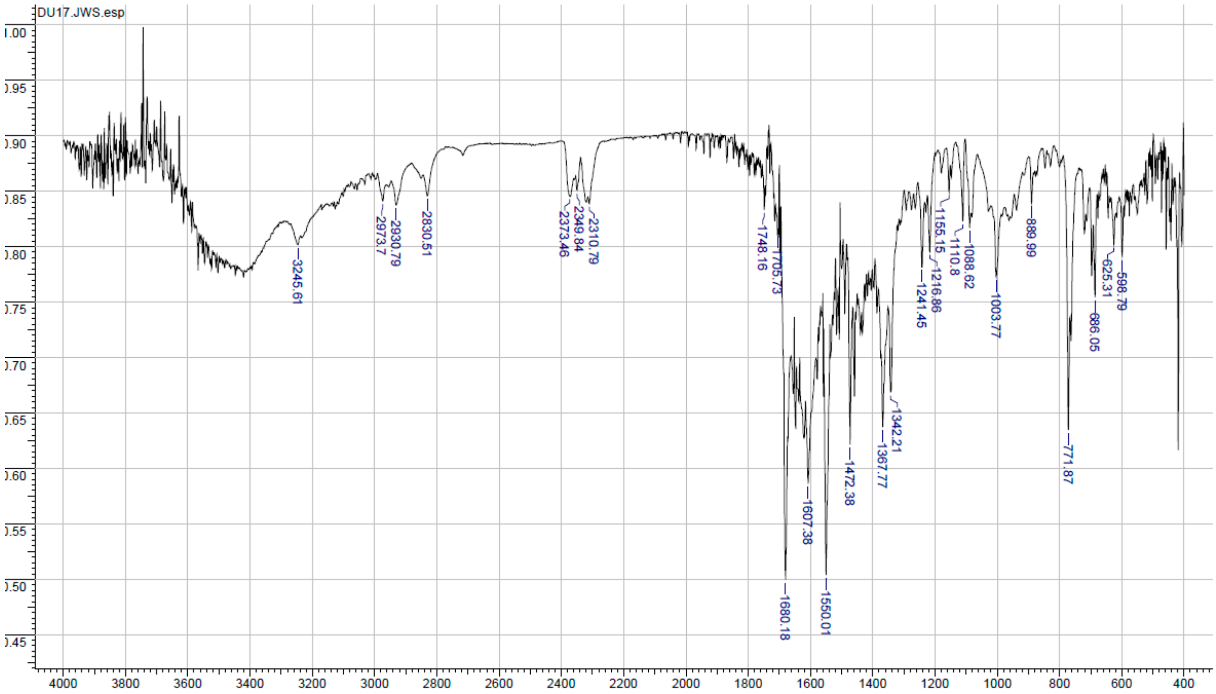

Figure S16. The IR spectrum for the compound **4a**.

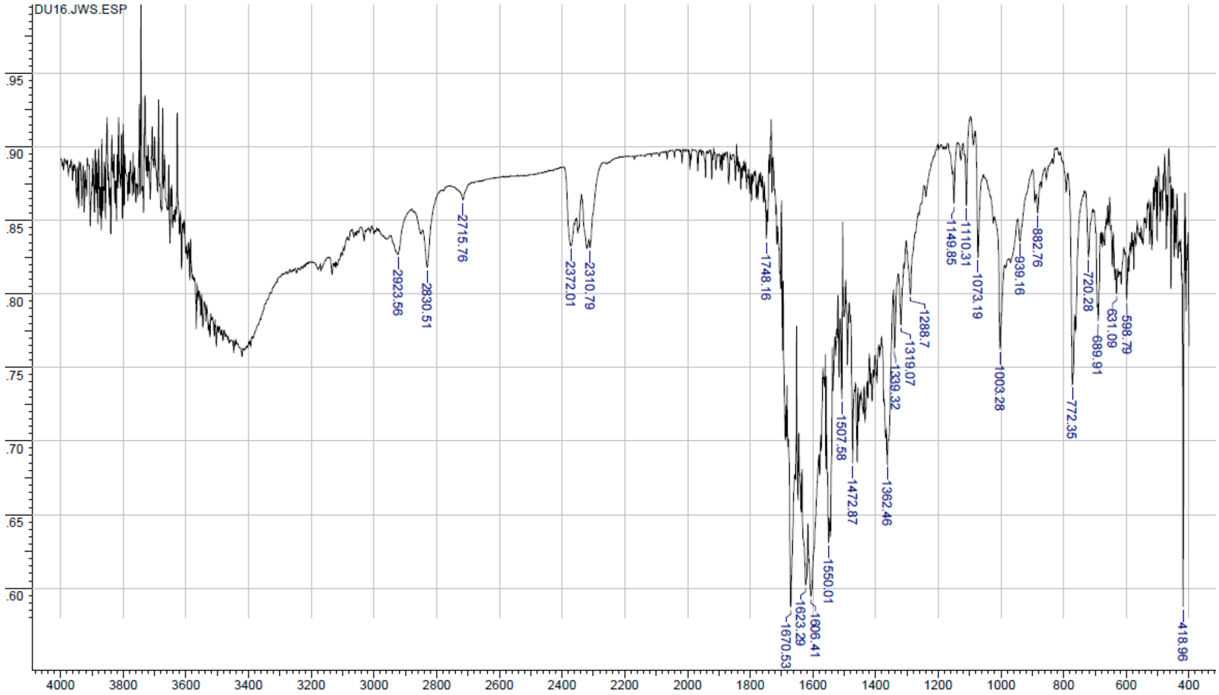

Figure S17. The IR spectrum for the compound **4b**.

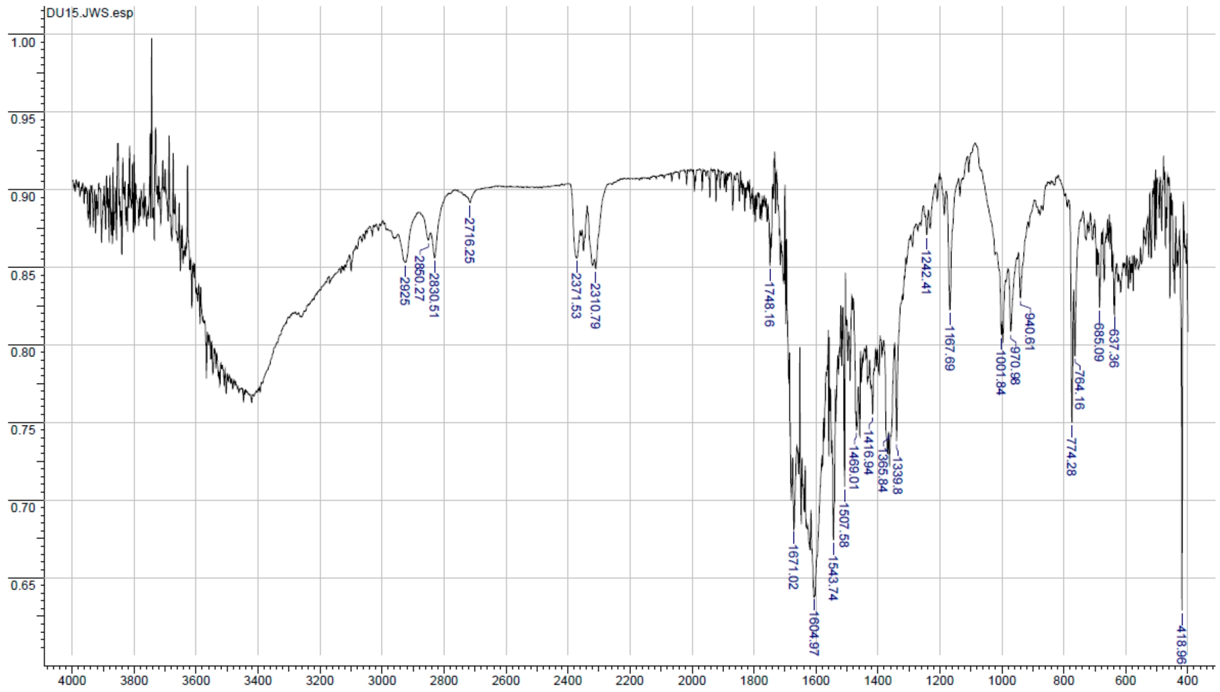

Figure S18. The IR spectrum for the compound **4c**.

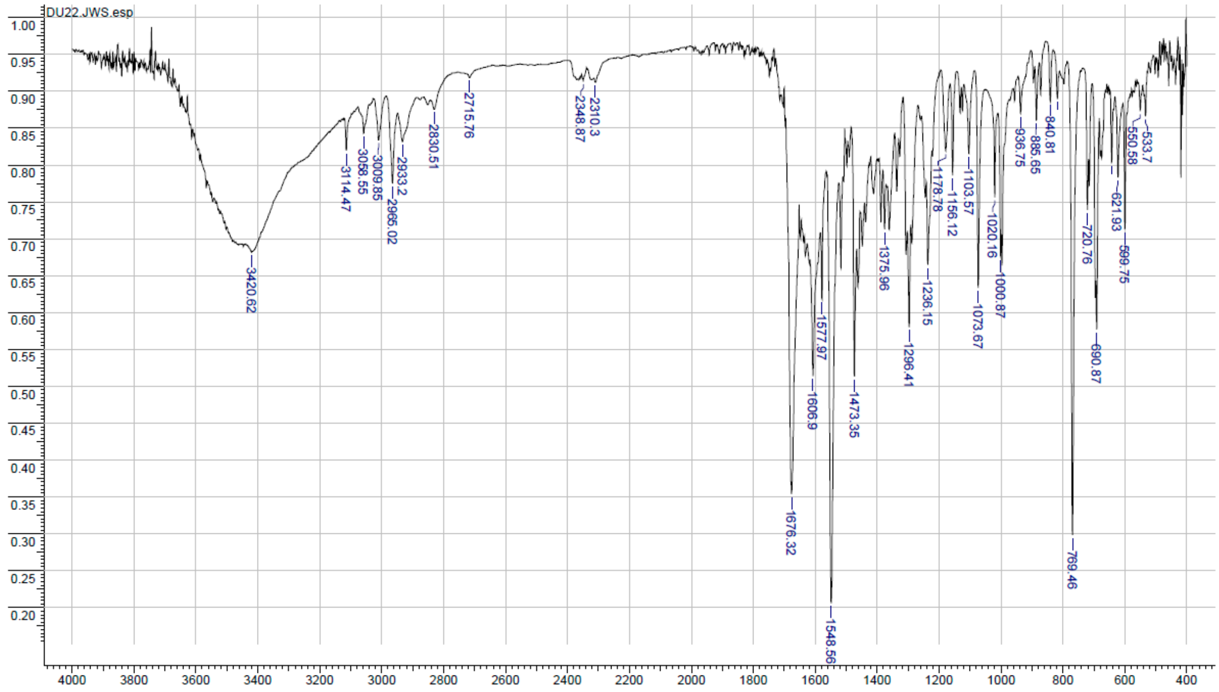

Figure S19. The IR spectrum for the compound **4d**.

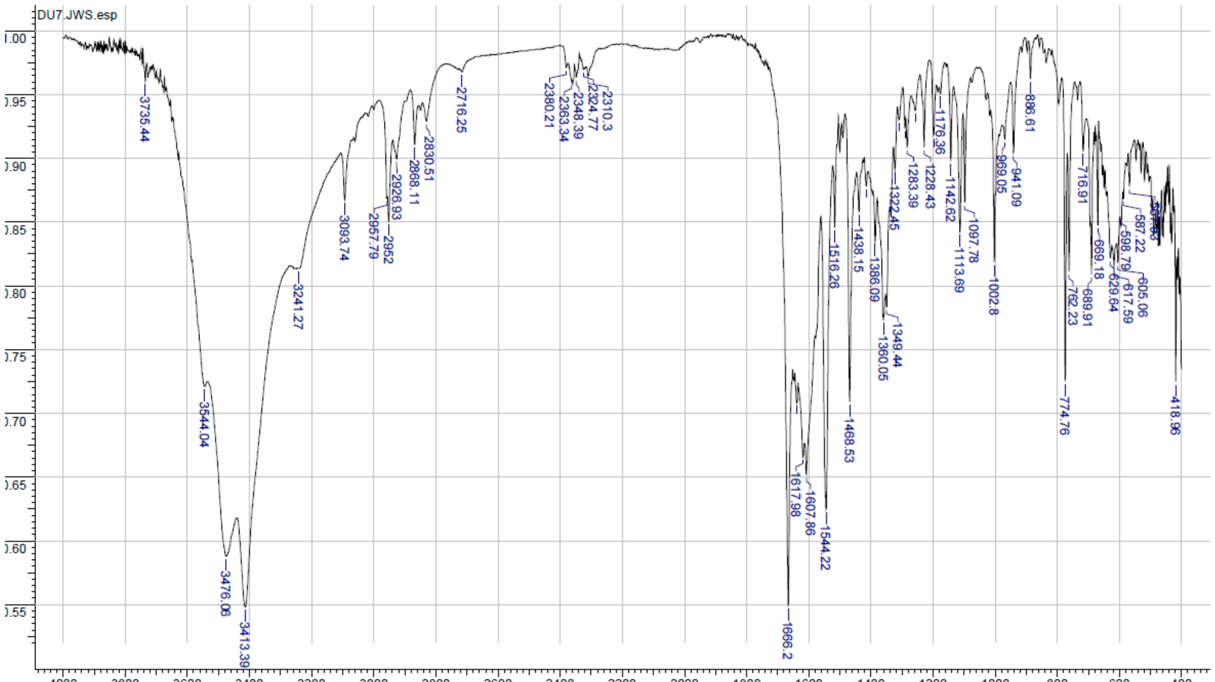

Figure S20. The IR spectrum for the compound **4e**.

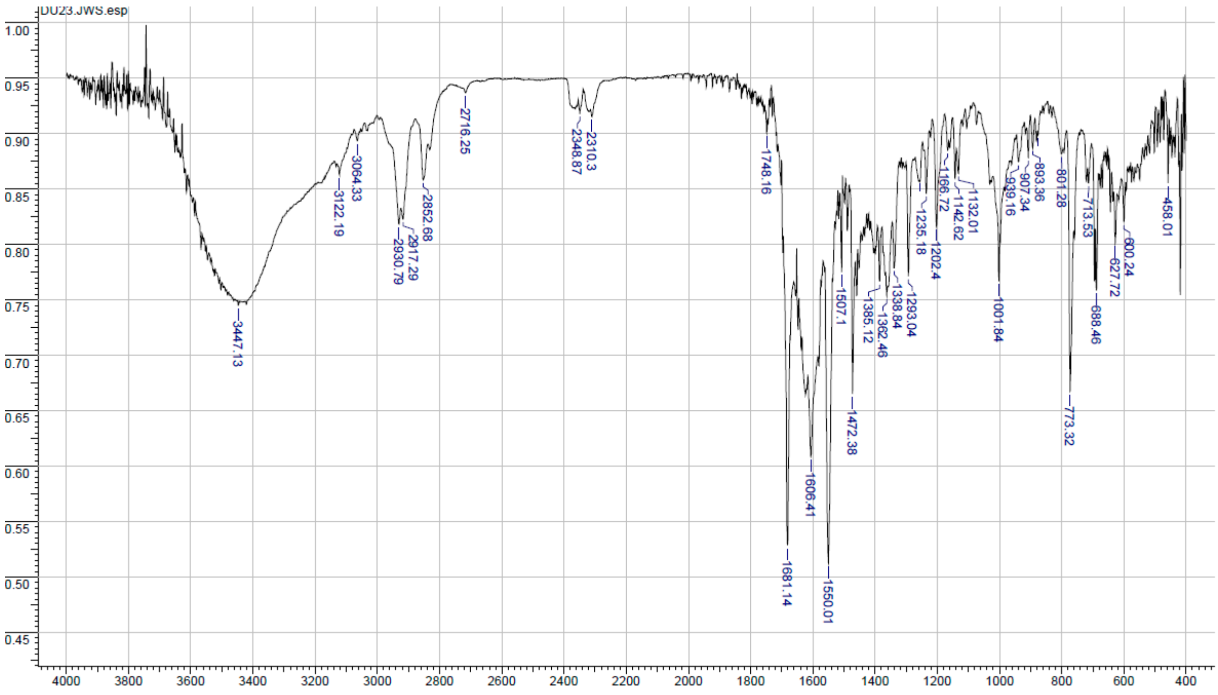

Figure S21. The IR spectrum for the compound **4f**.

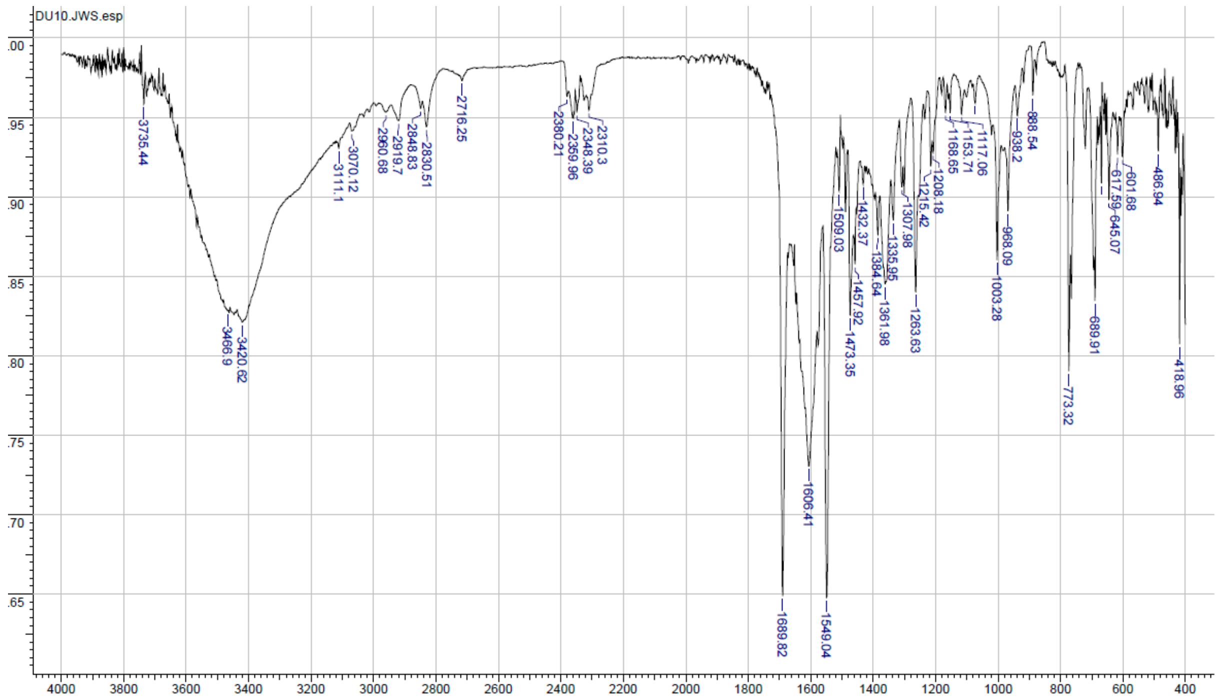

Figure S22. The IR spectrum for the compound **4g**.

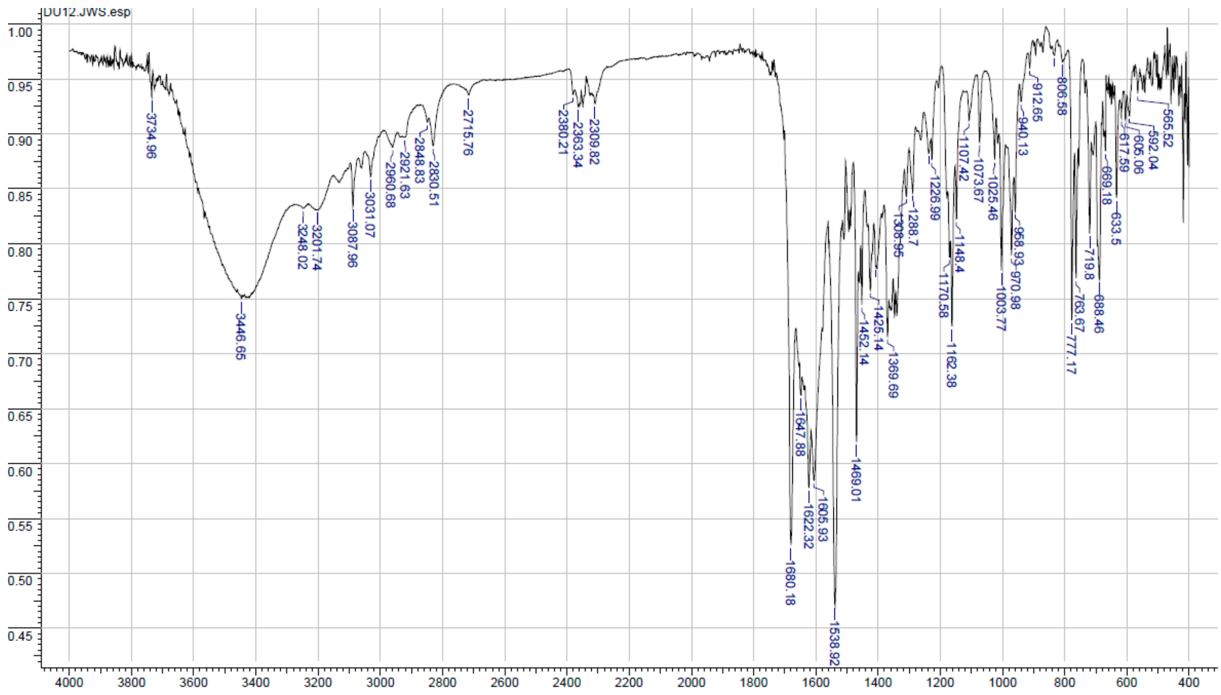

Figure S23. The IR spectrum for the compound **4h**.

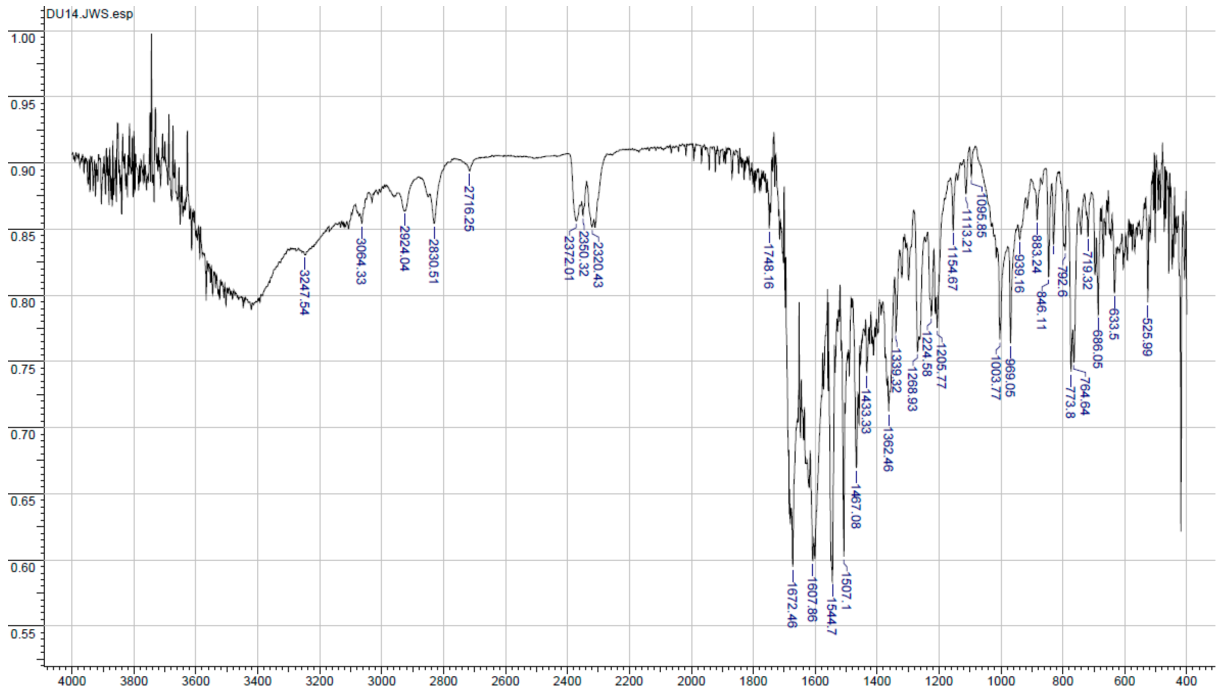

Figure S24. The IR spectrum for the compound **4i**.

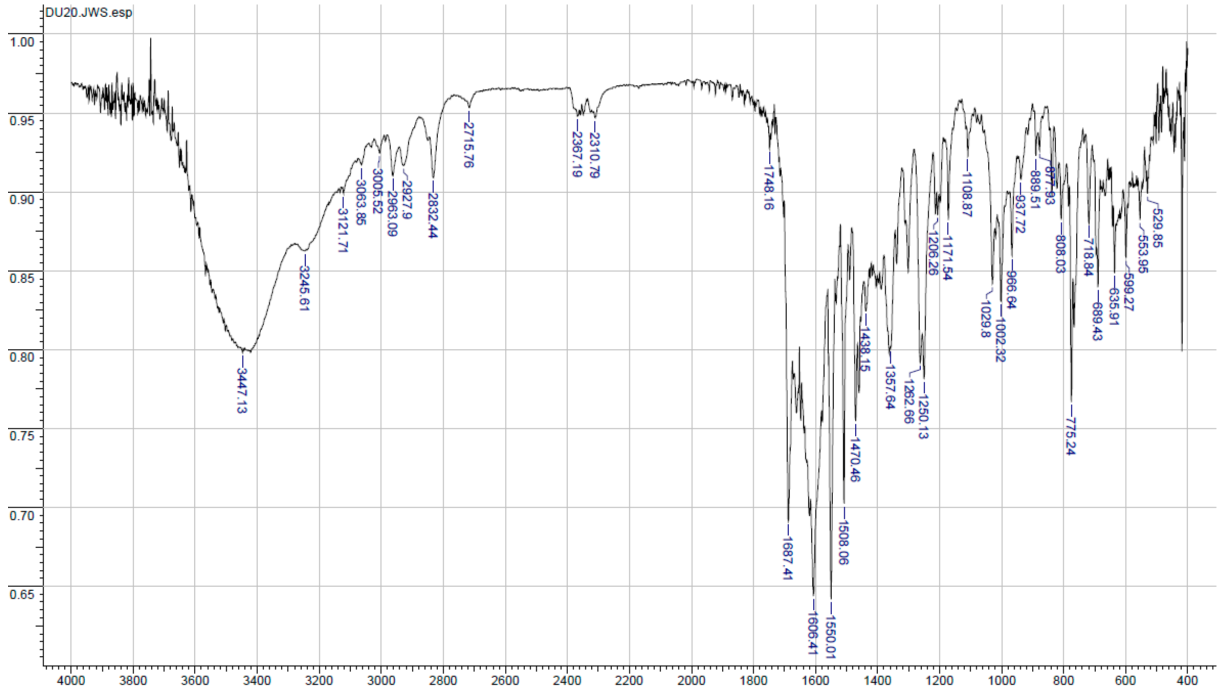

Figure S25. The IR spectrum for the compound **4j**.

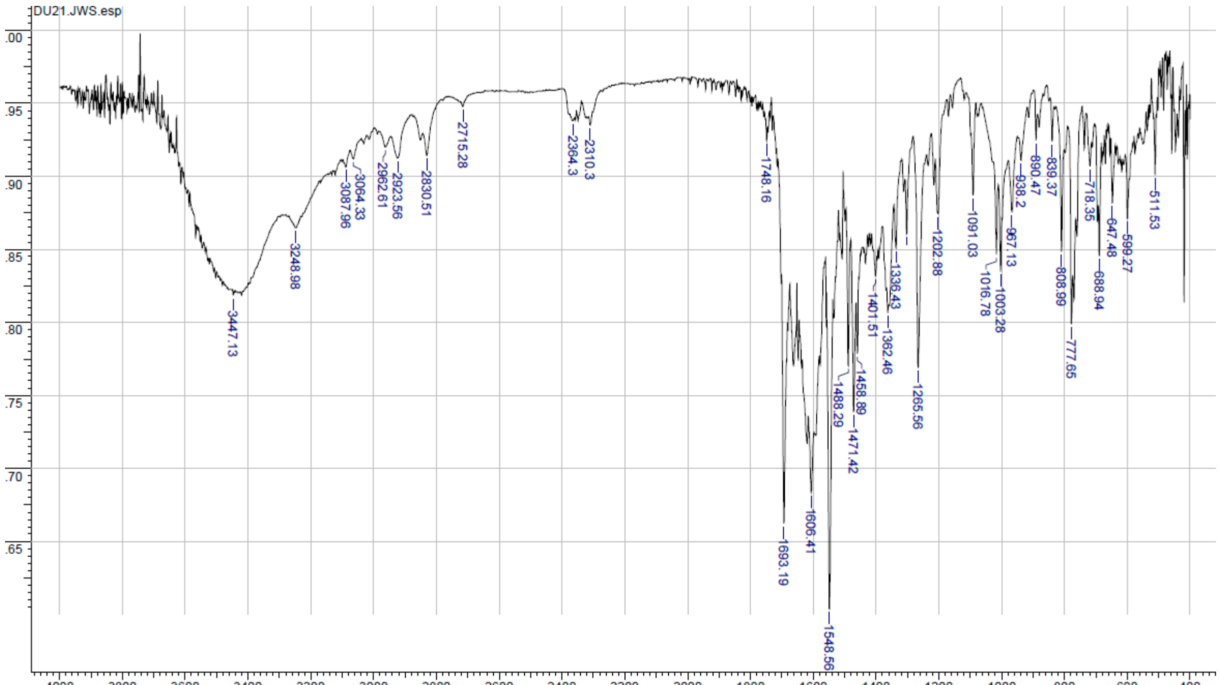

Figure S26. The IR spectrum for the compound **4k**.

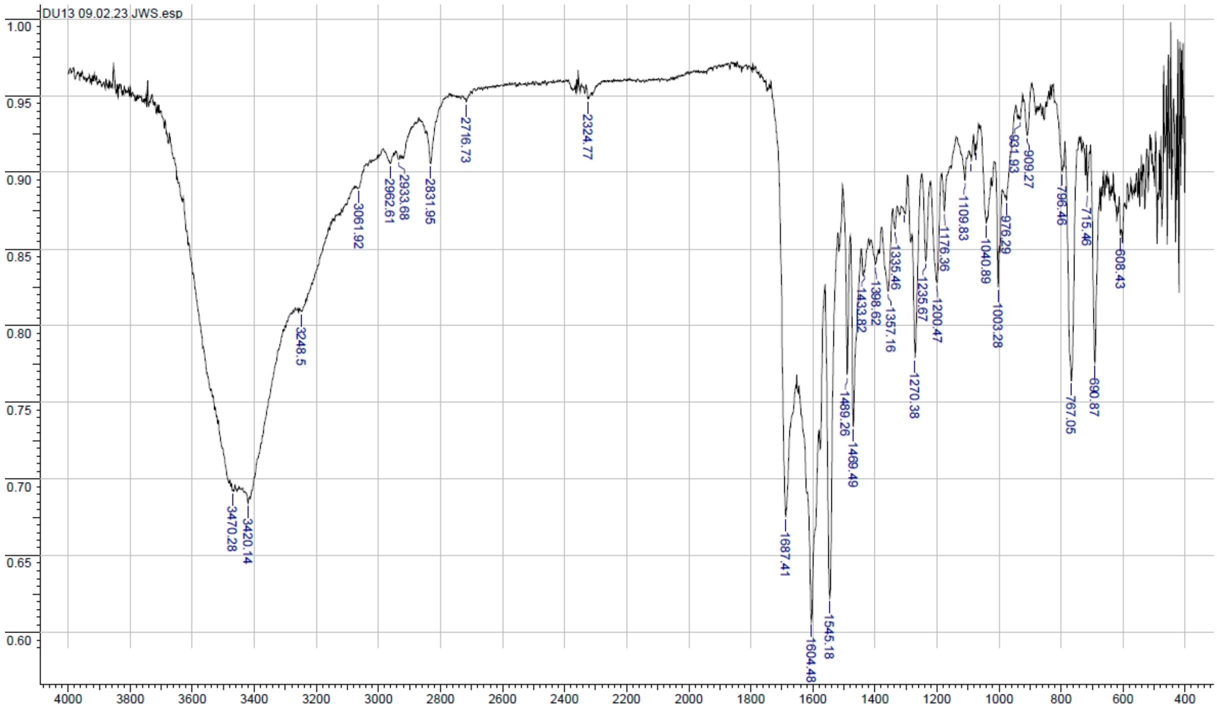

Figure S27. The IR spectrum for the compound **4l**.

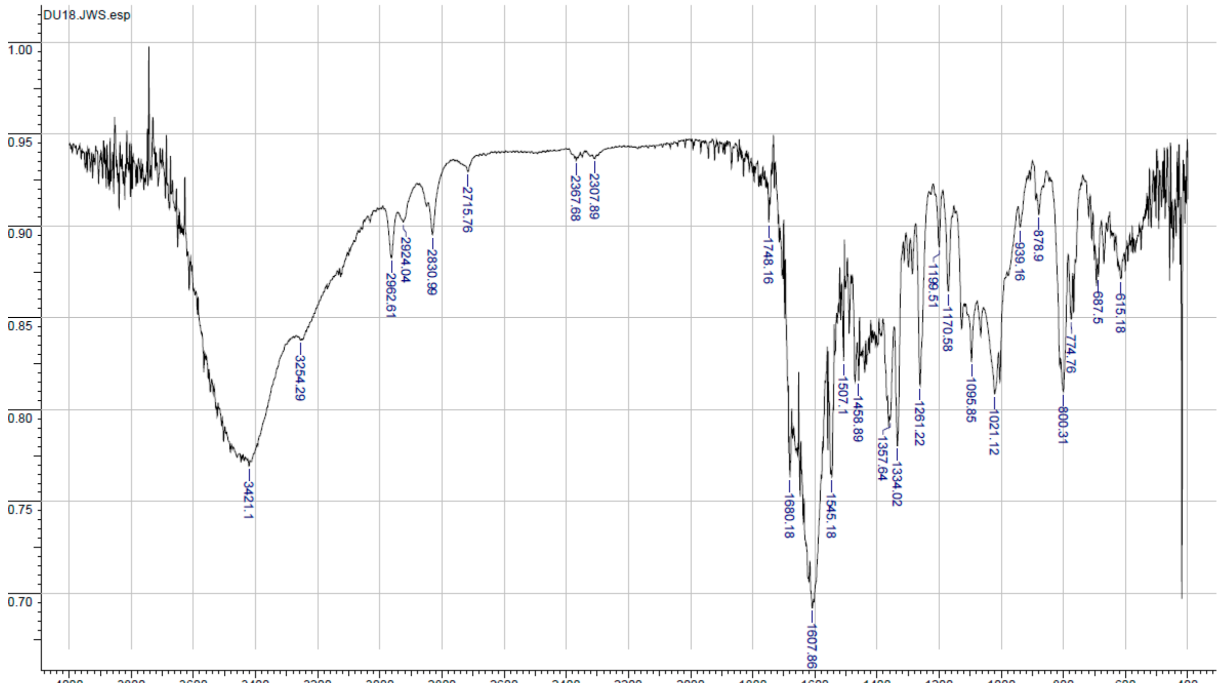

Figure S28. The IR spectrum for the compound **4m**.

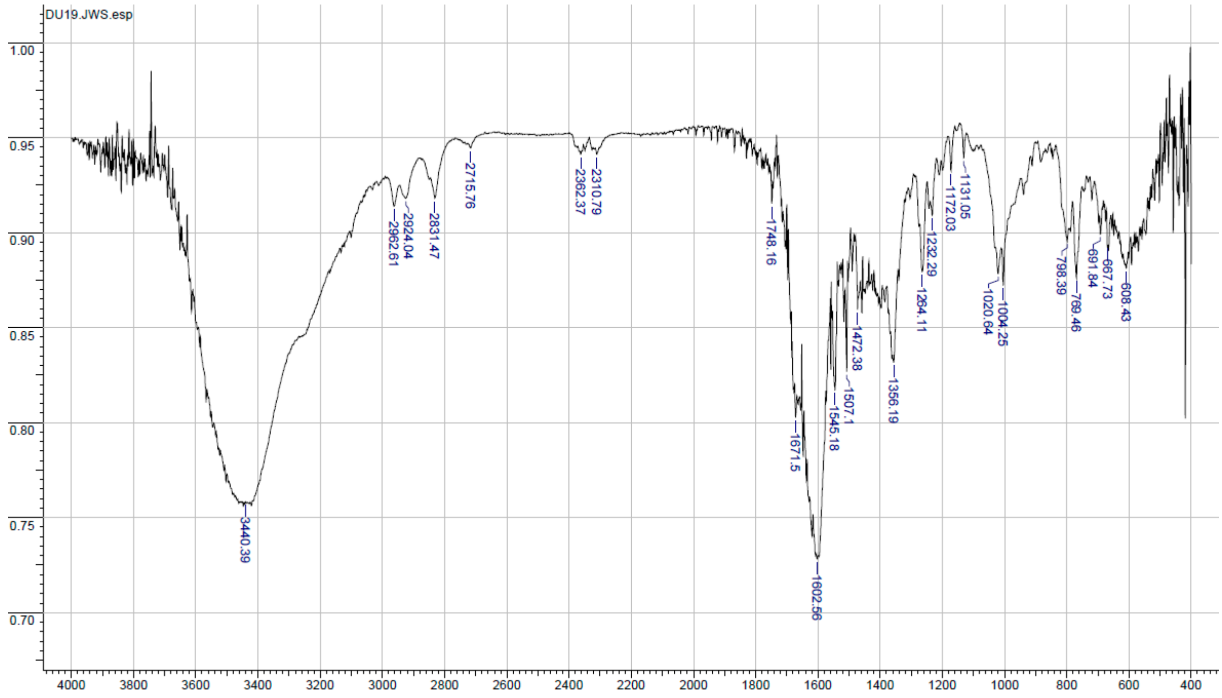

Figure S29. The IR spectrum for the compound **4n**.

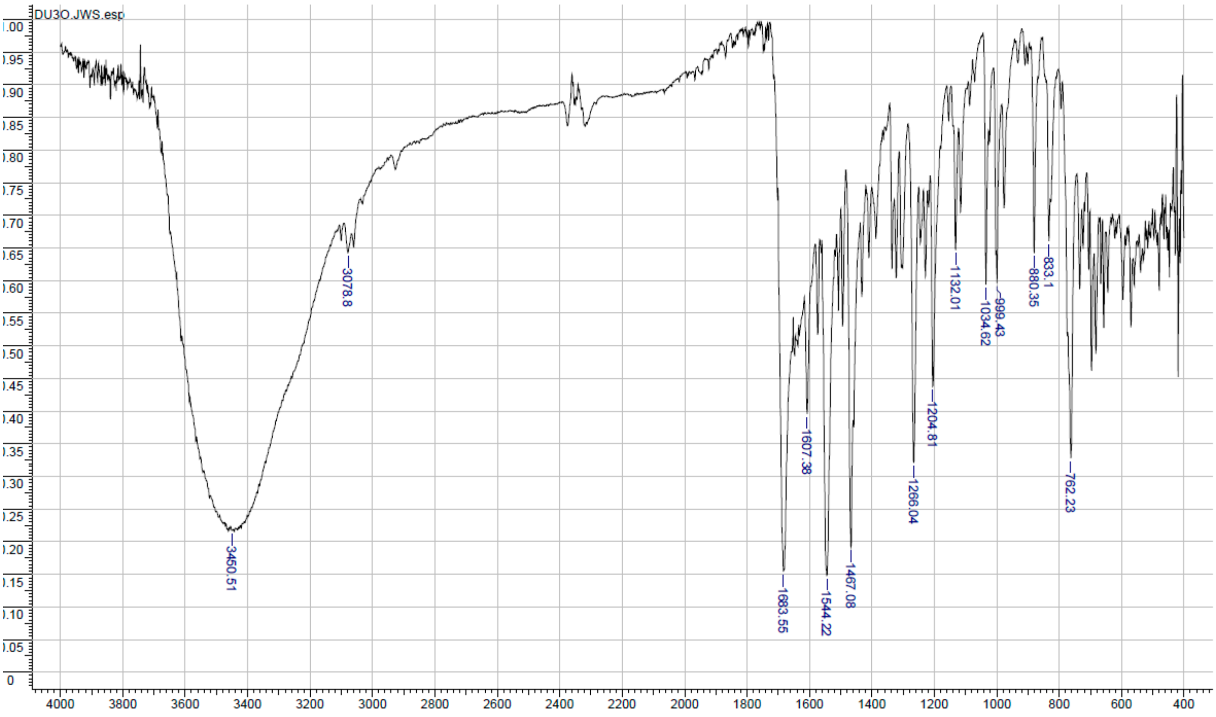

Figure S30. The IR spectrum for the compound **4o**.

1.2. MS Spectra

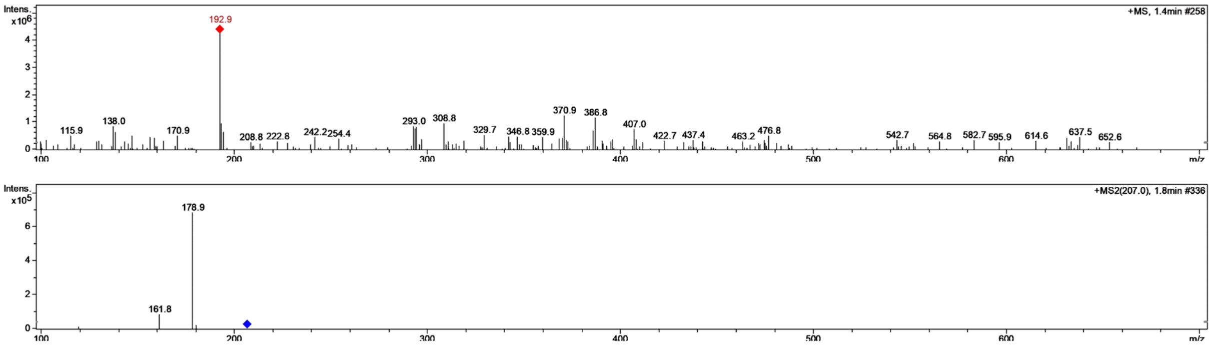

Figure S31. The MS spectrum for the compound **3a**.

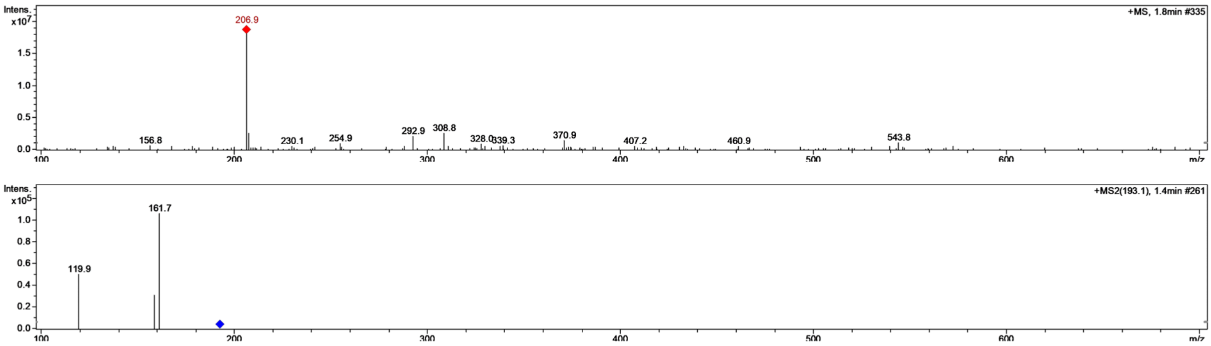

Figure S32. The MS spectrum for the compound **3b**.

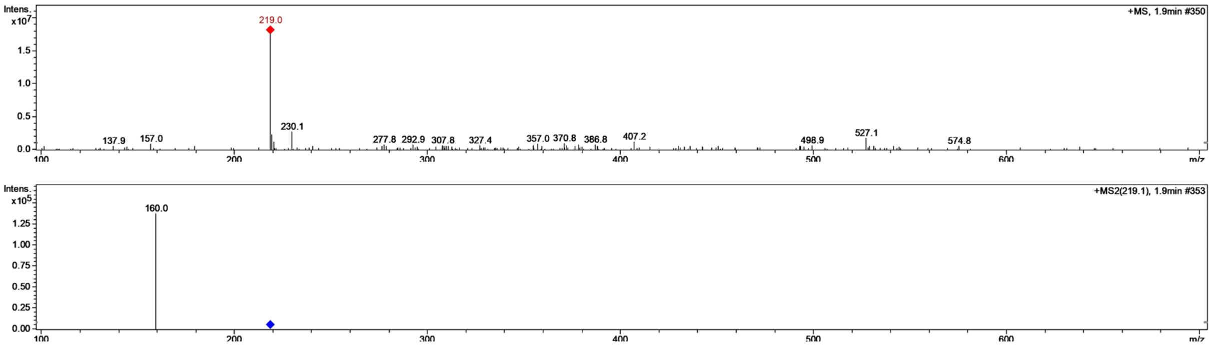

Figure S33. The MS spectrum for the compound **3c**.

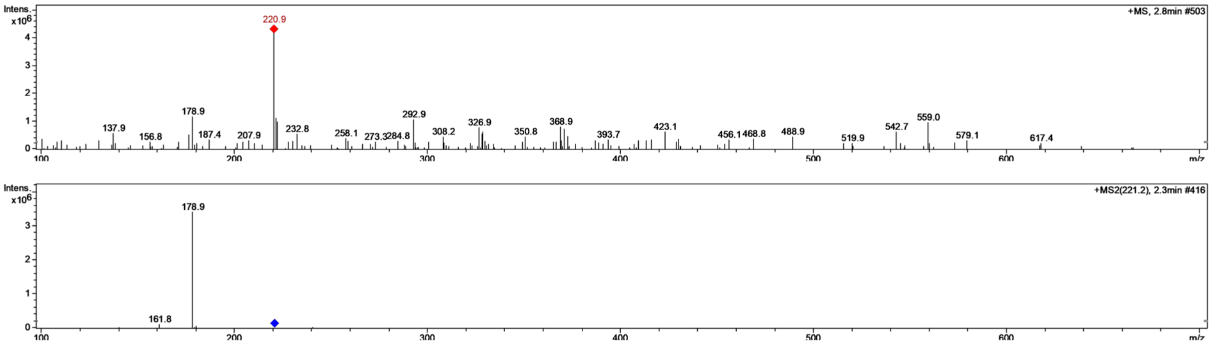

Figure S34. The MS spectrum for the compound **3d**.

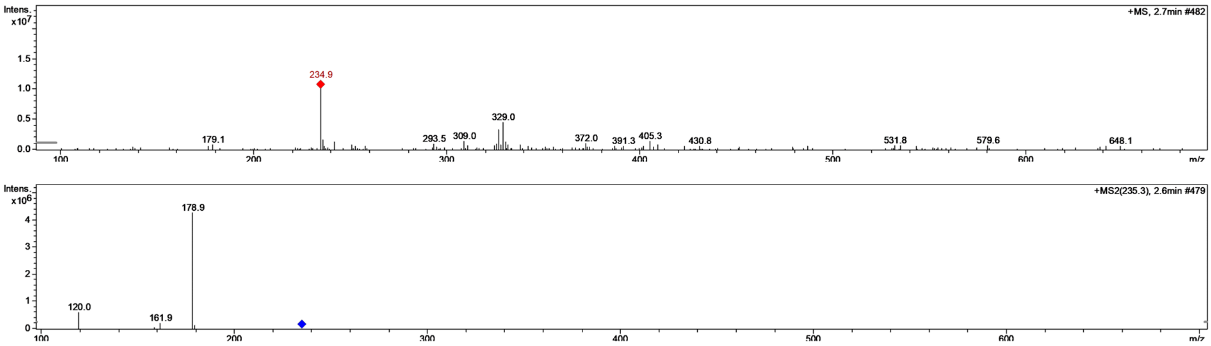

Figure S35. The MS spectrum for the compound **3e**.

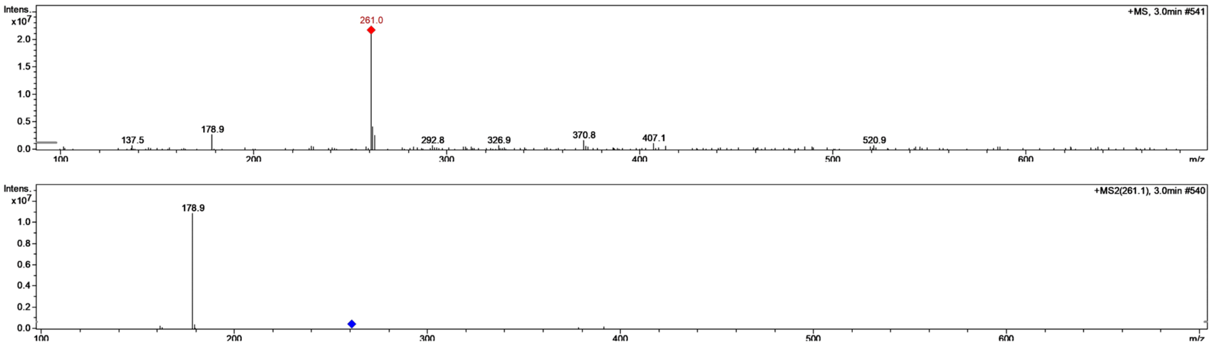

Figure S36. The MS spectrum for the compound **3f**.

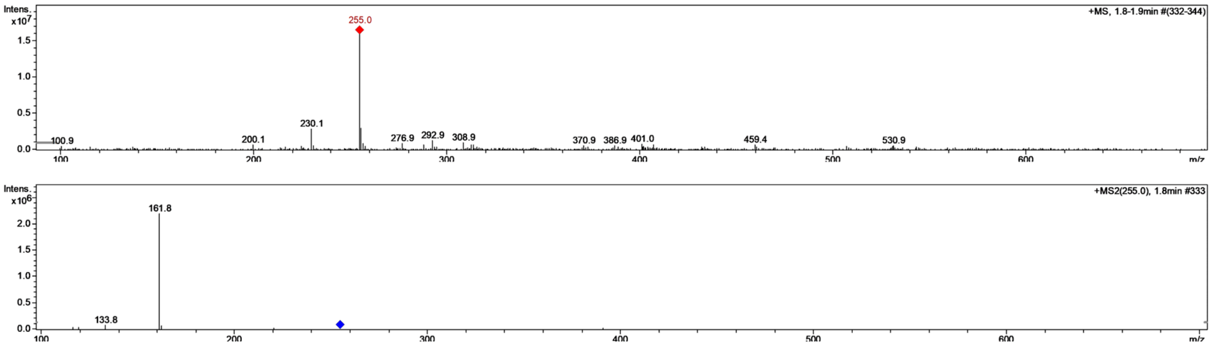

Figure S37. The MS spectrum for the compound **3g**.

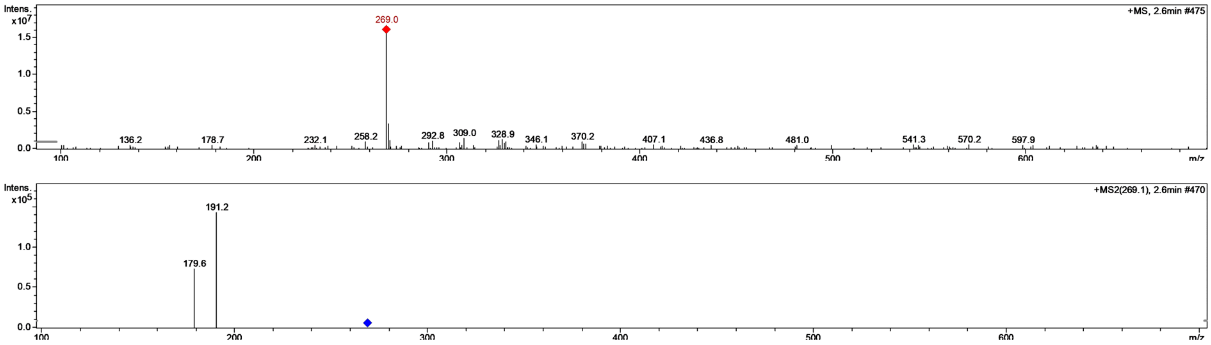

Figure S38. The MS spectrum for the compound **3h**.

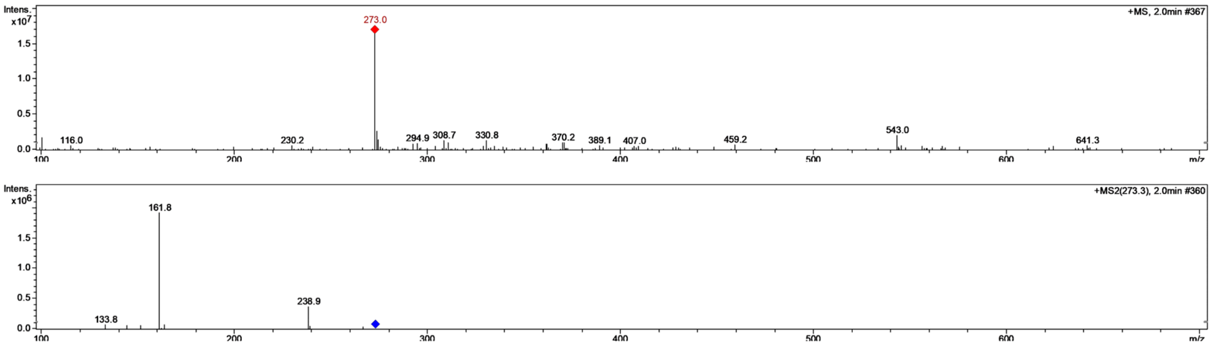

Figure S39. The MS spectrum for the compound **3i**.

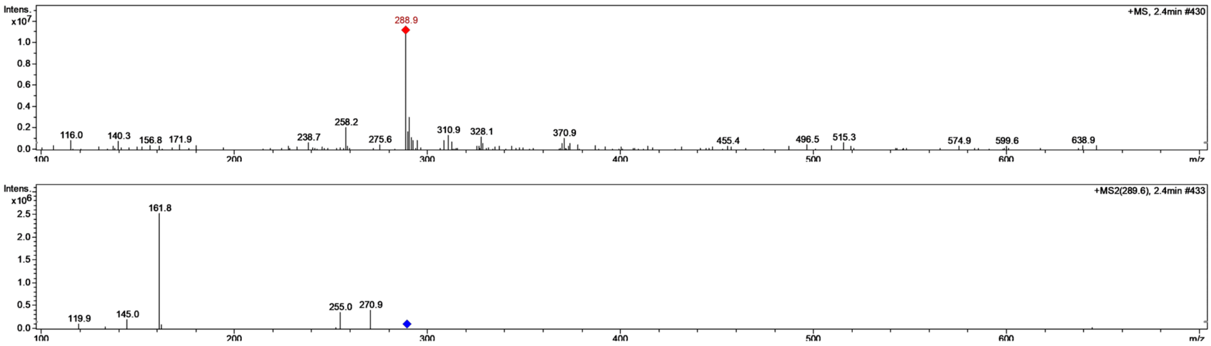

Figure S40. The MS spectrum for the compound **3j**.

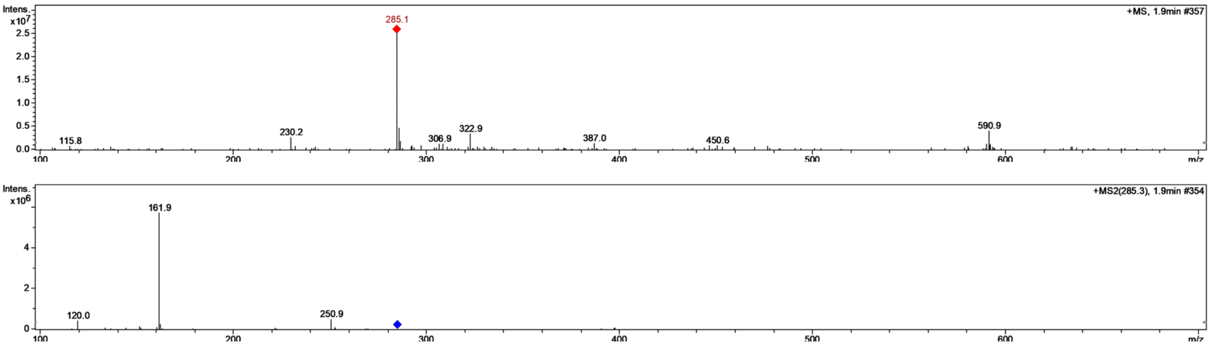

Figure S41. The MS spectrum for the compound **3k**.

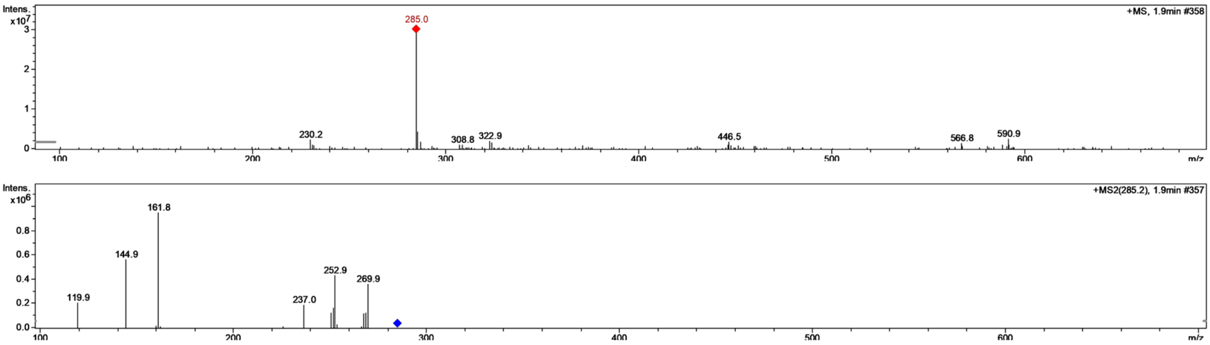

Figure S42. The MS spectrum for the compound **3l**.

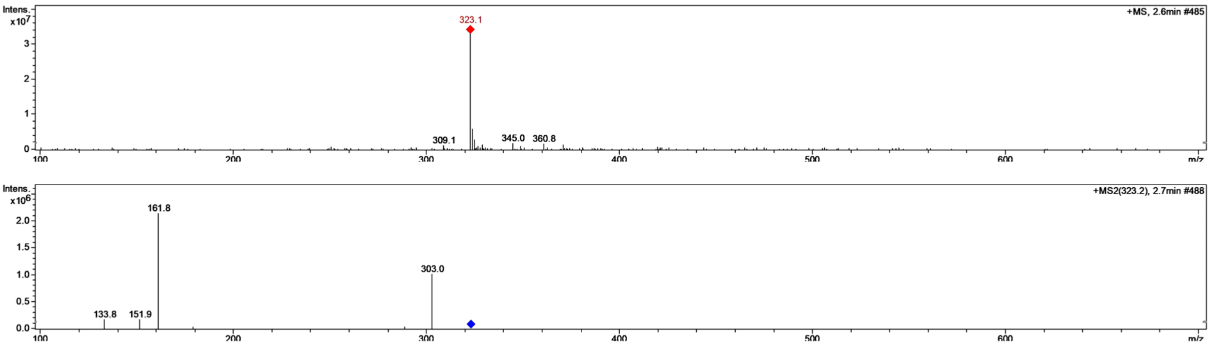

Figure S43. The MS spectrum for the compound **3m**.

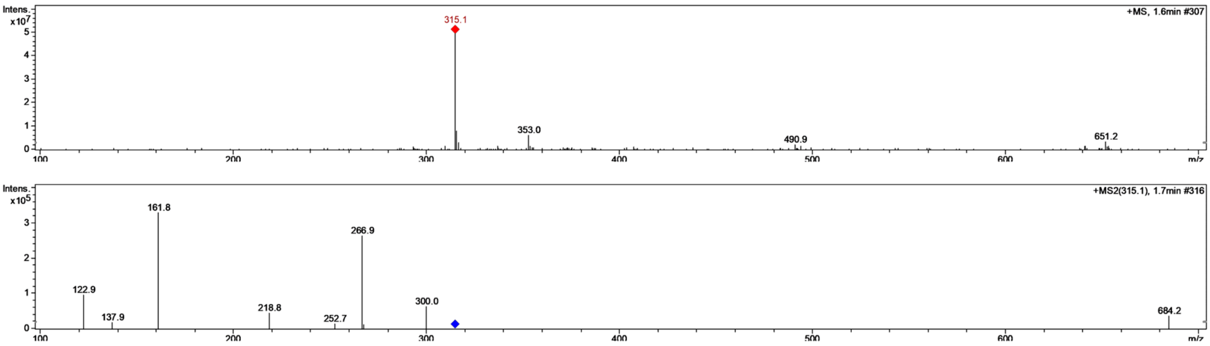

Figure S44. The MS spectrum for the compound **3n**.

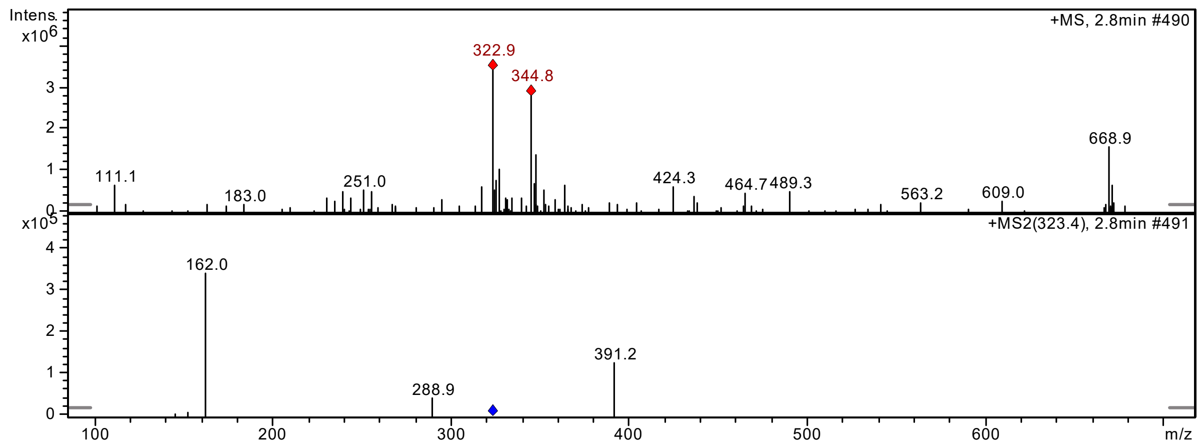

Figure S45. The MS spectrum for the compound **3o**.

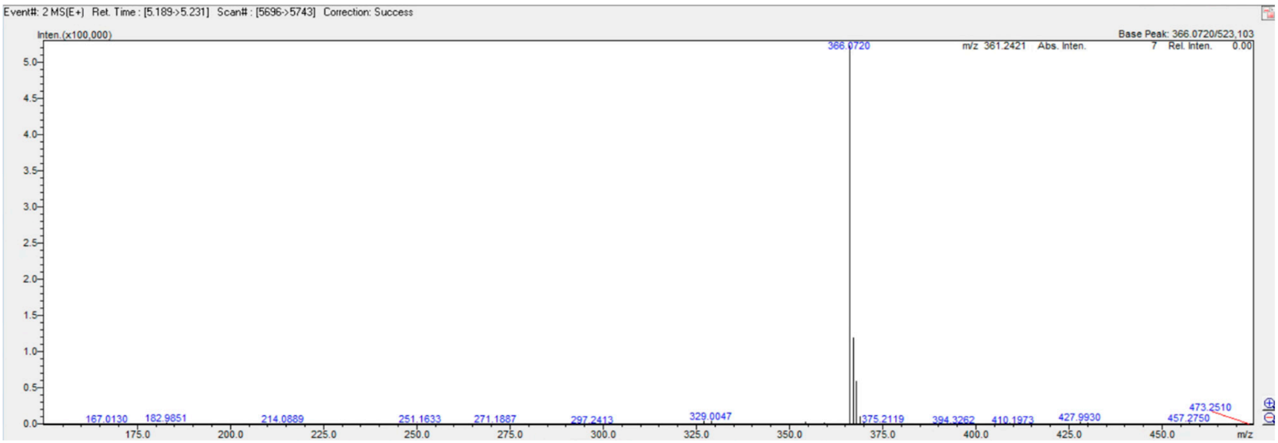

Figure S46. The MS spectrum for the compound **4a**.

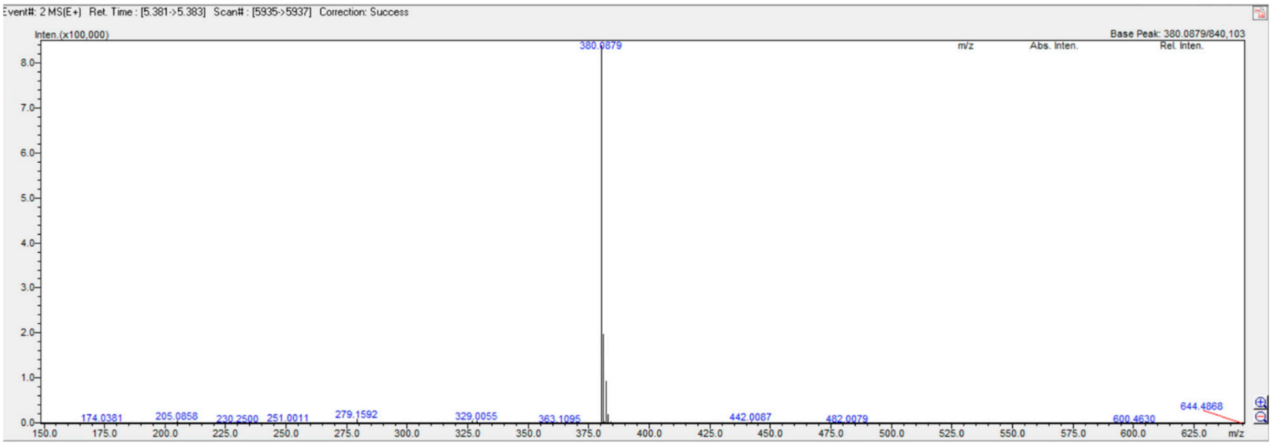

Figure S47. The MS spectrum for the compound **4b**.

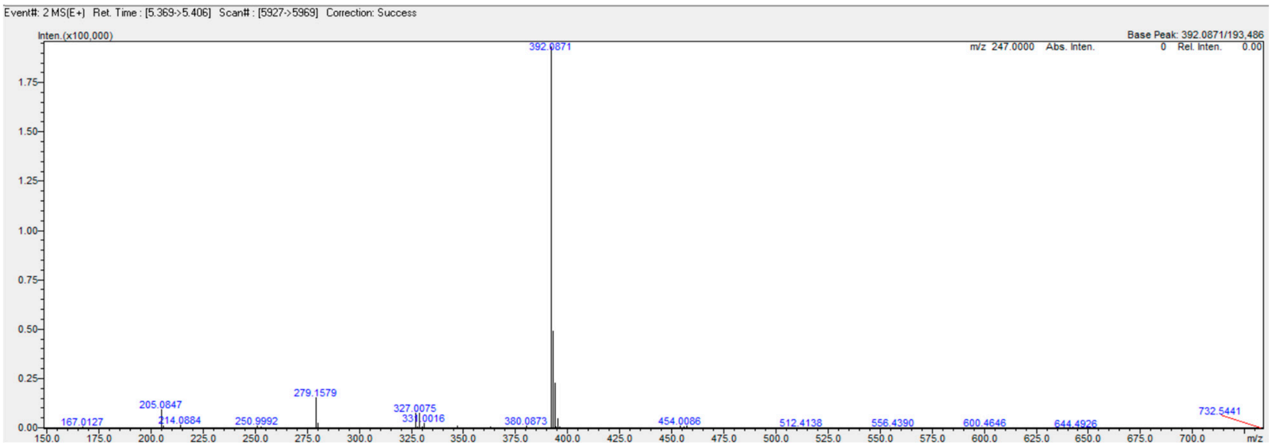

Figure S48. The MS spectrum for the compound **4c**.

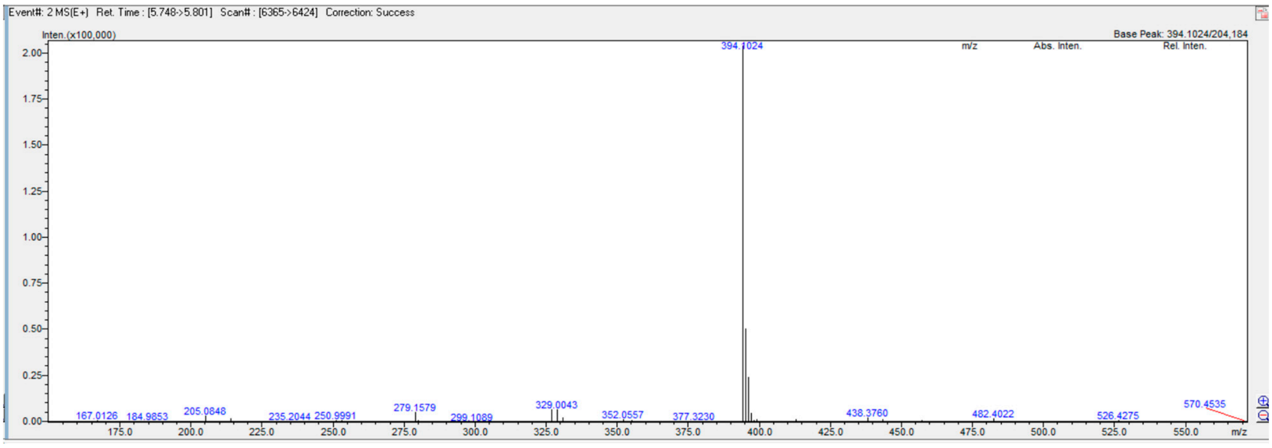

Figure S49. The MS spectrum for the compound **4d**.

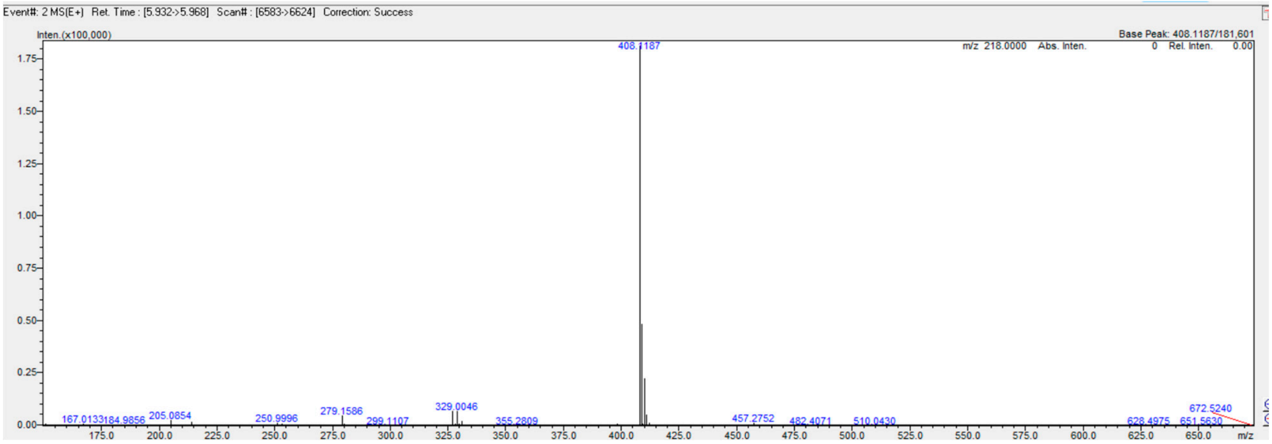

Figure S50. The MS spectrum for the compound **4e**.

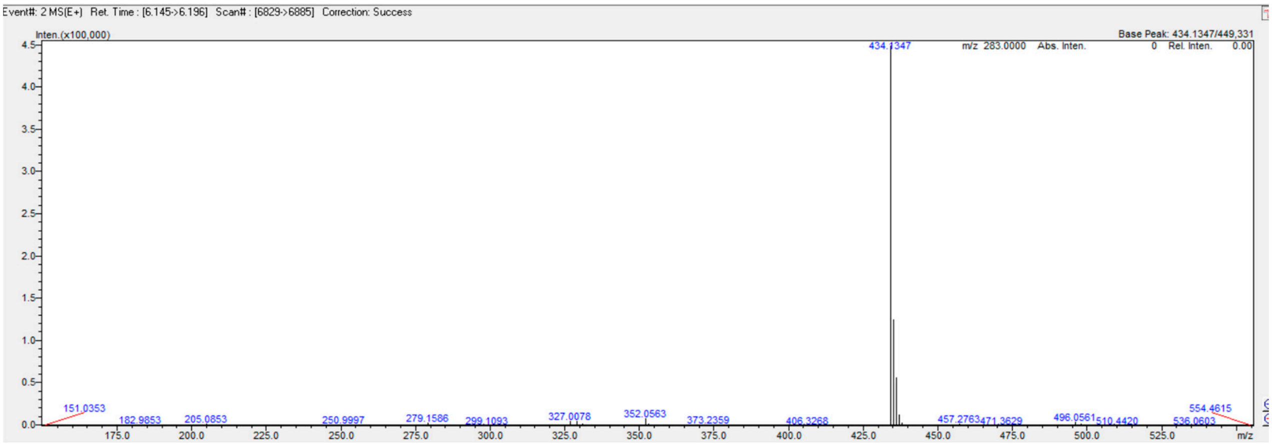

Figure S51. The MS spectrum for the compound **4f**.

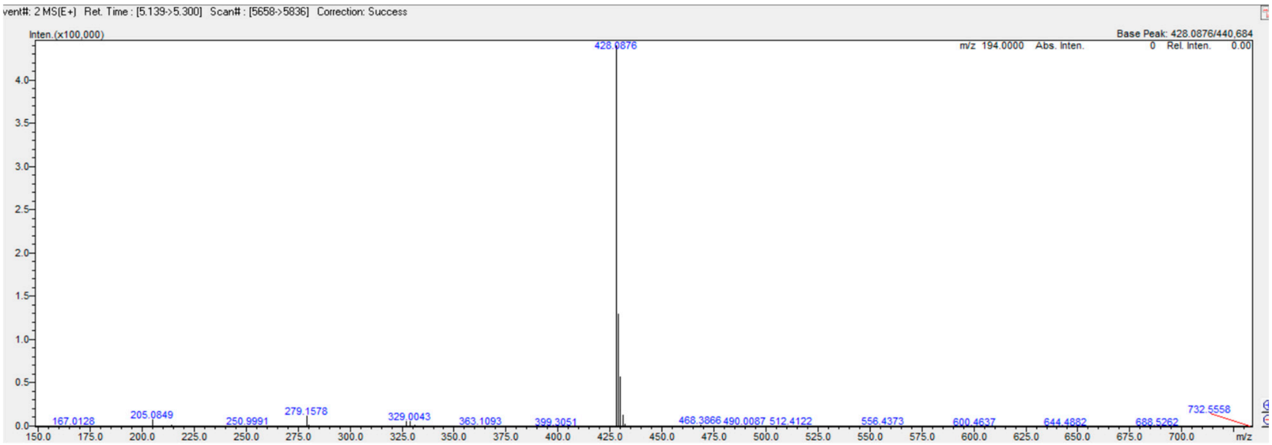

Figure S52. The MS spectrum for the compound **4g**.

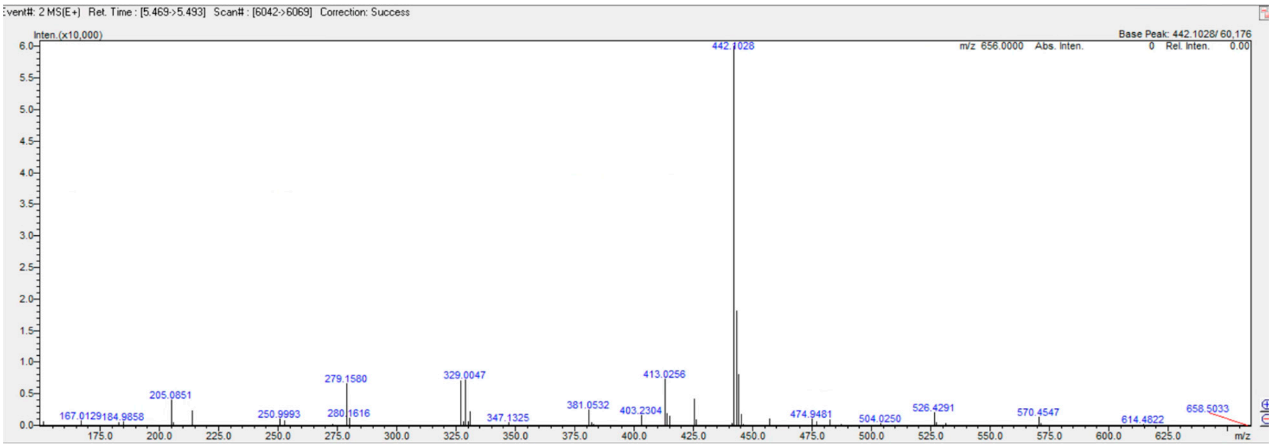

Figure S53. The MS spectrum for the compound **4h**.

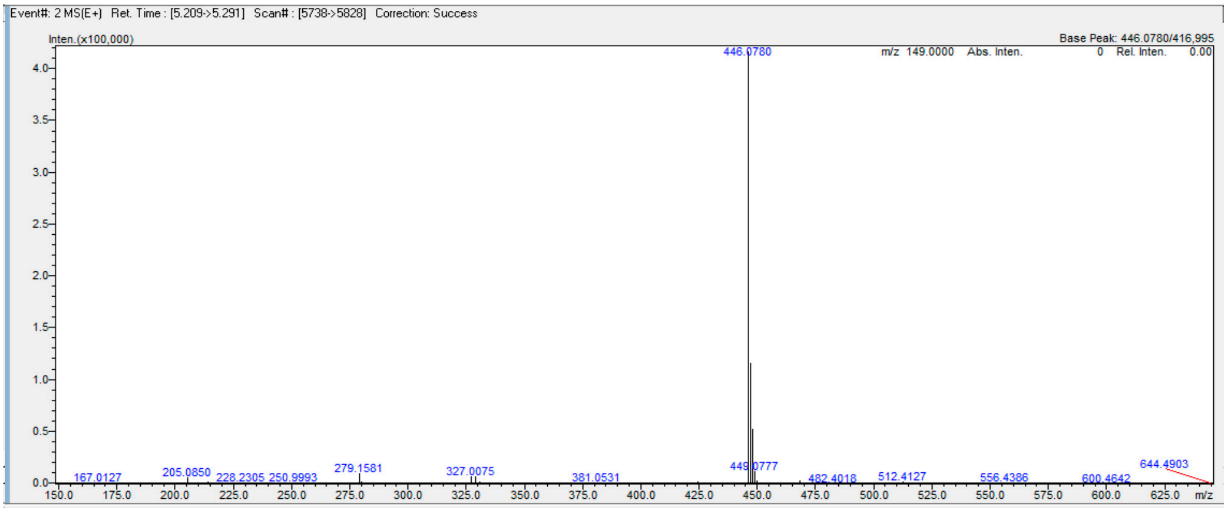

Figure S54. The MS spectrum for the compound **4i**.

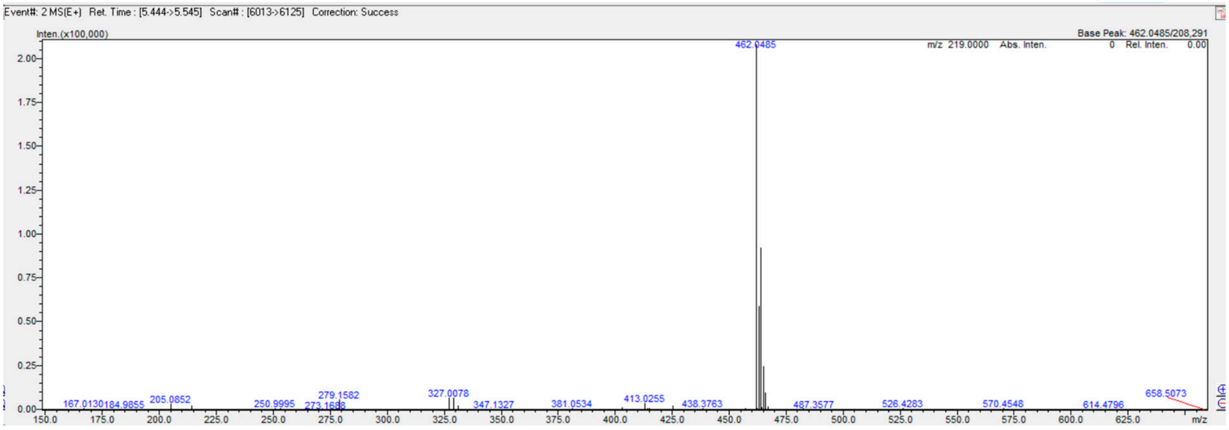

Figure S55. The MS spectrum for the compound **4j**.

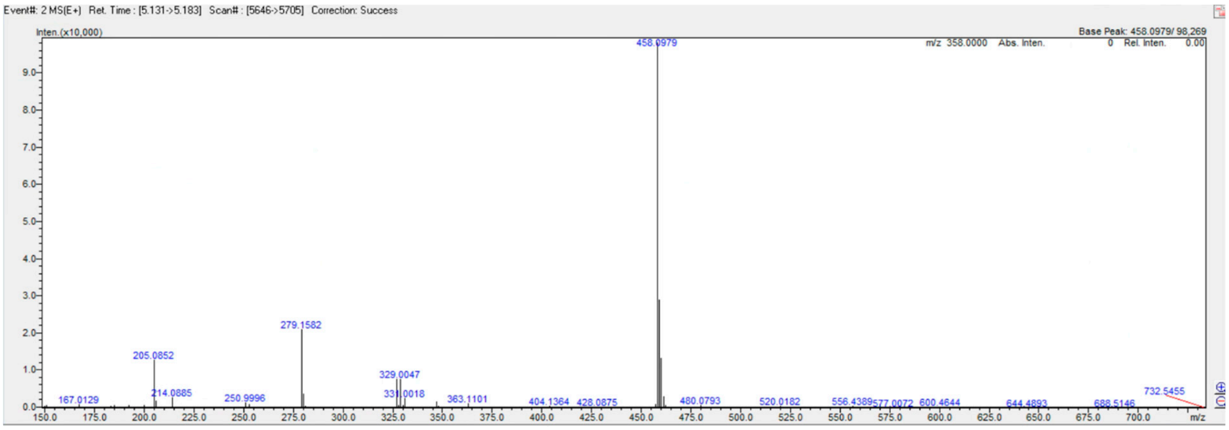

Figure S56. The MS spectrum for the compound **4k**.

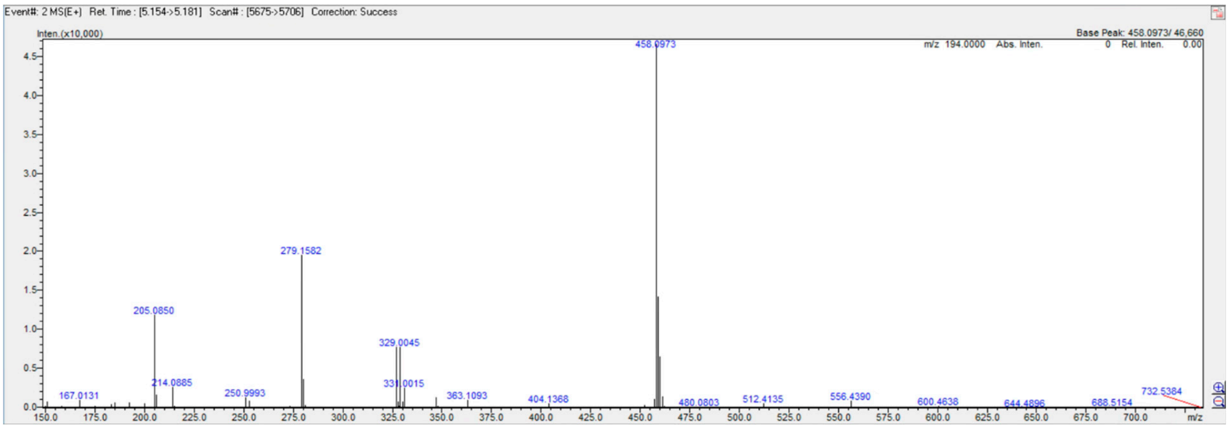

Figure S57. The MS spectrum for the compound **4l**.

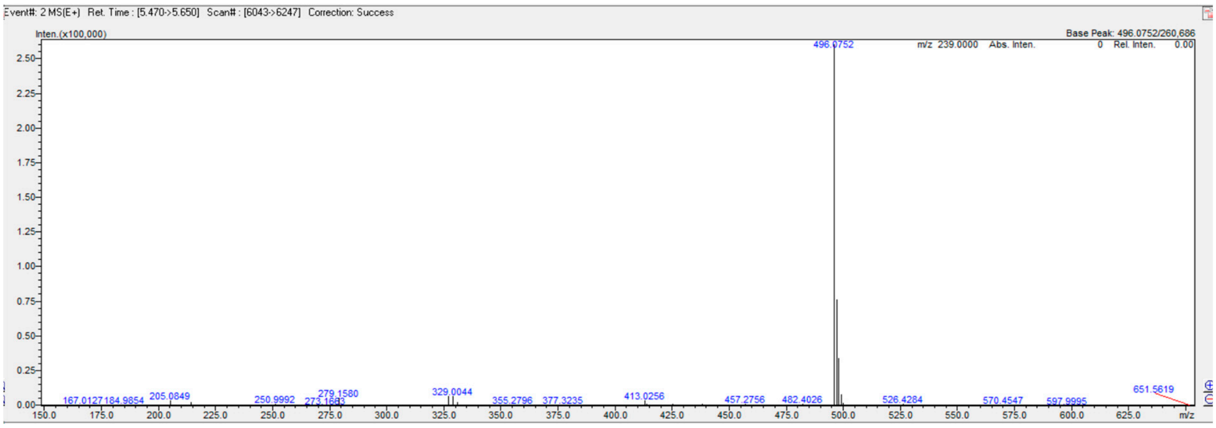

Figure S58. The MS spectrum for the compound **4m**.

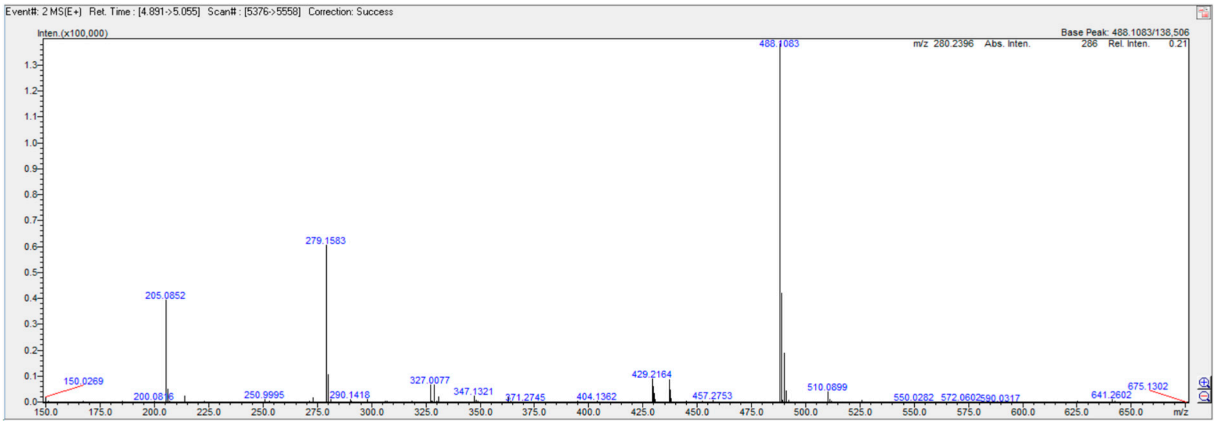

Figure S59. The MS spectrum for the compound **4n**.

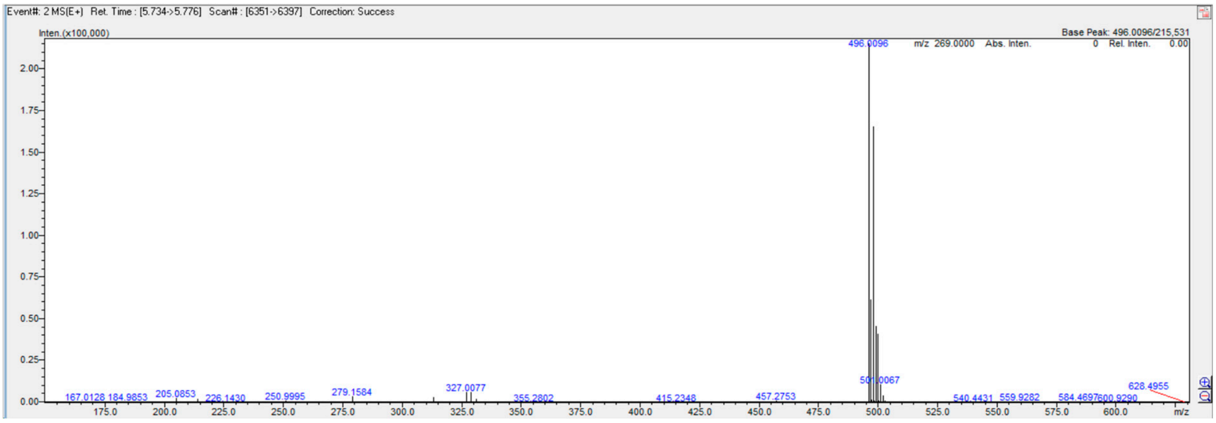

Figure S60. The MS spectrum for the compound **4o**.

1.3. ^{1}H-NMR Spectra

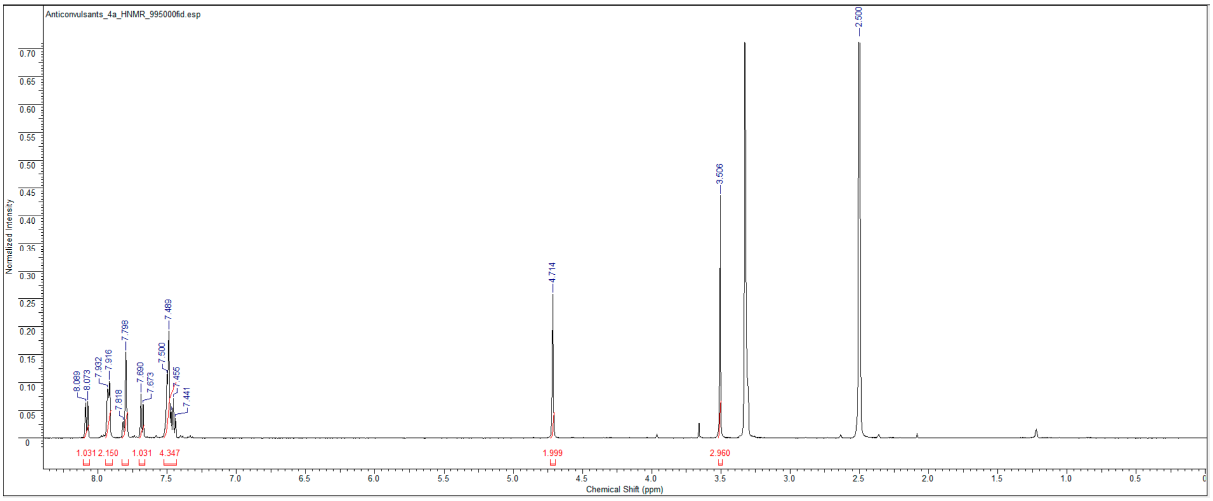

Figure S61. The ^{1}H-NMR spectrum for the compound **4a**.

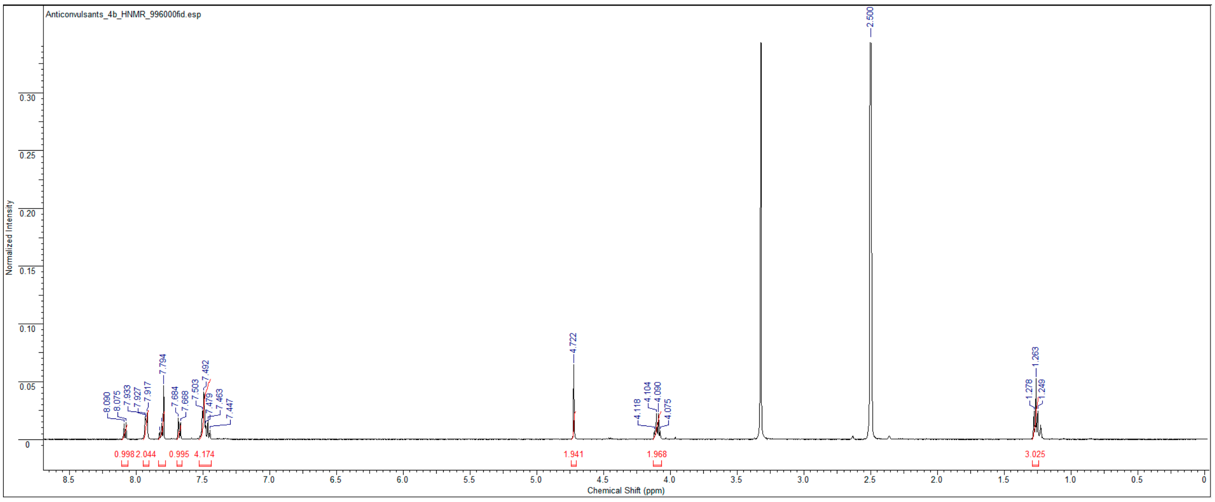

Figure S62. The ^{1}H-NMR spectrum for the compound **4b**.

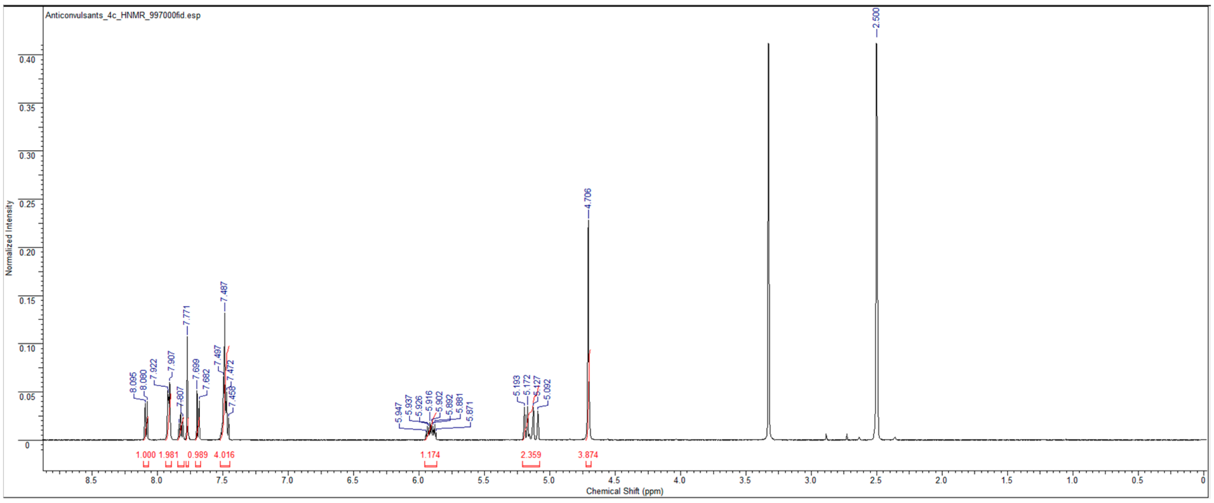

Figure S63. The ^{1}H-NMR spectrum for the compound **4c**.

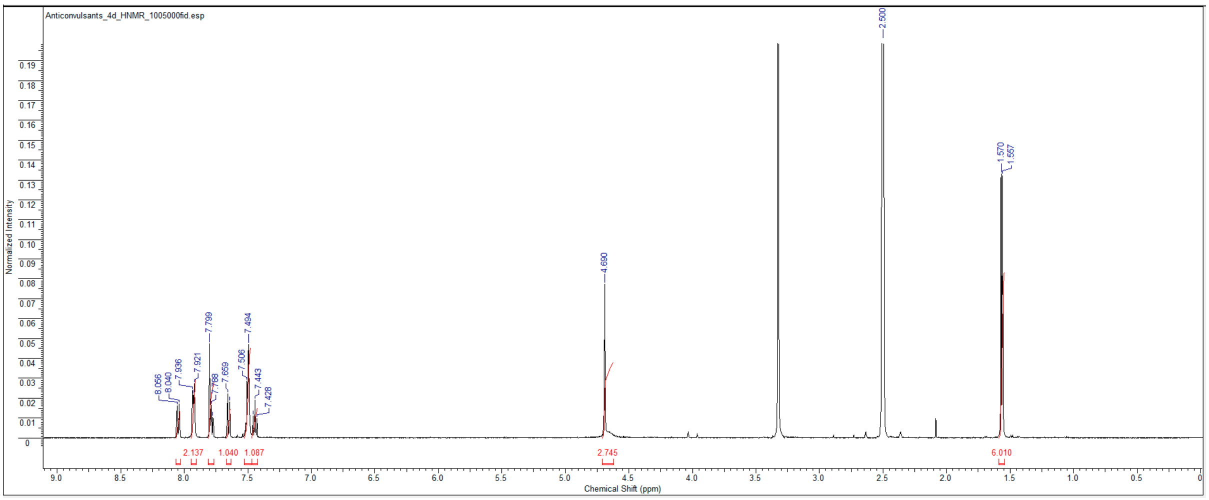

Figure S64. The ^{1}H-NMR spectrum for the compound **4d**.

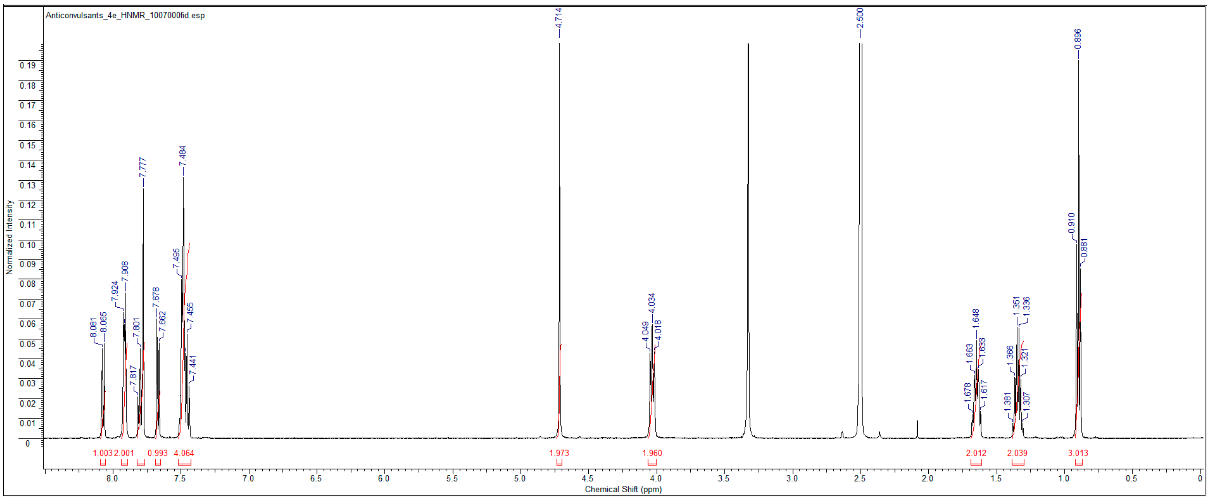

Figure S65. The ^{1}H-NMR spectrum for the compound **4e**.

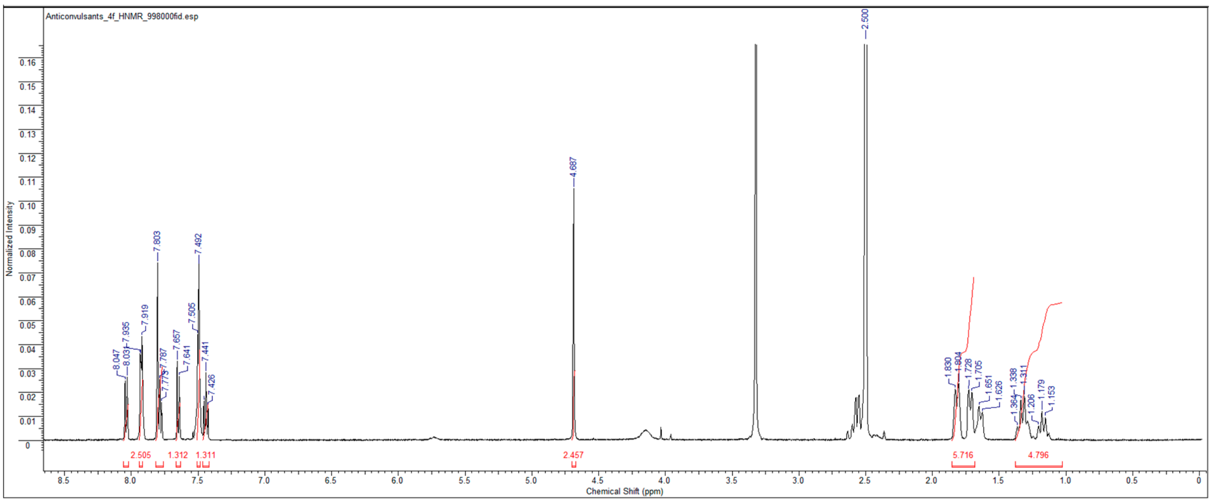

Figure S66. The ^{1}H-NMR spectrum for the compound **4f**.

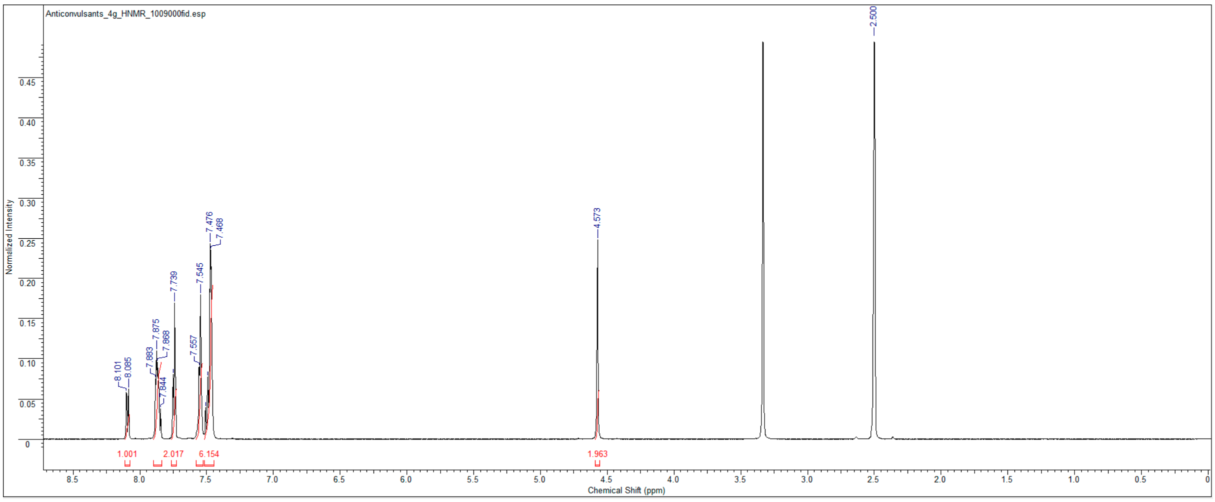

Figure S67. The ^{1}H-NMR spectrum for the compound **4g**.

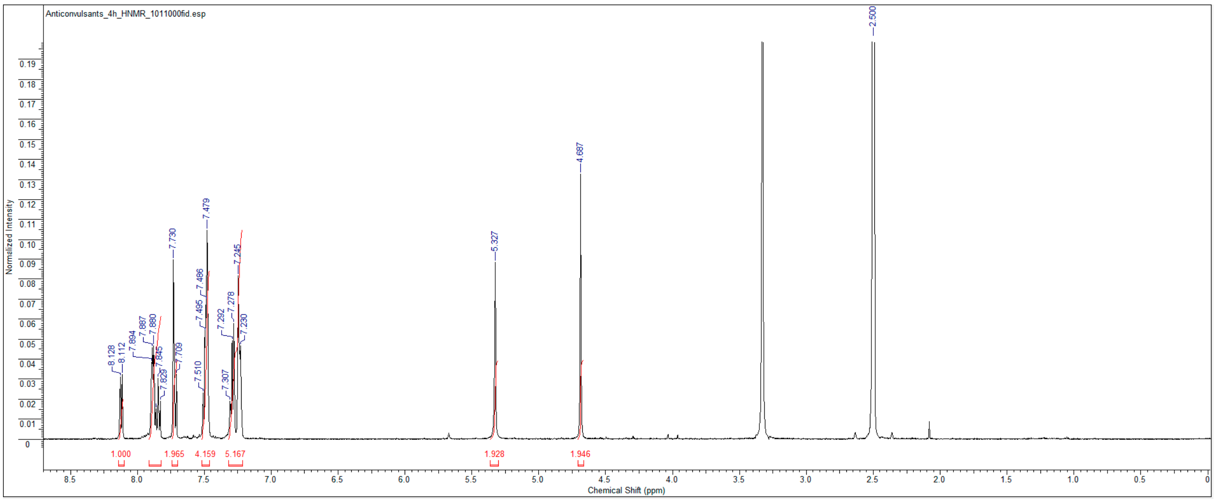

Figure S68. The ^{1}H-NMR spectrum for the compound **4h**.

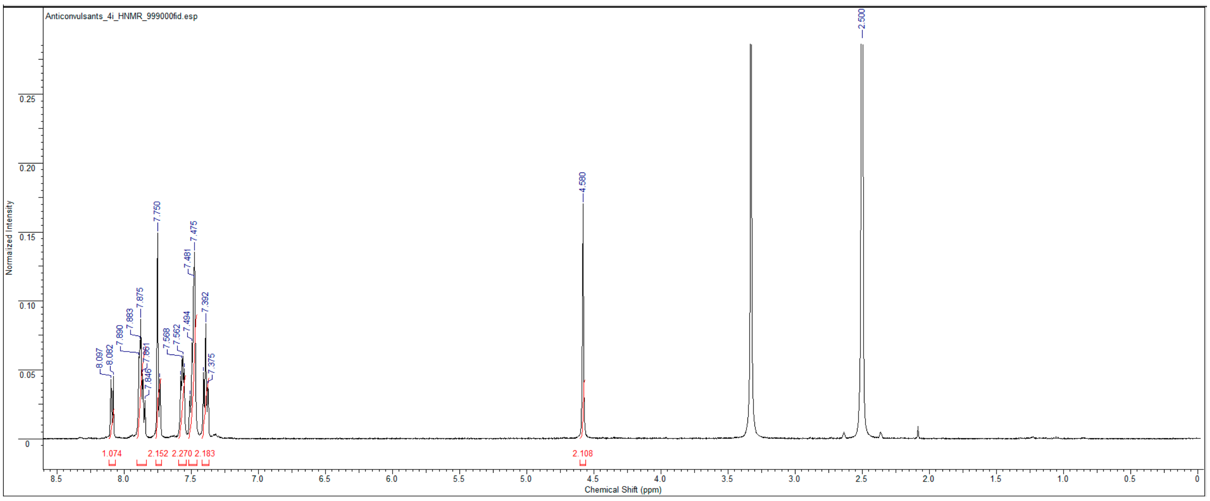

Figure S69. The ¹H-NMR spectrum for the compound **4i**.

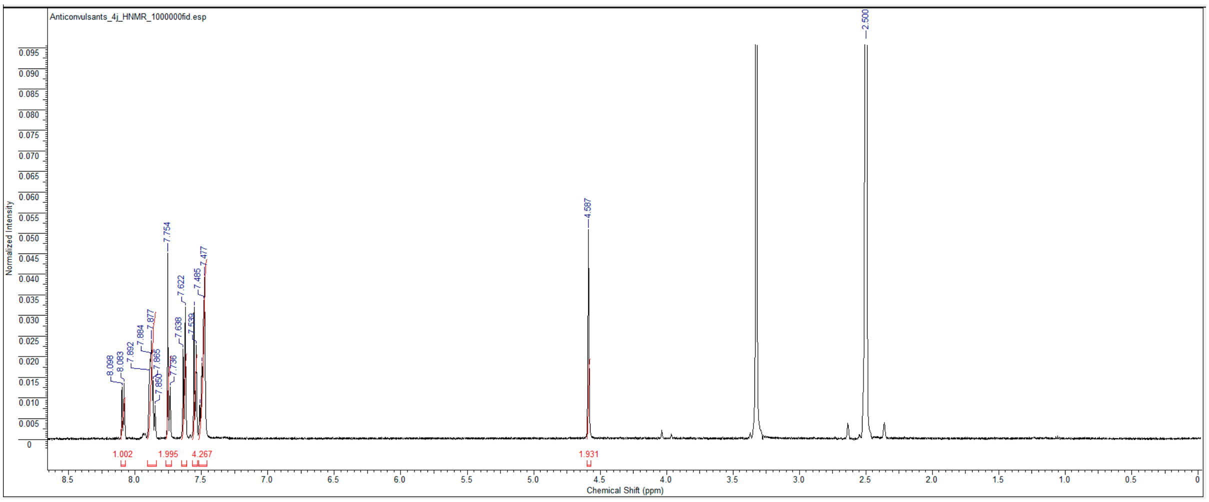

Figure S70. The ¹H-NMR spectrum for the compound **4j**.

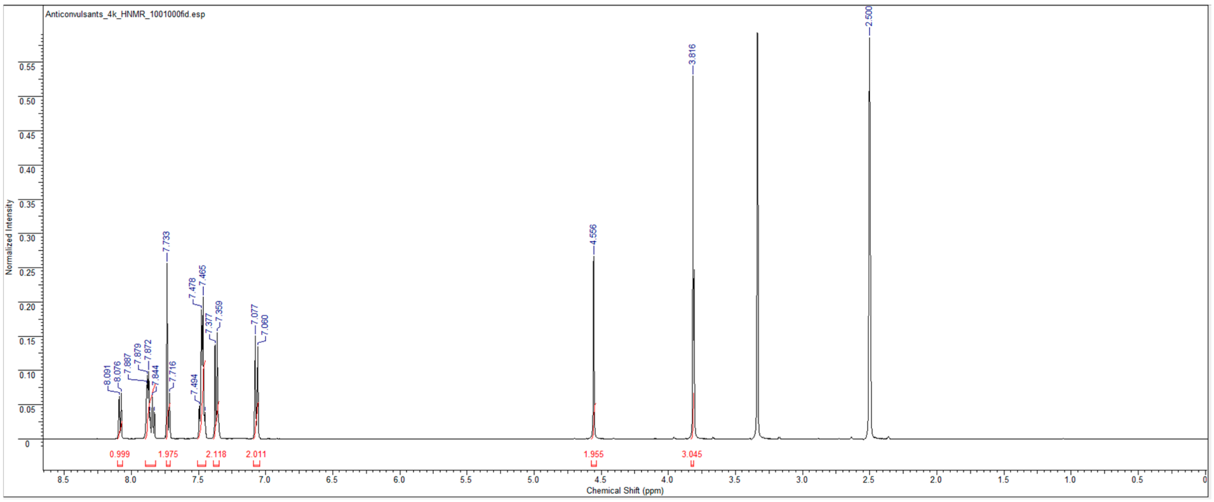

Figure S71. The ¹H-NMR spectrum for the compound **4k**.

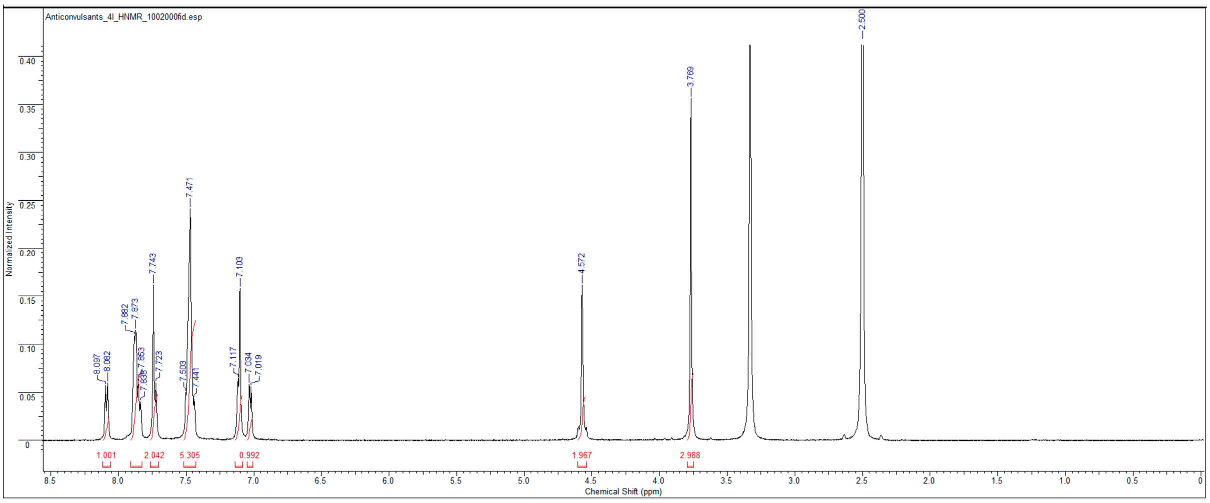

Figure S72. The ^{1}H-NMR spectrum for the compound **4l**.

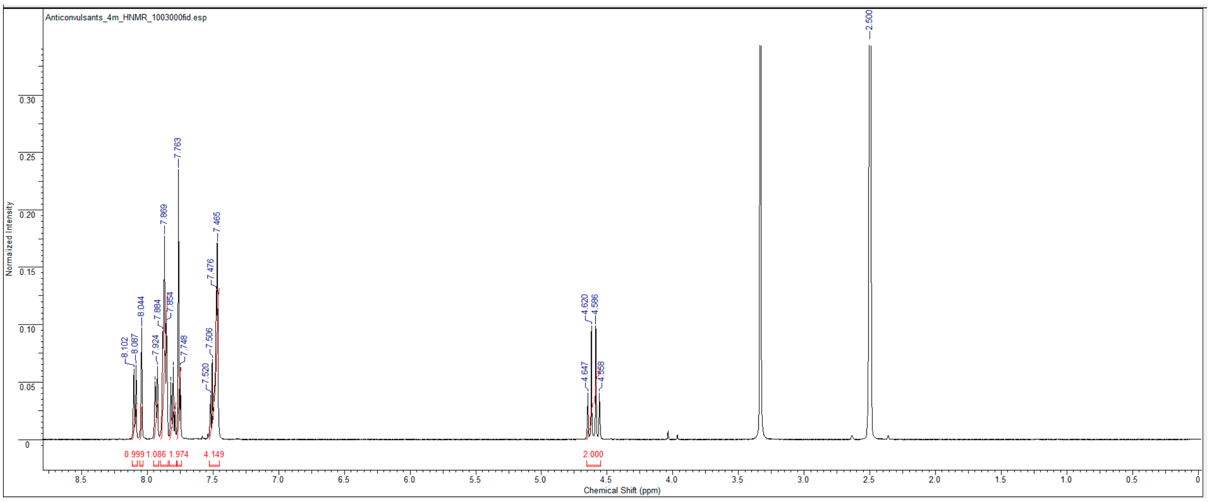

Figure S73. The ^{1}H-NMR spectrum for the compound **4m**.

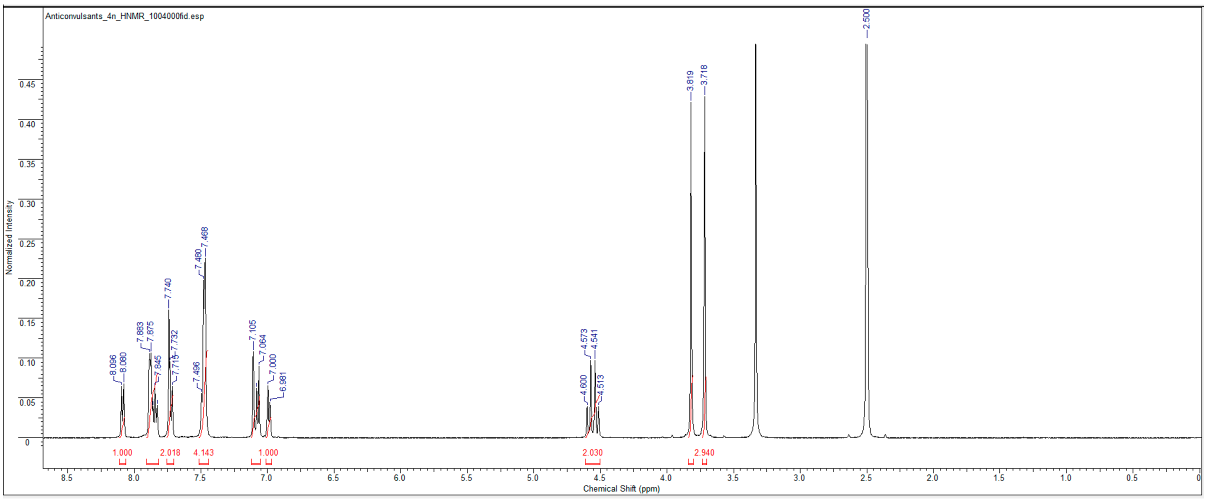

Figure S74. The ^{1}H-NMR spectrum for the compound **4n**.

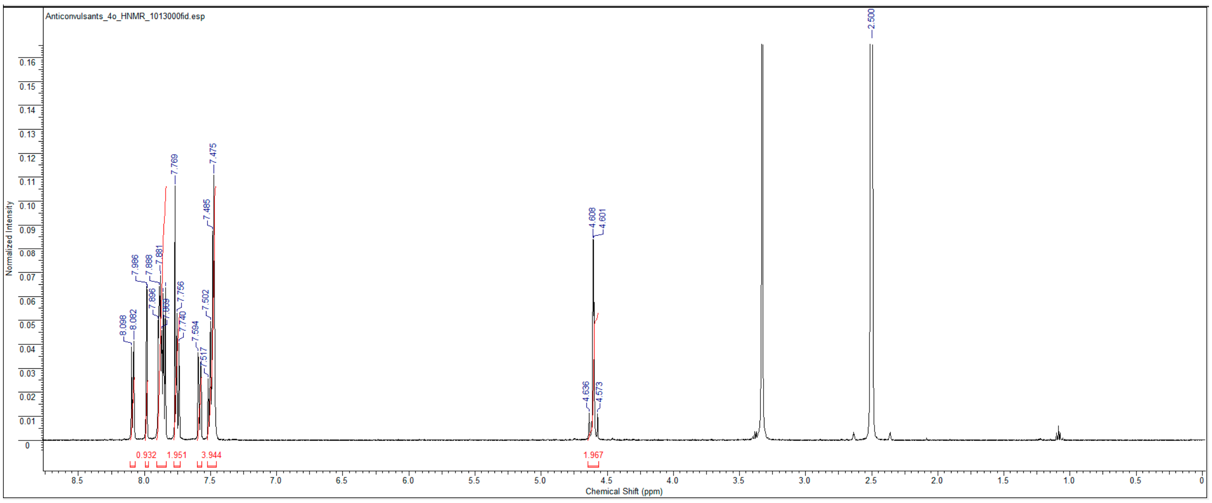

Figure S75. The ^{1}H-NMR spectrum for the compound **4o**.

1.4. ^{13}C-NMR Spectra

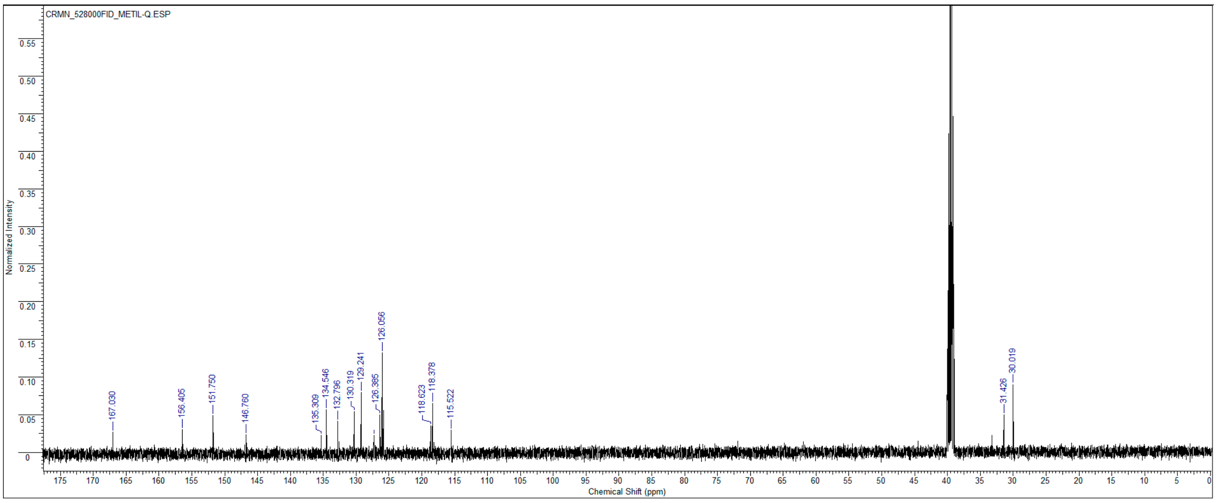

Figure S76. The ^{13}C-NMR spectrum for the compound **4a**.

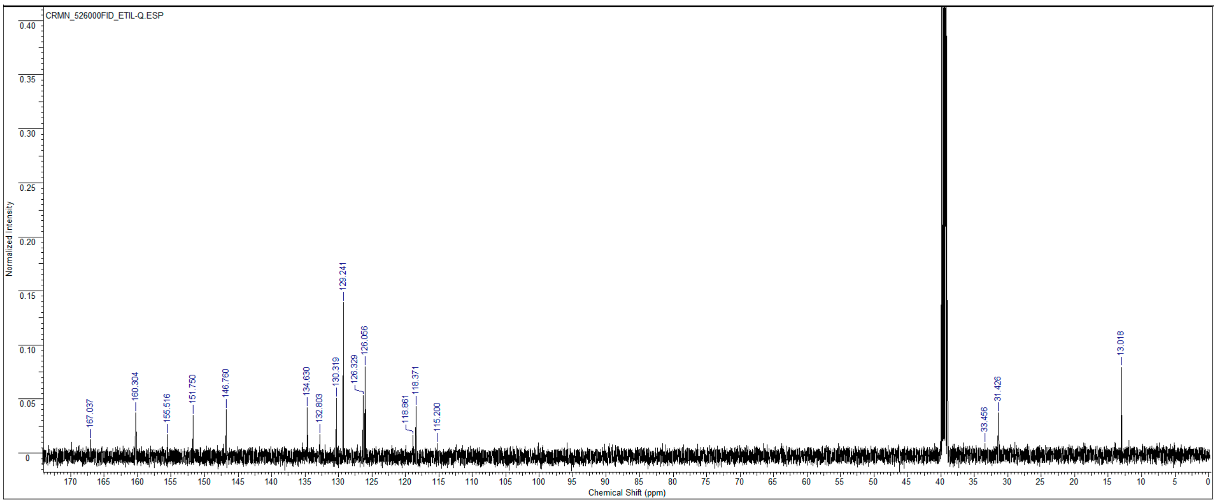

Figure S77. The ^{13}C-NMR spectrum for the compound **4b**.

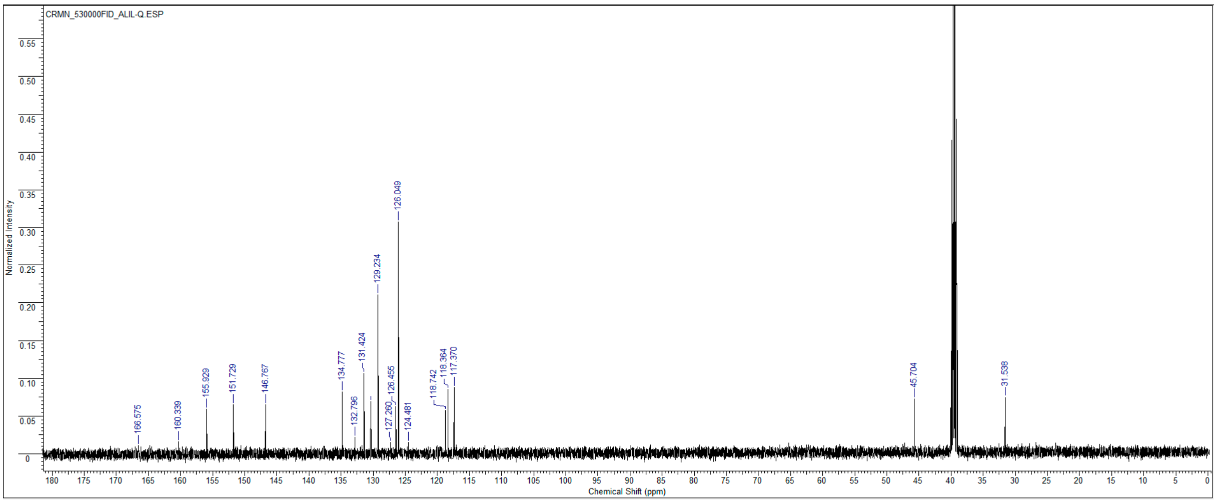

Figure S78. The ^{13}C-NMR spectrum for the compound **4c**.

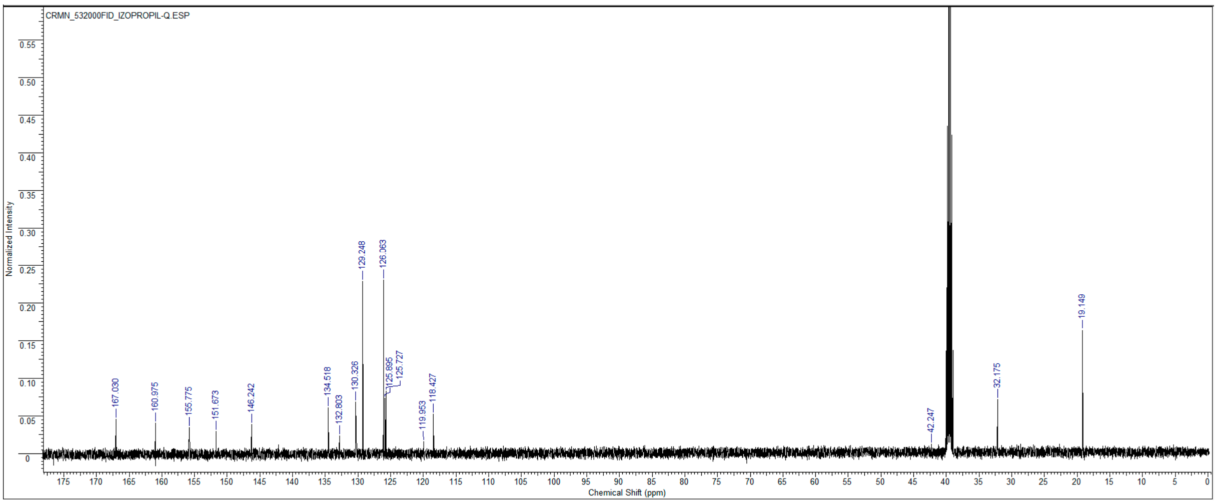

Figure S79. The ^{13}C-NMR spectrum for the compound **4d**.

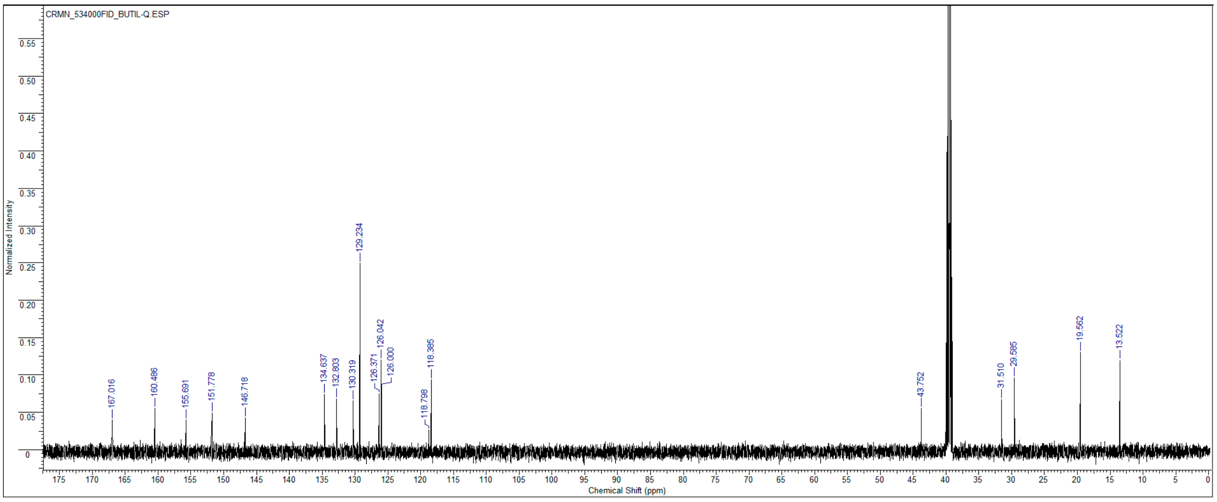

Figure S80. The ^{13}C-NMR spectrum for the compound **4e**.

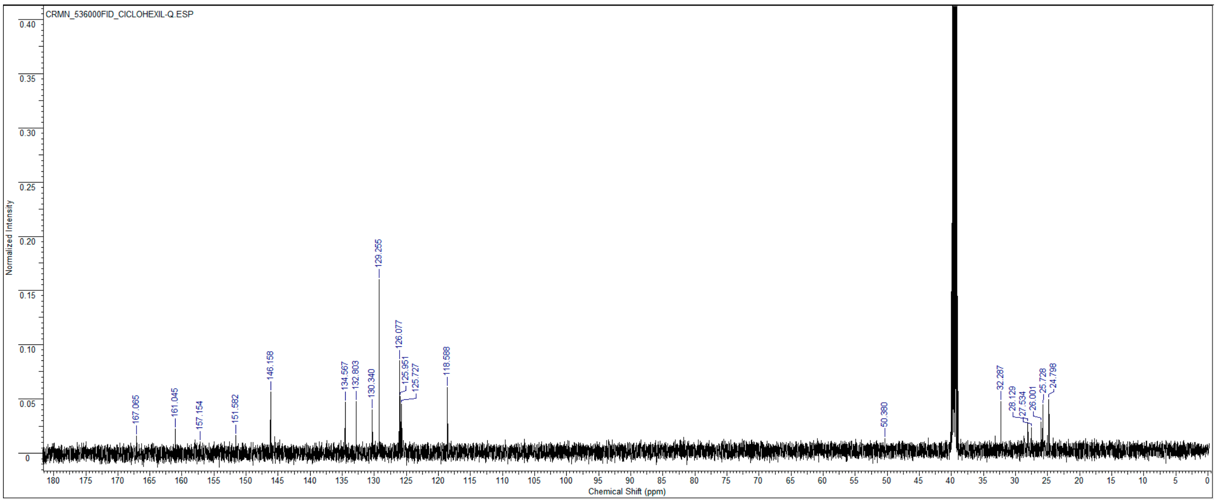

Figure S81. The ^{13}C-NMR spectrum for the compound **4f**.

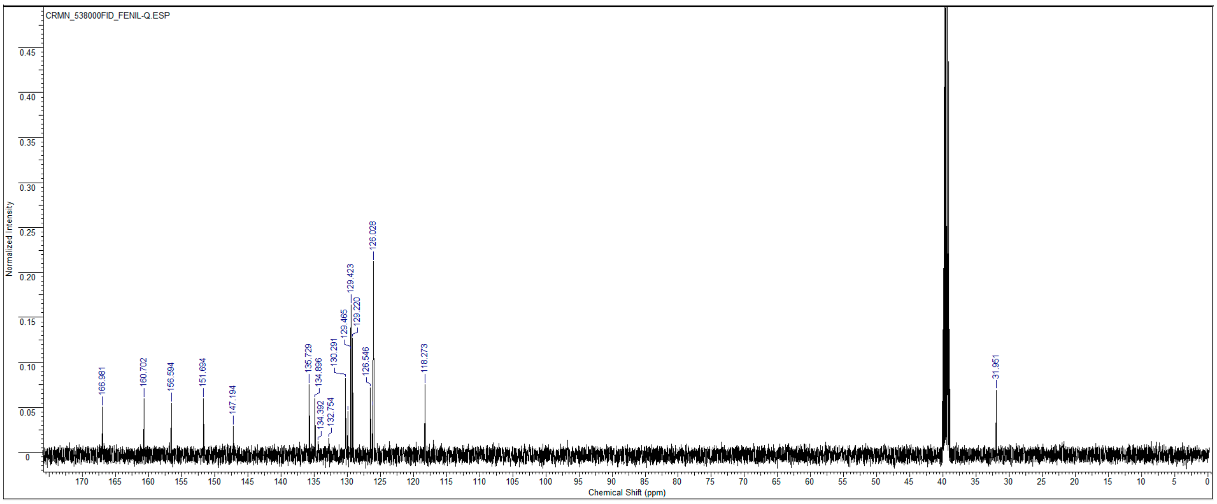

Figure S82. The ^{13}C-NMR spectrum for the compound **4g**.

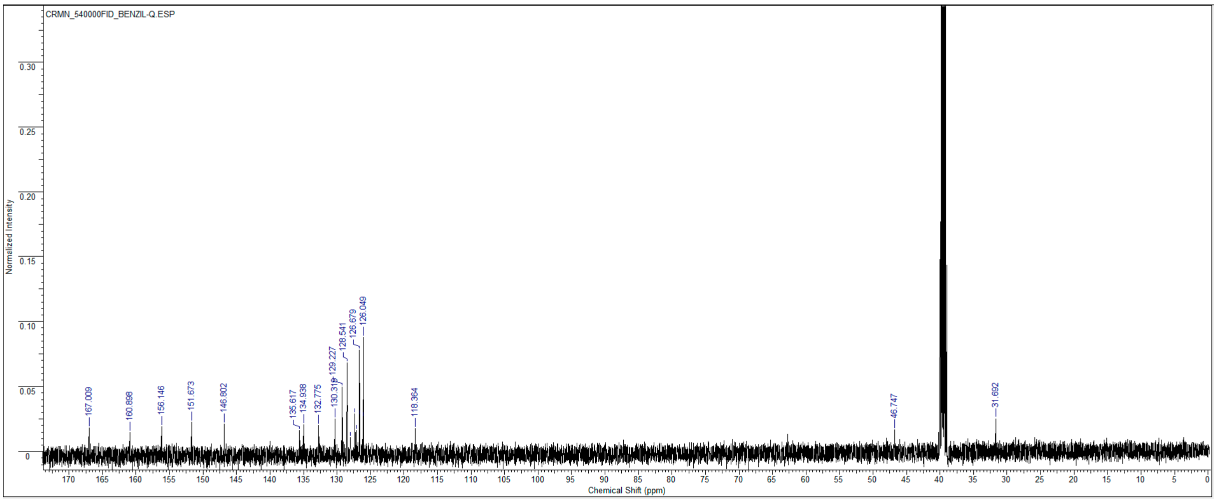

Figure S83. The ^{13}C-NMR spectrum for the compound **4h**.

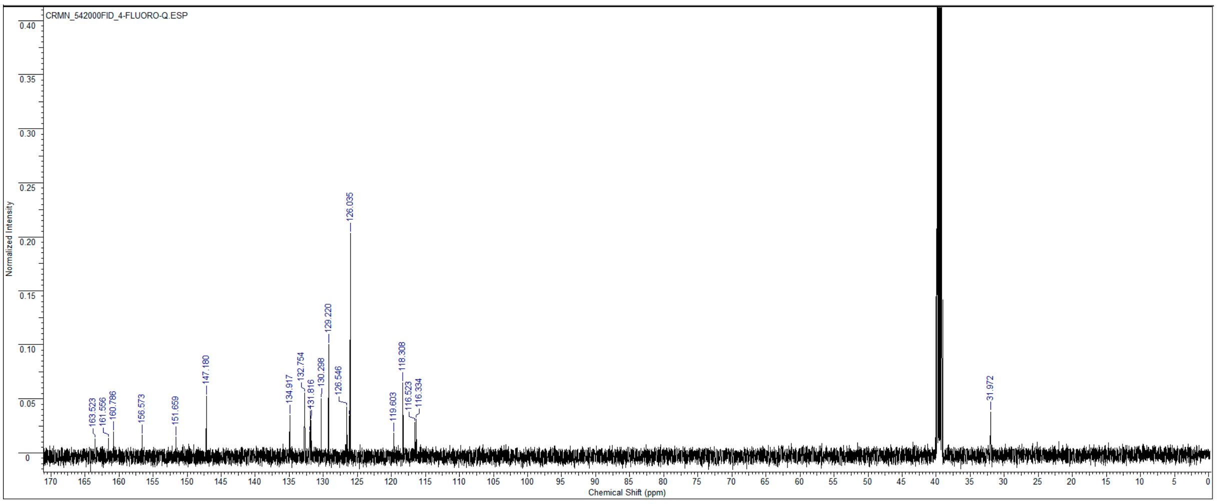

Figure S84. The ^{13}C-NMR spectrum for the compound **4i**.

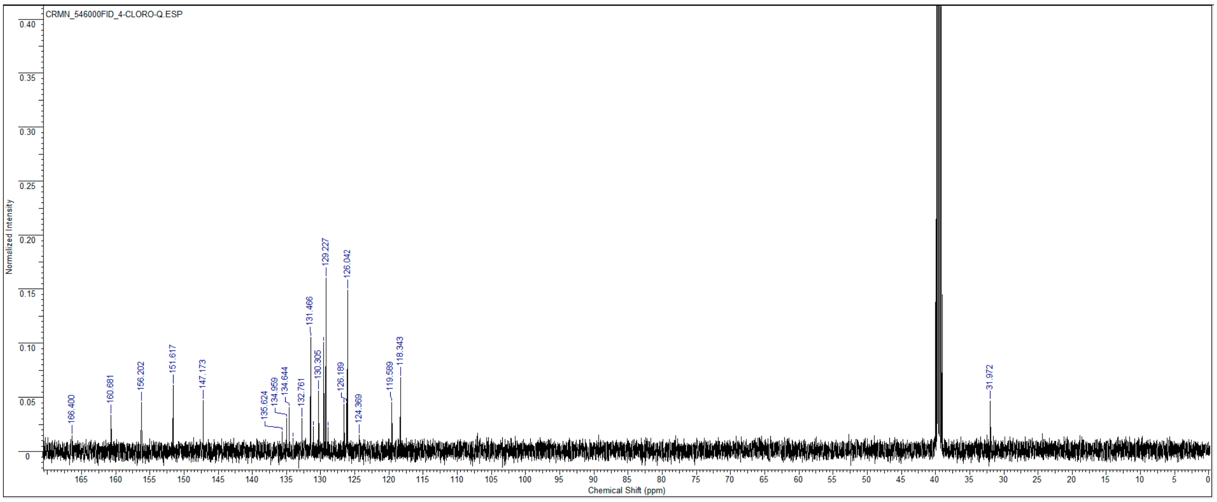

Figure S85. The ^{13}C-NMR spectrum for the compound **4j**.

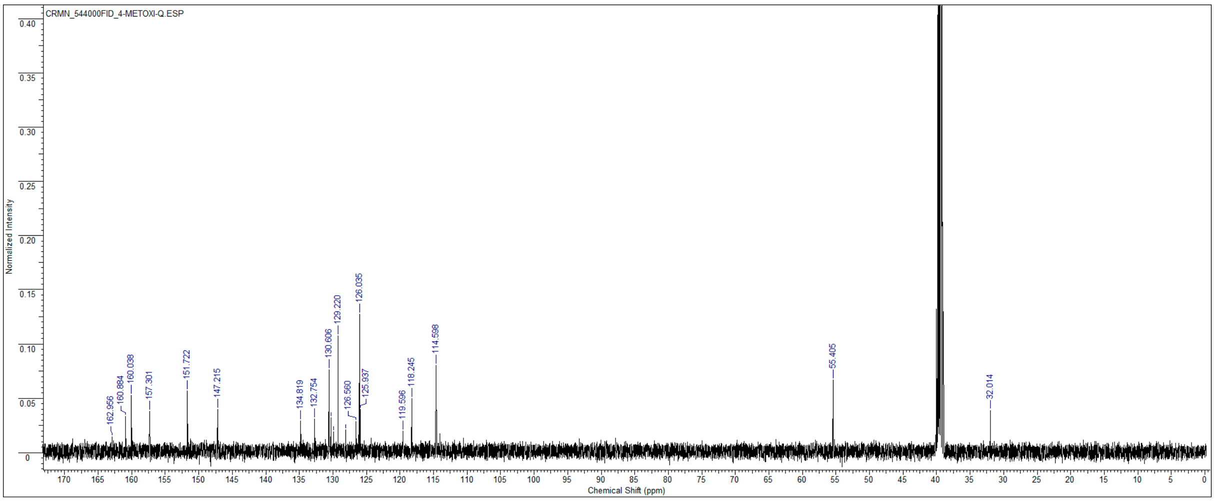

Figure S86. The ^{13}C-NMR spectrum for the compound **4k**.

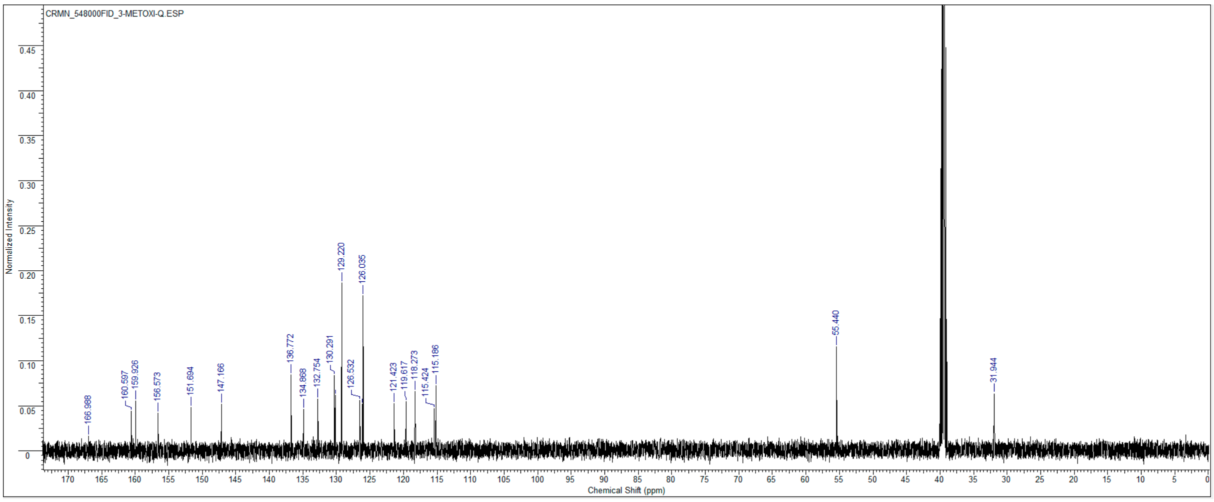

Figure S87. The ^{13}C-NMR spectrum for the compound **4l**.

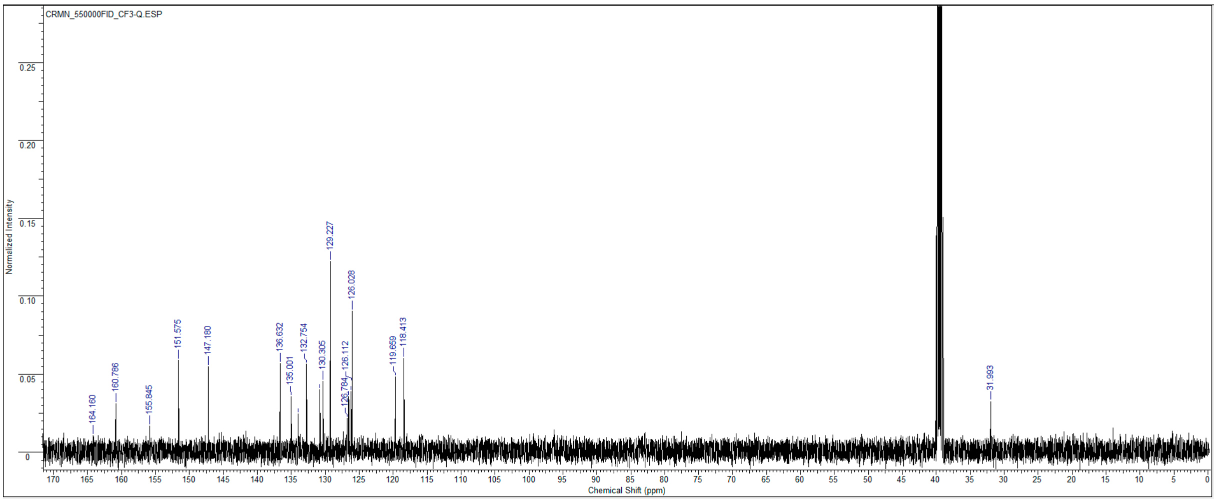

Figure S88. The ^{13}C-NMR spectrum for the compound **4m**.

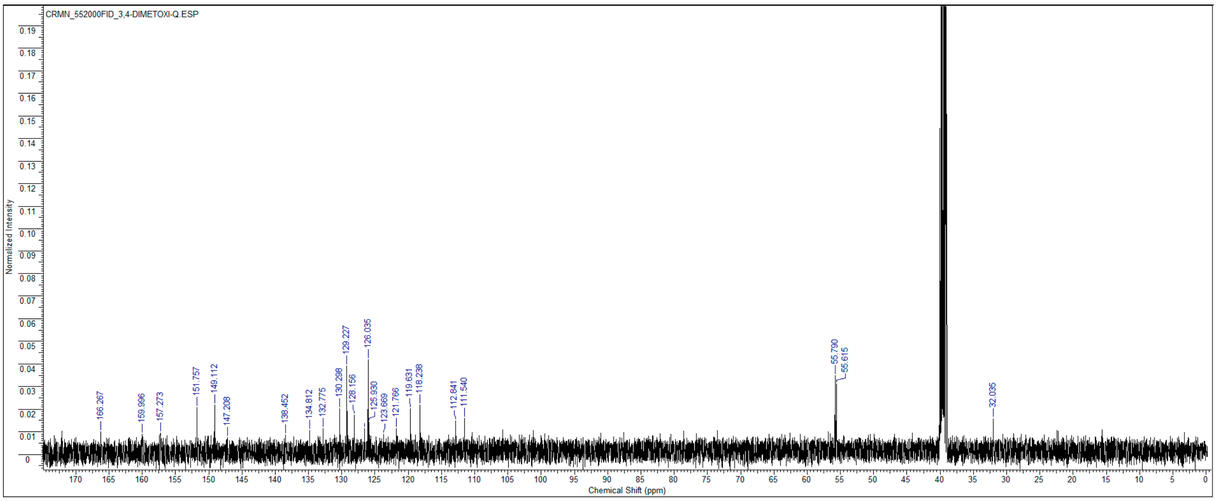

Figure S89. The ^{13}C-NMR spectrum for the compound **4n**.

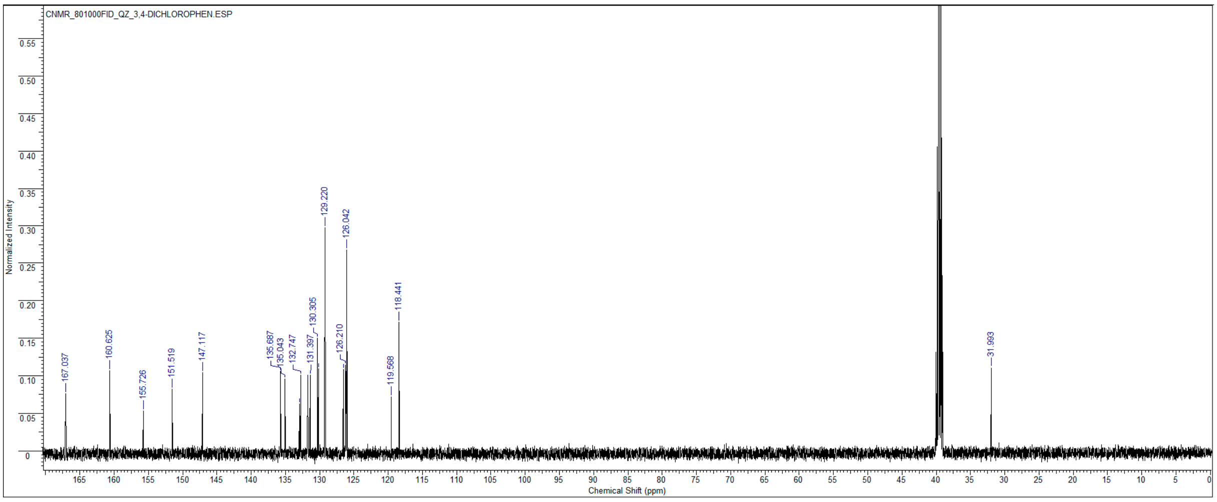

Figure S90. The ^{13}C-NMR spectrum for the compound **4o**.

1.5. Molecular Docking

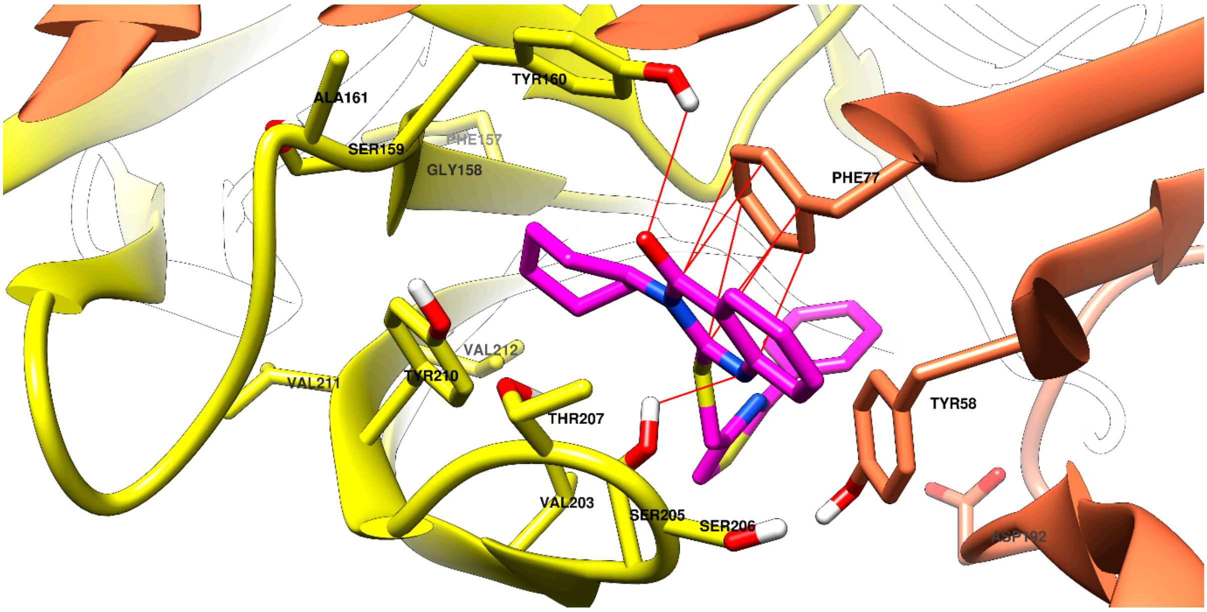

Figure S91. The predicted binding pose of compound **4f** in the human $\alpha_1\beta_2\gamma_2$ GABA$_A$ receptor. The following coloring scheme was used: purple for carbon atoms, red for oxygen atoms, blue for nitrogen atoms, white for hydrogen atoms, and yellow for sulfur atoms.

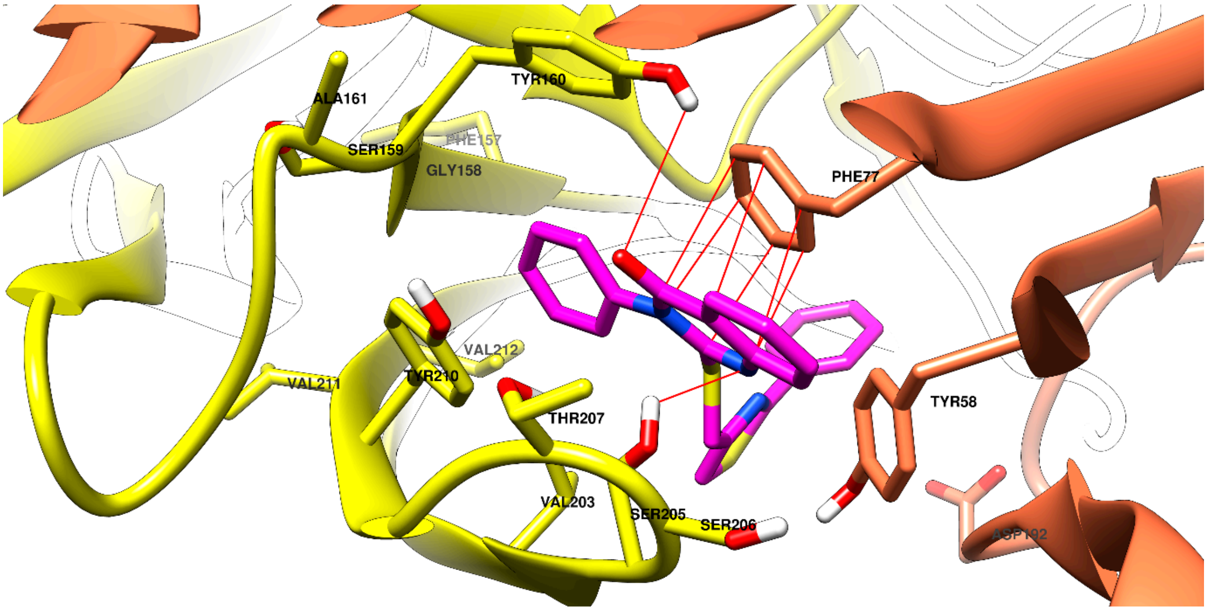

Figure S92. The predicted binding pose of compound **4g** in the human $\alpha_1\beta_2\gamma_2$ GABA$_A$ receptor. The following coloring scheme was used: purple for carbon atoms, red for oxygen atoms, blue for nitrogen atoms, white for hydrogen atoms, and yellow for sulfur atoms.

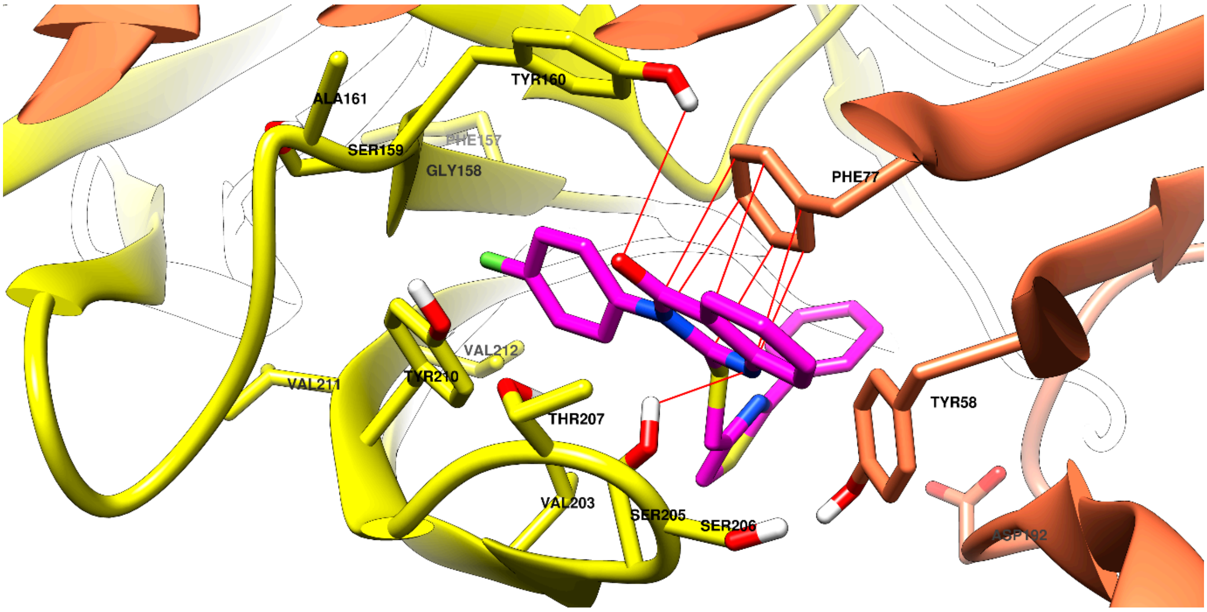

Figure S93. The predicted binding pose of compound **4i** in the human $\alpha_1\beta_2\gamma_2$ GABA$_A$ receptor. The following coloring scheme was used: purple for carbon atoms, red for oxygen atoms, blue for nitrogen atoms, white for hydrogen atoms, and yellow for sulfur atoms.

2. Tables

2.1. ADMETox Prediction Studies

Table S1. The 3D optimized structures of compounds **4a-o**, diazepam, phenobarbital, clomethiazole, methaqualone, and pentylenetetrazole, represented using the ball and stick visualization mode. Color scheme: white – hydrogen atoms, grey – carbon atoms, red – oxygen atoms, blue – nitrogen atoms, yellow – sulfur atoms, neon yellow – fluorine atoms, and green – chlorine atoms.

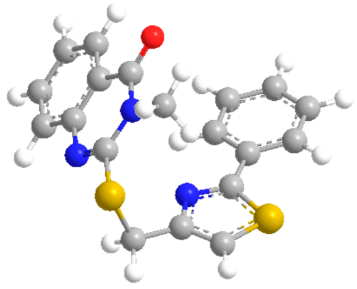 4a	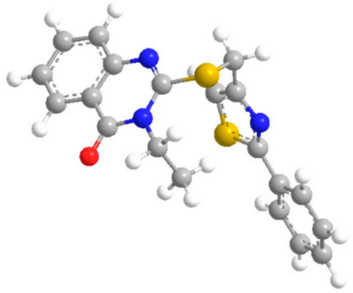 4b	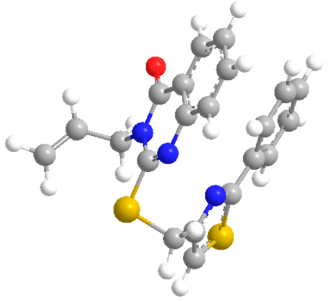 4c
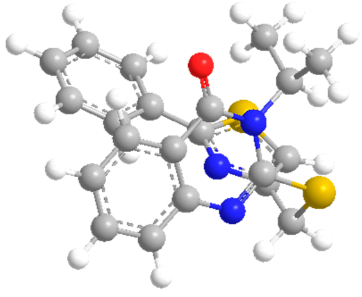 4d	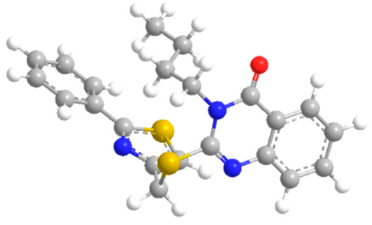 4e	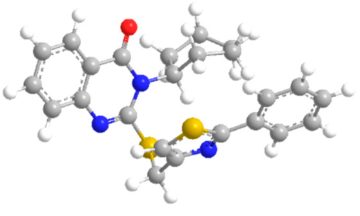 4f
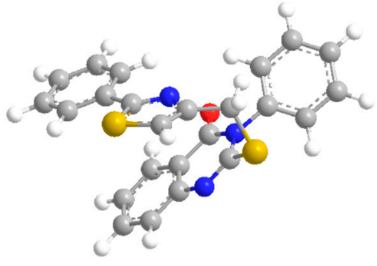 4g	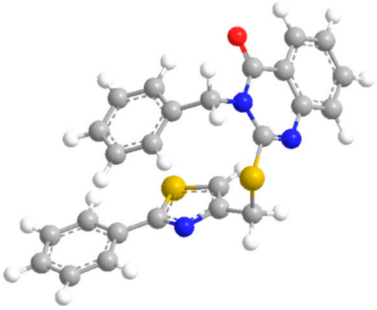 4h	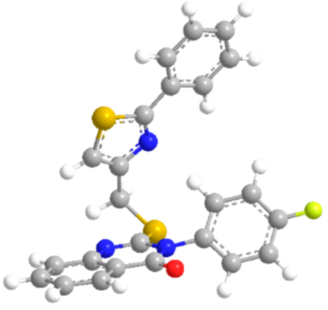 4i
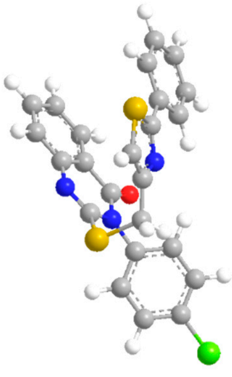 4j	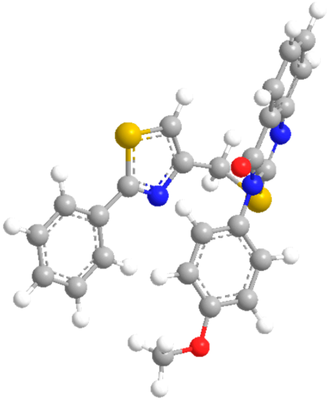 4k	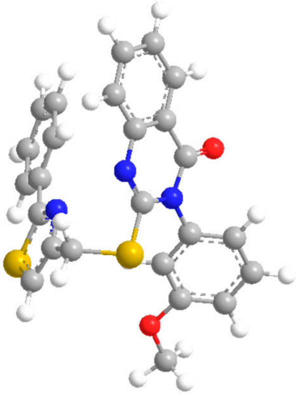 4l
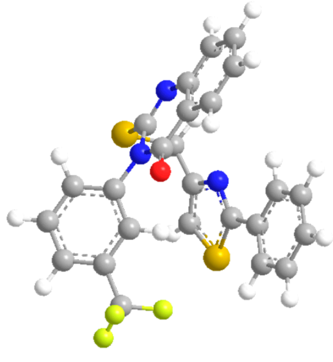 4m	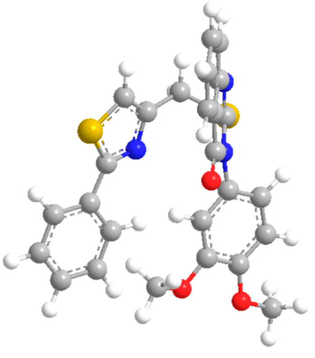 4n	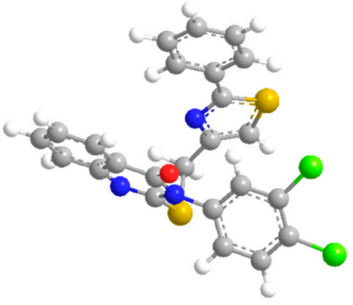 4o

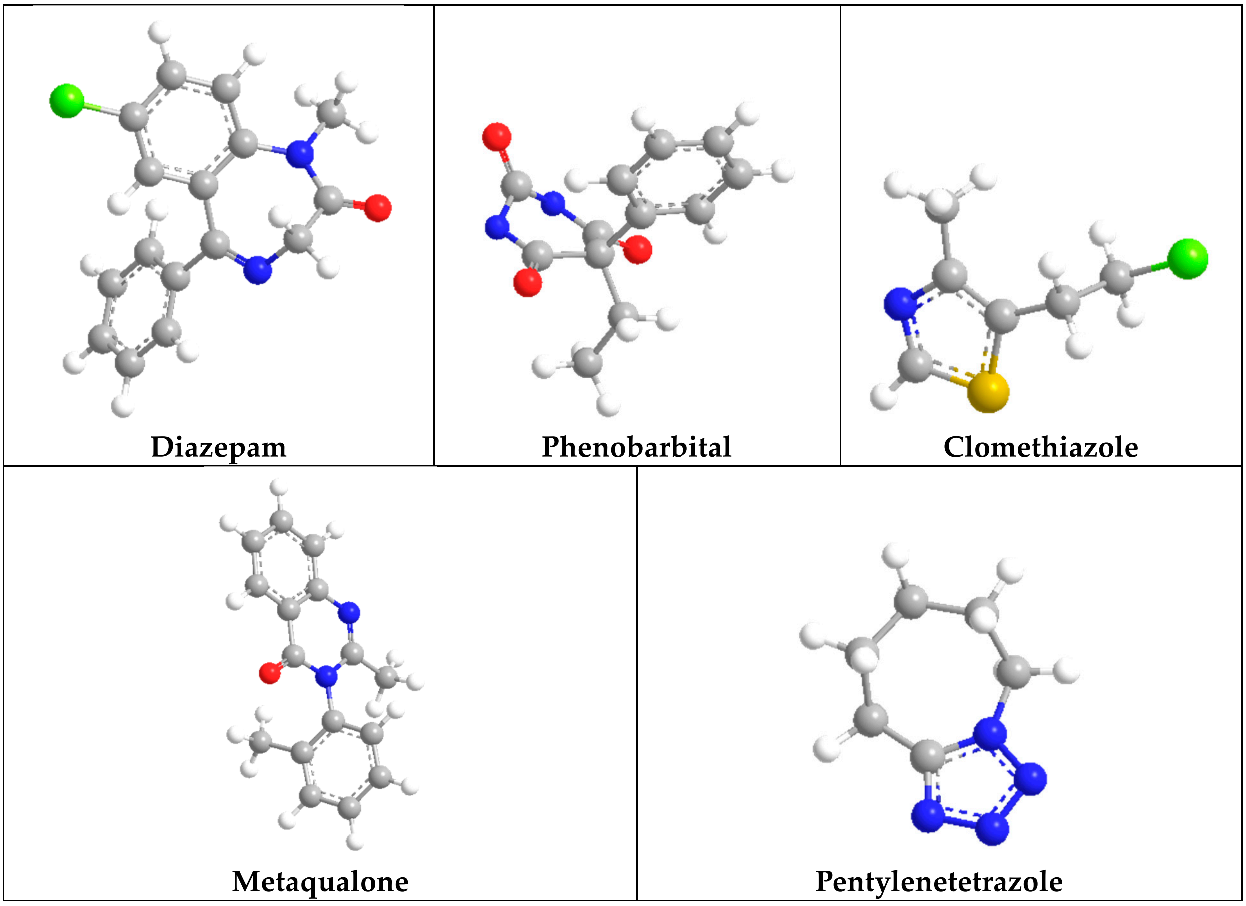

2.2. Anticonvulsant Activity and Neuromotor Coordination Impairment

Table S2. Seizure latency in PTZ-treated mice and statistical comparisons vs. negative control.

Groups	Median (s)	95% Confidence Interval		Adjusted *p* value	Summary
		Lower Bound	Upper Bound		
**NC**	81	64.2	97.8	-	-
**4a**	123	69.0	177.0	0.592	ns
**4b**	99	64.2	133.8	1.000	ns
**4c**	202	0.0	524.9	0.135	ns
**4d**	100	65.2	134.8	0.336	ns
**4e**	137	3.8	270.2	0.118	ns
**4f**	104	0.0	209.6	1.000	ns
**4g**	135	115.8	154.2	0.051	ns
**4h**	178	107.2	248.8	0.078	ns
**4i**	130	102.4	157.6	0.078	ns
**4j**	100	88.0	112.0	1.000	ns
**4k**	145	0.0	332.2	0.135	ns
**4l**	90	85.2	94.8	1.000	ns
**4m**	103	22.6	183.4	1.000	ns
**4n**	132	100.8	163.21	0.068	ns
**4o**	149	85.4	212.6	0.265	ns
**Phenobarbital**	158	59.6	256.4	0.078	ns

**Diazepam**	NR†	-	-	0.009	**

Legend: NR† = Not Reached – complete protection, 0/6 seizures; ** – $p < 0.01$. Mice seizure-free at 30 minutes (1800 seconds) were treated as censored observations.

Data presented as median latency [95% CI]. Groups compared using Kaplan-Meier survival analysis; *p*-values from pairwise log-rank tests (Mantel-Cox) with Bonferroni correction (17 comparisons vs. negative control). Overall group comparison: log-rank test $\chi^2(17) = 66.684$, $p < 0.001$.

Table S3. Mean number of seizures expressed as mean ± standard error of mean (SEM). For the statistically significant values compared to the negative group, the *p* value was expressed as: ***$p < 0.001$; **$p < 0.01$; *$p < 0.05$.

Group	No. seizures
**NC**	12.83 ± 4.094
**4a**	12.83 ± 2.651
**4b**	18.50 ± 3.008
**4c**	6.50 ± 1.708
**4d**	5.167* ± 1.740
**4e**	5.167* ± 1.493
**4f**	6.000 ± 1.211
**4g**	4.667* ± 1.229
**4h**	4.000** ± 0.447
**4i**	2.500** ± 0.5627
**4j**	5.500* ± 0.8062
**4k**	9.000 ± 1.438
**4l**	7.500 ± 1.176
**4m**	7.167 ± 2.056
**4n**	2.833** ± 0.8724
**4o**	4.333* ± 1.202
**Phenobarbital**	4.667* ± 0.4944
**Diazepam**	0.000***

Table S4. Latency until the first seizure (Ti) and mean number of seizures expressed as mean ± standard error of mean (SEM) for compounds **4c** and **4k** in four different doses. For the statistically significant values compared to the negative group, the *p* value was expressed as: ***$p < 0.001$; **$p < 0.01$; *$p < 0.05$.

Group	Ti (s)	No. seizures
**NC**	83.67 ± 8.597	6.333 ± 1.333
	**Compound 4c**	
**D_1 = 50 mg/kg**	160.7 ± 22.48	11.67* ± 0.8433
**D_2 = 150 mg/kg**	443.8 ± 271.7	5.167 ± 1.579
**D_3 = 300 mg/kg**	1069* ± 336.0	1.667* ± 0.7601
**D_4 = 450 mg/kg**	650.0 ± 260.3	3.167 ± 1.352
	**Compound 4k**	
**D_1 = 50 mg/kg**	226.0 ± 145.3	4.000 ± 0.9661
**D_2 = 150 mg/kg**	410.0 ± 279.0	2.667* ± 0.6146
**D_3 = 300 mg/kg**	779.3 ± 307.8	2.833* ± 0.9458
**D_4 = 450 mg/kg**	404.3 ± 96.60	2.333* ± 0.6146

2.3. Flumazenil Antagonism Assay

Table S5. Latency until the first seizure (Ti) and mean number of seizures expressed as mean ± standard error of mean (SEM) for compounds **4c** and **4k** and diazepam in the flumazenil antagonism assay. For the statistically significant values compared to the diazepam group, the *p* value was expressed as: ***$p < 0.001$; **$p < 0.01$; *$p < 0.05$.

Group	Ti (s)	No. seizures
**Diazepam**	126.3 ± 20.54	1.500 ± 0.3416
**Compound 4c**	129.0 ± 19.97	2.667 ± 1.054
**Compound 4k**	132.2 ± 44.25	2.667 ± 0.6146
